# Abstracts from the 10th C1-inhibitor deficiency workshop

**DOI:** 10.1186/s13223-017-0198-5

**Published:** 2017-06-15

**Authors:** Alvin H. Schmaier, Marco Cicardi, Avner Reshef, Dumitru Moldovan, Attila Mócsai, Margarita López-Trascasa, Alberto López Lera, Nancy J. Brown, Anastasios E. Germenis, Rafael Filippelli-Silva, Diego A. Duarte, Renan P. Martin, Camila L. Veronez, Michel Bouvier, Michael Bader, Claudio M. Costa-Neto, João Bosco Pesquero, Xavier Charest-Morin, François Marceau, Georges-É. Rivard, Arnaud Bonnefoy, Éric Wagner, Márta L. Debreczeni, Zsuzsanna Németh, Erika Kajdácsi, Endre Schwaner, László Cervenak, Gábor Oroszlán, András Szilágyi, Ráhel Dani, Péter Závodszky, Péter Gál, József Dobó, Jacques Hébert, Matthieu Vincent, Jean-Nicolas Boursiquot, Hugo Chapdeleine, Marylin Desjardins, Benoit Laramée, Rémi Gagnon, Nancy Payette, Oleksandra Lepeshkina, Delphine Charignon, Arije Ghannam, Denise Ponard, Christian Drouet, Kusumam Joseph, Baby G. Tholanikunnel, Daniel J. Sexton, Allen P. Kaplan, Stefania Loffredo, Maria Bova, Anne Lise Ferrara, Angelica Petraroli, Chiara Suffritti, Nóra Veszeli, Andrea Zanichelli, Henriette Farkas, Gianni Marone, Samuel Luyasu, Bertrand Favier, Ludovic Martin, Kinga Viktória Kőhalmi, György Temesszentandrási, Katalin Várnai, Lilian Varga, Bruce L. Zuraw, Annette Feussner, Michael A. Tortorici, Dipti Pawaskar, Huamin Henry Li, John Anderson, Jonathan A. Bernstein, Ying Zhang, Ingo Pragst, on behalf of COMPACT investigators, Emel Aygören-Pürsün, Kraig Jacobson, Jim Christensen, Arthur Van Leerberghe, Yi Wang, Jennifer Schranz, Inmaculada Martinez-Saguer, Daniel Soteres, Urs Steiner, Vesna Grivcheva Panovska, William Rae, Werner Aberer, Aarnoud Huissoon, Anette Bygum, Markus Magerl, Jochen Graff, Hilary Longhurst, Ramón Lleonart, Lei Fang, Melanie Cornpropst, Desiree Clemons, Amanda Mathis, Phil Collis, Sylvia Dobo, William P. Sheridan, Marcus Maurer, Marc A. Riedl, Timothy Craig, Aleena Banerji, Mustafa Shennak, William Yang, Jovanna Baptista, Paula Busse, Ira Kalfus, Andrew McDonald, Shawn Qian, Anthony Roberts, Con Panousis, Tim Green, Andreas Gille, Maria Zamanakou, Gedeon Loules, Dorottya Csuka, Fotis Psarros, Faidra Parsopoulou, Matthaios Speletas, Davide Firinu, Tiziana Maria Angela De Pasquale, Alessandra Zoli, Anna Radice, Stefano Pizzimenti, Emmanouil Manoussakis, George N. Konstantinou, Valeria Bafunno, Vincenzo Montinaro, Mauro Cancian, Maurizio Margaglione, Konrad Bork, Karin Wulff, Guenther Witzke, Jochen Hardt, Laurence Bouillet, Teresa Caballero, Anete S. Grumach, Christelle Pommie, Irmgard Andresen, Carmen Escuriola Ettingshausen, Zeynep Gutowski, Karin Andritschke, Richard Linde, Noémi Andrási, Tamás Szilágyi, Iris Leibovich-Nassi, Christine Symons, John Dempster, Isabelle Boccon-Gibod, Anne Pagnier, Audrey Lehmann, Kristian B. Kreiberg, Sandra A. Nieto, Raquel Martins, Renata Martins, Alejandra Menendez, Solange O. R. Valle, Margarita Olivares, Maria E. Hernandez-Landeros, Elma Nievas, Natalia Fili, Olga M. Barrera, René Bailleau, Ana Maria Gallardo-Olivos, Masumi Grau, Julian Rodriguez-Galindo, Marlon J. O. Carabantes, Edison Zapata-Venegas, Mario Martinez Alfonso, Maria Rosario-Grauert, Manuel Ratti, Daniel Vaszquez, Dario Josviack, Luis Fernando Landivar-Salinas, Oscar M. E. Calderón-Llosa, Rolando Campilay-Sarmiento, Pablo Raby, Jose Fabiani, William R. Lumry, Henrike Feuersenger, Douglas J. Watson, Thomas Machnig, on behalf of the Investigators of the COMPACT study, Donatella Lamacchia, Adriana Hernanz, Ana Alvez, Mariana Lluncor, Maria Pedrosa, Rosario Cabañas, Nieves Prior, Patrik Nordenfelt, Mats Nilsson, Anders Lindfors, Carl-Fredrik Wahlgren, Janne Björkander, Roman Hakl, Pavel Kuklínek, Irena Krčmová, Jana Hanzlíková, Martina Vachová, Radana Zachová, Marta Sobotková, Jana Strenková, Jiří Litzman, Maria Palasopoulou, Gerasimina Tsinti, Panagiota Gianni, Maria Kompoti, Sofia Garrido, Wojciech Dyga, Anna Bogdali, Aleksander Obtułowicz, Mikolajczyk Tomasz, Ewa Czarnobilska, Krystyna Obtulowicz, Teofila Książek, Anna Koncz, Dominik Gulyás, Maria Staevska, Milos Jesenak, Katarina Hrubiskova, L. Bellizzi, A. Relan, Maddalena A. Wu, Antonio Castelli, Riccardo Colombo, Gianmarco Podda, Marta Del Medico, Emanuele Catena, Francesco Casella, Francesca Perego, Nada Afifi Afifi, Eleonora Tobaldini, Nicola Montano, for the IOS Study Group, Marta Sánchez-Jareño, Marcin Stobiecki, Krystyna Obtułowicz, Irina Guryanova, Ekaterina Polyakova, Viktar Lebedz, Andrej Salivonchik, Svetlana Aleshkevich, Mikhail Belevtsev, Melanie Nordmann-Kleiner, Susanne Trainotti, Janina Hahn, Jens Greve, Liudmyla Zabrodska, Maria L. Oliva Alonso, Rosangela P. Tórtora, Alfeu T. França, Marcia G. Ribeiro, Lisa Fu, Amin Kanani, Gina Lacuesta, Susan Waserman, Stephen Betschel, Melissa I. Espinosa, Francisco A. Contreras, Martin Hrubisko, Ludmila Vavrova, Peter Banovcin, Maryam Ayazi, Mohammad Reza Fazlollahi, Shiva Saghafi, Sajedeh Mohammadian, Susan Nabilou Deshiry, Kiana Bidad, Raheleh Shokouhi Shoormasti, Iraj Mohammadzadeh, Mohammad Hassan Bemanian, Seyed Alireza Mahdaviani, Zahra Pourpak, Anna Valerieva, Mariela Vasileva, Tsvetelina Velikova, Elena Petkova, Vasil Dimitrov, Ruggero Di Maulo, on behalf of participating centers, Raz Somech, Hava Golander, Erika J. Sifuentes, Catherine Mansard, Anne Gompel, Bernard Floccard, Claire Blanchard-Delaunay, David Launay, Olivier Fain, Alain Sobel, Stéphane Gayet, Stéphanie Amarger, Guillaume Armengol, Yann Ollivier, Ariane Zélinsky-Gurung, Pierre-Yves Jeandel, Gisèle Kanny, Brigitte Coppéré, Marie Dubrel, Fabien Pelletier, Aurélie Du Thanh, Sébastien Trouiller, Jérôme Laurent, Claire De Moreuil, Christine Audouin Pajot, Alexandre Belot, Ana Rodríguez, Dasha Roa, Alicia Prieto, Maria Luisa Baeza, Borislava Krusheva, Stephanie K. A. Almeida, Rosemeire N. Constantino-Silva, Nyla Melo, Joanna Araujo Simoes, Sandra Mitie U. Palma, Jane da Silva, Bruna F. de Azevedo, Eli Mansour, Teresa González-Quevedo, Carmen Marcos, Teófilo Lobera, Blanca Sáenz de San Pedro, Ernie Avilla, Jacquie Badiou, Karen Binkley, Rozita Borici-Mazi, Linda Howlett, Paul K. Keith, Anne Rowe, Peter Waite, Aurore Billebeau, Isabelle Boccon-Gibbod, Kristina Lis, Yael Laitman, Eitan Friedman, N. M. Gokmen, O. Gulbahar, H. Onay, Z. P. Koc, A. Z. Sin

**Affiliations:** 1 Case Western Reserve University, University Hospitals Cleveland Medical Center, Cleveland, OH USA; 2 Department of Biomedical and Clinical Sciences Luigi Sacco, University of Milan, ASST Fatebenefratelli Sacco, 20157 Milan, Italy; 3 Angioedema Center, The Sheba Medical Center, 5265601 Tel-Hashomer, Israel; 4 Romanian Network for Hereditary Angioedema, MediQuest Medical Center, Tirgu Mures, Romania; 5 School of Medicine, Semmelweis University, Budapest, Hungary; 6 Unidad de Inmunología, Hospital Universitario La Paz, IdiPAZ, CIBERER U754, Madrid, Spain; 7 Department of Medicine, Vanderbilt University Medical Center, Nashville, TN USA; 8 Department of Immunology & Histocompatibility, School of Health Sciences, Faculty of Medicine, University of Thessaly, Larissa, Greece; 9 Department of Biophysics, Federal University of São Paulo (UNIFESP), São Paulo, São Paulo 04039-032 Brazil; 10 Department of Molecular Biology of Peptide Hormones, Max-Delbrück-Center for Molecular Medicine, Berlin, 13125 Germany; 11 Department of Biochemistry and Immunology, FMRP-USP, Ribeirão Preto, São Paulo 14049-900 Brazil; 12 Department of Biochemistry and Molecular Medicine and Institute for Research in Immunology and Cancer, Université de Montréal, Montreal, QC 6128 Canada; 13 CHU de Québec-Université Laval, Quebec City, QC G1V 4G2 Canada; 14 CHU Ste-Justine, Montreal, QC Canada; 15 3rd Department of Internal Medicine, Semmelweis University, Budapest, Hungary; 16 Institute of Enzymology, Research Centre for Natural Sciences, Hungarian Academy of Sciences, Magyar tudósok krt. 2., Budapest, 1117 Hungary; 17 Centre hospitalier Universitaire de Québec, Université Laval, Quebec, Canada; 18 CHU Ste-Justine, Université de Montréal, Montreal, Canada; 19 Hôpital Charles Lemoyne, Greenfield Park, Canada; 20 Centre Hospitalier Universitaire de Montréal, Université de Montréal, Montreal, Canada; 21 Université McGill, Montreal, Canada; 22 Clinique Médicale 504 Notre-Dame, Repentigny, Canada; 23 KininX SAS, 38000 Grenoble, France; 24 GREPI UE7408, Université Grenoble Alpes, Grenoble, France; 25 CHU, Grenoble, France; 26 Centre de Référence des Angioedèmes (CREAK), Grenoble, France; 27 Department of Biochemistry and Molecular Biology, Medical University of South Carolina, Charleston, SC 29425 USA; 28 Shire Pharmaceuticals, Lexington, MA 02421 USA; 29 Division of Clinical Immunology and Allergy and Center for Basic and Clinical Immunology Research (CISI), University of Naples Federico II, Naples, Italy; 30 Department of Biomedical and Clinical Sciences Luigi Sacco, University of Milan, Milan, Italy; 31 Hungarian Angioedema Center, 3rd Department of Internal Medicine, Semmelweis University, Budapest, 1125 Hungary; 32 CNR Institute of Experimental Endocrinology and Oncology “G. Salvatore”, Naples, Italy; 33 Cliniques du Sud Luxembourg, Arlon, Belgium; 34 Department of Dermatology, Angers University Hospital, Angers, France; 35 Research Laboratory, 3rd Department of Internal Medicine, Semmelweis University, Budapest, 1125 Hungary; 36 Department of Laboratory Medicine, Semmelweis University, Budapest, 1085 Hungary; 37 University of California Medical Center, San Diego, CA USA; 38 Università Degli Studi Di Milano, Milan, Italy; 39 CSL Behring, Marburg, Germany; 40 CSL Behring, King of Prussia, PA USA; 41 Institute for Asthma and Allergy, Chevy Chase, MD USA; 42 Alabama Allergy & Asthma Centre, Birmingham, AL USA; 43 Bernstein Allergy Group, Cincinnati, OH USA; 44 Semmelweis University, Budapest, Hungary; 45 Department for Children and Adolescents, Angioedema Centre, University Hospital Frankfurt, Goethe University, Frankfurt, Germany; 46 Oregon Allergy Associates, Allergy and Asthma Research Group, Eugene, OR USA; 47 University of Medicine & Pharmacy, Mures County Hospital, Tirgu Mures, Romania; 48 Nevada Access to Research & Education Society, Las Vegas, NV USA; 49 Shire, Brussels, Belgium; 50 Shire, Lexington, MA 02421 USA; 51 Hemophilia Center Rhein Main, Mörfelden-Walldorf, Germany; 52 Asthma & Allergy Associates, Colorado Springs, CO USA; 53 Department of Clinical Immunology, University Hospital Zurich, Zurich, Switzerland; 54 PHI University Clinic of Dermatology, Skopje, Republic of Macedonia; 55 The University Hospital Southampton NHS Foundation Trust, Southampton, UK; 56 Medizinische Universität Graz, Graz, Austria; 57 Heart of England NHS Foundation Trust, Birmingham, UK; 58 JHAE Centre Denmark, Odense University Hospital, Odense, Denmark; 59 Department of Dermatology and Allergy, Allergie-Centrum-Charité, Charité-Universitätsmedizin Berlin, Berlin, Germany; 60 Projektgruppe Translationale Medizin & Pharmakologie TMP, Fraunhofer-Institut für Molekularbiologie und angewandte Oekologie IME, Frankfurt, Germany; 61 Department of Immunology, Barts Health NHS Trust, London, E1 2ES UK; 62 Hospital Universitari de Bellvitge, Barcelona, Spain; 63 PharStat, Inc., Durham, NC USA; 64 BioCryst Pharmaceuticals, Durham, NC USA; 65 University of California-San Diego School of Medicine, La Jolla, CA 92122 USA; 66 Department of Internal Medicine/Allergy Section Cincinnati, University of Cincinnati College of Medicine, Cincinnati, OH 45267 USA; 67 Department of Medicine and Pediatrics, Penn State University Allergy, Immunology and Respiratory Research, Penn State Hershey Allergy, Asthma and Immunology, Hershey, PA 17033 USA; 68 Division of Rheumatology, Allergy and Immunology, Department of Medicine, Massachusetts General Hospital, Harvard Medical School, Boston, MA 02114 USA; 69 Luigi Sacco Hospital Milan, Milan, Italy; 70 Triumpharma Inc., Amman, Jordan; 71 Ottawa Allergy Research Corporation, University of Ottawa Medical School, Ottawa, ON K1G 6C6 Canada; 72 Division of Clinical Immunology and Allergy, Department of Medicine, Icahn School of Medicine at Mount Sinai, New York, NY 10029 USA; 73 Attune Pharmaceuticals, LLC, New York City, NY 10019 USA; 74 Global Research, CSL Limited, Parkville, VIC Australia; 75 CeMIA SA, Larissa, Greece; 76 Department of Translational Medical Sciences, University of Naples Federico II, Naples, Italy; 77 Department of Allergology, Navy Hospital, Athens, Greece; 78 Department of Medical Sciences and Public Health, University of Cagliari, Cagliari, Italy; 79 Department of Allergy, Civitanova Marche Hospital, Civitanova Marche, Italy; 80 Department of Clinical Immunology, Ospedali Riuniti, Ancona, Italy; 81 Unit of Immunoallergology, Azienda Ospealiera Universitarta Careggi, Florence, Italy; 82 Department of Allergy, University of Torino, Turin, Italy; 83 Allergy Unit, 2nd Pediatric Clinic, University of Athens, “P & A Kyriakou” Children’s Hospital, Athens, Greece; 84 Department of Allergy and Clinical Immunology, 424 General Military Training Hospital, Thessaloníki, Greece; 85 Medical Genetics, Department of Clinical and Experimental Medicine, University of Foggia, Foggia, Italy; 86 Division of Nephrology, University of Bari, Bari, Italy; 87 Department of Medicine, University of Padua, Padua, Italy; 88 Department of Dermatology, University Mainz, Mainz, Germany; 89 University Medicine, Ernst Moritz Arndt University, Greifswald, Germany; 90 Department of Medical Psychology and Medical Sociology, University Mainz, Mainz, Germany; 91 Department of Dermatology and Venereology, Medical University of Graz, Graz, Austria; 92 National Reference Centre for Angioedema, Internal Medicine Department, Grenoble University Hospital, CHU Grenoble Alpes, Université Grenoble Alpes, Grenoble, France; 93 Allergy Department, Hospital La Paz Institute for Health Research (IdiPaz), Biomedical Research Network on Rare Diseases (CIBERER, U754), Madrid, Spain; 94 Department of Dermatology and Allergy Centre, Odense University Hospital, Odense, Denmark; 95 Faculty of Medicine ABC, São Paulo, Brazil; 96 Shire, Zug, Switzerland; 97 Haemophilia Centre Rhine Main, Frankfurt-Mörfelden, Germany; 98 Angioedema Research Center, Sheba Medical Center, 5265601 Tel-Hashomer, Israel; 99 Rhein-Main Haemophilia Centre (HZRM), Moerfelden, 64546 Germany; 100 Derriford Hospital, Plymouth, PL6 8DH UK; 101 Barts HealthWhitechapel, London, E1 1BB UK; 102 CHUGA, Grenoble, France; 103 HAE Centre Denmark, Department of Dermatology and Allergy Centre, Odense University Hospital, Odense, Denmark; 104 Clinical Immunology, ABC School of Medicine, Santo Andre, SP Brazil; 105 National Institute of Pediatrics, Mexico City, Mexico; 106 Mexican Association of Patients with Hereditary Angioedema, Mexico, Mexico; 107 Brazilian Association of Patients with Hereditary Angioedema (ABRANGHE), São Paulo, Brazil; 108 Association of Patients with Hereditary Angioedema from Argentina, Buenos Aires, Argentina; 109 Federal University of Rio de Janeiro, Rio de Janeiro, Brazil; 110 Group of Clinical and Experimental Allergology (GACE), Medellín, Colombia; 111 Medicina Clinica del Este “Rosa Rangel”, Xalapa, Veracruz Mexico; 112 Allergy Clinic, Buenos Aires, Argentina; 113 Allergist Salta Capital, Salta, Argentina; 114 Jefa de la Clínica de Alergias e Inmunología del Instituto de Neumologia y Alergias del Hospital San Fernando, Panama, Panama; 115 Clinics of Allergy, Buenos Aires, Argentina; 116 Hospital Barros Luco Trudeau, Santiago, Chile; 117 Hospital El Pino, University of Santiago, Santiago, Chile; 118 San Pablo Clinic, Lima, Peru; 119 Allergy and Clinical Immunology, Hospital Nacional Rosales, San Salvador, El Salvador; 120 Hospital Quito N.1, National Police of Equador, Quito, Ecuador; 121 Clinics of Allergy, San Jose, Costa Rica; 122 Allergy Clinic, Montevideo, Uruguay; 123 Allergy Clinic, Assuncion, Paraguay; 124 Allergy Sevice “Clinica Estrada”, Buenos Aires, Argentina; 125 Institute of Respiratory Medicine, Rafaela, Santa Fe Argentina; 126 Clinical Allergist, Santa Cruz, Bolivia; 127 Medical Center San Sebastian, Piura, Peru; 128 Researcher in Hereditary Angioedema at the Argentinian Institute of Allergy and Founder of Latin American Association of Hereditary Angioedema (ALAeh), Buenos Aires, Argentina; 129 AARA Research Center, Dallas, TX 75231 USA; 130 Department of US HAEA Angioedema Center, UC San Diego, La Jolla, CA 92193 USA; 131 Department of Internal Medicine, Hungarian Angioedema CenterSemmelweis University, 1085 Budapest, Hungary; 132 CSL Behring GmbH, 35002 Marburg, Germany; 133 Department of Translational Medical Sciences, Center of Basic and Clinical Immunology Research, University of Naples Federico II, Naples, Italy; 134 Department of Allergy, Hospital La Paz Institute for Health Research (IdiPaz), Madrid, Spain; 135 Department of Clinical and Experimental Medicine, University of Linköping, Linköping, Sweden; 136 Department of Internal Medicine, County Hospital Ryhov, Jönköping, Sweden; 137 Futurum-Academy for Health and Care, Region Jönköping County, Jönköping, Sweden; 138 Department of Pediatrics, Astrid Lindgren Children’s Hospital, Karolinska University Hospital, Stockholm, Sweden; 139 Dermatology Unit, Department of Medicine Solna, Karolinska Institutet, Stockholm, Sweden; 140 Department of Dermatology, Karolinska University Hospital, Stockholm, Sweden; 141 Department of Clinical Immunology and Allergology, St. Anne’s University Hospital in Brno, Masaryk University, Brno, Czech Republic; 142 Department of Clinical Immunology and Allergology, University Hospital in Hradec Králové, Charles University, Hradec Kralove, Czech Republic; 143 Department of Immunology and Allergology, University Hospital in Plzeň, Charles University, Plzeň, Czech Republic; 144 Department of Immunology, University Hospital in Motol, Charles University, Motol, Czech Republic; 145 Institute of Biostatistics and Analyses at the Faculty of Medicine and the Faculty of Science of the Masaryk University, Brno, Czech Republic; 146 Allergy and Lung Function Office, University of Medicine and Pharmacy, Tirgu Mures, Romania; 147 Department of Clinical and Environmental Allergology, Jagiellonian University Medical College, Sniadeckich 10, 31-531 Kraków, Poland; 148 Department of Dermatology, Jagiellonian University Medical College, Skawinska 8, Kraków, Poland; 149 Department of Internal and Agricultural Medicine, Jagiellonian University Medical College, Kraków, Poland; 150 Department of Medical Genetics, Faculty of Medicine, Jagiellonian University Medical College, Kraków, Poland; 151 St. Anne’s University Hospital of Brno, Brno, Czech Republic; 152 University Hospital “Alexandrovska” Medical University of Sofia, Sofia, Bulgaria; 153 University Hospital of Martin, Martin, Slovakia; 154 Bratislava University Hospital, Bratislava, Slovakia; 155 Pharming Technologies BV, Leiden, The Netherlands; 156 Intensive Care Unit, ASST Fatebenefratelli Sacco, Luigi Sacco Hospital, Milan, Italy; 157 Medicina III, Azienda Ospedaliera San Paolo, Dipartimento di Scienze della Salute, Università degli Studi di Milano, Milan, Italy; 158 Allergy & Immunology, Angioedema Center, ShebaMedical Center, Tel-Hashomer, Israel; 159 Department of Biomedical and Clinical Sciences, University of Milan, Milan, Italy; 160 Department of Clinical Sciences and Community Health, IRCCS Ca’ Granda Foundation, Ospedale Maggiore Policlinico, University of Milan, Milan, Italy; 161 Clinical Immunology, Faculty of Medicine ABC, Santo André, SP Brazil; 162 Department of Allergy, Instituto de Investigación Hospital Universitario La Paz (IdiPaz), Madrid, Spain; 163 CIBERER, U754, Madrid, Spain; 164 Medical College, Jagiellonian University, Kraków, 31-531 Poland; 165 Belorusian Research Center for Pediatric Oncology, Hematology and Immunology, Borovlyani, Minsk region 223053 Belarus; 166 Department of Otorhinolaryngology Head and Neck Surgery, Ulm University Medical Center, Ulm, 89075 Germany; 167 Centre of Allergic Diseases of Upper Airways, Institute of Otolaryngology by Name of O.S. Kolomiychenko of the National Academy of Medical Sciences of Ukraine, Kyiv, 03057 Ukraine; 168 Division of Clinical Immunology and Allergy, Department of Medicine, University of Toronto, Toronto, ON M5B 1W8 Canada; 169 Division of Allergy and Immunology, Department of MedicineUniversity of British Columbia, Vancouver, BC Canada; 170 Department of Medicine, Dalhousie University, Halifax, NS B3H 4R2 Canada; 171 Division of Clinical Immunology Allergy, McMaster University, Hamilton, ON Canada; 172 Allergy and Clinical Immunology, Allergy DepartmentInstituto Nacional de Pediatría, Mexico City, Mexico; 173 Clinical Allergy Department, Instituto Nacional de Pediatría, Mexico City, Mexico; 174 Nutritional Genetics Department, Instituto Nacional de Pediatría, Mexico City, Mexico; 175 Asociación Mexicana de Angioedema Hereditario, Mexico City, Mexico; 176 Center for Hereditary Angioedema, Comenius University in Bratislava, Jessenius Faculty of Medicine, University Teaching Hospital, Martin, Slovakia; 177 Center for Hereditary Angioedema, Comenius University in Bratislava, Faculty of Medicine, University Teaching Hospital, Bratislava, Slovakia; 178 Department of Clinical Immunology and Allergology, St. Elisabeth’s Oncology Institute, Bratislava, Slovakia; 179 Department of Medical Genetics, St. Elisabeth’s Oncology Institute, Bratislava, Slovakia; 180 Immunology, Asthma and Allergy Research InstituteTehran University of Medical Sciences, Tehran, Iran; 181 Non-Communicable Pediatric Diseases Research Center, Babol University of Medical Sciences, Babol, Iran; 182 Department of Clinical Immunology and Allergy, Hazrate Rasoul Hospital, Iran University of Medical Sciences, Tehran, Iran; 183 Pediatric Respiratory Diseases Research Center, National Research Institute of Tuberculosis and Lung Diseases (NRITLD), Shahid Beheshti University of Medical Sciences, Tehran, Iran; 184 Clinic of Allergy and Asthma, Medical University of Sofia, University Hospital “Alexandrovska”, Sofia, Bulgaria; 185 Department of Surgery, Medical University of Sofia, University Hospital “Alexandrovska”, Sofia, Bulgaria; 186 Laboratory of Clinical ImmunologyUniversity Hospital St. Ivan Rilski, Medical University-Sofia, Sofia, Bulgaria; 187 CEO Cloud-R s.r.l., Milan, Italy; 188 Safra Pediatric Hospital, Sheba Medical Center, 5265601 Tel-Hashomer, Israel; 189 Department of Nursing, Sackler School of Medicine, Ramat Aviv, 6997801 Israel; 190 Mexican Association of Hereditary Angioedema, Mexico City, Mexico; 191 CREAKCHU GrenobleMédecine Interne, GRENOBLE, France; 192 Department of Internal Medicine, Grenoble University Hospital, Grenoble, France; 193 National Reference Centre for Angioedema, CREAK, Grenoble, France; 194 Endocrine Gynecology, Paris Descartes University, University Hospital Centre, Paris, France; 195 Intensive Care Unit, Edouard Herriot Hospital, Hospices Civils de Lyon, Lyon, France; 196 Department of Internal Medicine, CH Niort, Niort, France; 197 Université Lille Nord de France, Department of Internal Medicine, National Reference Centre for Autoimmune and Systemic Rare Diseases, Hospital Claude-Huriez, CHRU Lille, Lille, France; 198 Department of Internal Medicine, Saint Antoine University Hospital, AP-HP, Paris, France; 199 Hotel Dieu University Hospital, AP-HP, Paris, France; 200 Department of Internal Medicine, La Timone Hospital, Marseille, France; 201 Department of Dermatology and Cutaneous Oncology, CHU Clermont-Ferrand, Clermont-Ferrand, France; 202 Department of Internal Medicine, Bois-Guillaume Hospital, CHU Rouen, Bois-Guillaume, France; 203 Department of Internal Medicine, CHU Caen, Caen, France; 204 Department of Pediatric Emergency, CH Niort, Niort, France; 205 Department of Internal Medicine, CHU Nice, Nice, France; 206 Department of Internal Medicine and Clinical Immunology, CHU Nancy, Nancy, France; 207 Department of Internal Medicine, Edouard Herriot Hospital, Hospices Civils de Lyon, Lyon, France; 208 Department of Internal Medicine-Pediatry, Saint Antoine University Hospital, AP-HP, Paris, France; 209 Department of Dermatology, Jean Minjoz Hospital, CHU Besançon, Besançon, France; 210 Department of Dermatology, CHU Montpellier, Montpellier, France; 211 Department of Internal Medicine, Henri-Mondor Hospital, CH Aurillac, Aurillac, France; 212 Department of Allergology, HEGP Hospital, AP-HP, Paris, France; 213 Department of Internal Medicine, CHU Brest, Brest, France; 214 Department of Internal Medicine, CHU Toulouse, Toulouse, France; 215 Department of Pediatric Internal Medicine, Mother Children Hospital, Hospices Civils de Lyon, Lyon, France; 216 Department of Allergy, Hospital General Universitario Gregorio Marañón, Madrid, Spain; 217 Clinic of Allergy and Asthma, Medical University of Sofia, Sofia, Bulgaria; 218 Laboratory of Biophysics, Federal University of Sao Paulo, Sao Paulo, SP Brazil; 219 Serviço de Alergia, University Hospital Professor Polydoro Ernani de São Thiago, Federal University of Santa Catarina, Florianópolis, SC Brazil; 220 Department of Clinical Immunology, Federal University of Rio de Janeiro, Rio de Janeiro, RJ Brazil; 221 Department of Clinical Allergy and Immunology, Faculty of Medicine, State University of Campinas, Campinas, SP Brazil; 222 Outpatient Group of Recurrent Infections and Laboratory of Clinical Immunology, Faculty of Medicine ABC, Santo André, SP Brazil; 223 Department of Allergy, Hospital Universitario Virgen del Rocío, Sevilla, Spain; 224 Department of Allergy, Complejo Hospitalario Universitario de Vigo, Vigo, Spain; 225 Department of Allergy, Hospital San Pedro, Logroño, Spain; 226 CIBERER U761, Madrid, Spain; 227 Department of Allergy, Hospital Universitario de Jaén, Jaén, Spain; 228 IDEA Group, Hamilton, On L8S 3C2 Canada; 229 HAE Canada, Ottawa, ON K1Y 4G2 Canada; 230 Division of Allergy and Immunology Queens University, Kingston, ON K7L 5G2 Canada; 231 Centre de Recherche Appliquée en Allergie de Québec, Quebec, G1V 4W2 Canada; 232 Division of Allergy & Clinical Immunology, St. Paul’s Hospital, Vancouver, BC V6Z 1Y6 Canada; 233 Division of Clinical Immunology and Allergy, Department of Medicine, McMaster University, Hamilton, ON L8S 4K1 Canada; 234 Canadian Hereditary Angioedema Network, Toronto, Ontario M5B 2H5 Canada; 235 Department of Endocrinology Gynecology, Paris Descartes University, 75014 Paris, France; 236 Department of Internal Medicine, Paris Pierre et Marie Curie University, 75012 Paris, France; 237 Department of Internal Medicine, Lille Nord de France University, 59000 Lille, France; 238 Department of Internal Medicine, Grenoble Alpes University, 38400 Grenoble, France; 239 French National Reference Center for Hereditary Angioedema (CREAK), Grenoble, France; 240 Laboratory of Molecular Genetics, Institute of Human Genetics, The Sheba Medical Center, 5265601 Tel-Hashomer, Israel; 241 Immunology, Ege University, Izmir, 34437 Turkey

## Preface

The 10th C1-inhibitor deficiency workshop will be held between 18 and 21 May 2017 in Budapest (2017.haenetworkshop.hu), among the picturesque surroundings of Margaret Island. As indicated by the name of this event, most of the interest focused on angioedema due to C1-inhibitor deficiency in 1999, when it was first organized. The name is unchanged, but the range of angioedemas has expanded since to include all known varieties of hereditary and acquired angioedemas with a bradykinin-mediated pathomechanism. Looking back to the agenda of this biennial conference, many questions remained unanswered and new issues have arisen despite the enormous scientific progress made.

On this occasion, 318 participants have registered from 42 countries—this is the greatest attendance in the history of the Workshop since the start of the series. Eighty-six presentations have been submitted for this 4-day long scientific forum. The scientific program sounds interesting—it comprises novel achievements by leading scientific teams in basic research into bradykinin-mediated angioedema, the new findings of diagnostics and genetics, promising therapeutic solutions awaiting introduction, and the experience accumulated with the latest therapeutic procedures. Several presentations discuss the efforts related to improving the patients’ quality of life. This time, we have invited five prominent experts—namely, Alvin Schmaier (Cleveland, OH, USA), Marco Cicardi (Milan, Italy), Avner Reshef (Tel-Hashomer, Israel), Dumitru Moldovan (Tirgu-Mures, Romania) and Attila Mócsai (Budapest, Hungary). Alvin Schmaier will show us that our knowledge about the underlying mechanisms of bradykinin-mediated angioedemas is still limited—this may be remedied by extending our interest to other forms of angioedema with different pathophysiological backgrounds. Marco Cicardi will expose the similarities and the differences between bradykinin-mediated edema formation, and the idiopathic systemic capillary leak syndrome. Avner Reshef will explore a similar issue in his presentation titled ‘*Angioedema*–*Histamine or Bradykinin*?’. The lecture on neutrophil granulocytes by Attila Mócsai will take us closer to understanding the pathomechanism of angioedema. The agenda also contains the traditional roundtable session, an opportunity to develop consensus and international guidelines—this year, genetics will be in the limelight. In this session, the keynote lectures will be read by Margarita-Lopez Trascasa (Madrid, Spain) on the extended diagnostic approach integrating serological and genetic methodology; by Anastasios Germenis (Larissa, Greece) on the latest techniques for studying the *SERPING1* gene; and by Nancy Brown (Nashville, TN, USA) on the pharmacogenetics of angiotensin-converting enzyme inhibitor-associated angioedema. Notwithstanding the remarkable progress made in South-America and in the former Soviet-bloc countries of Europe, state-of-the-art diagnostic and therapeutic modalities are still not available in many regions of the World. Dumitru Moldovan will review the stages along the way to making these accessible, and the experience accumulated in the effort to achieve high levels of patient care.

The conference will be attended both by researchers and by clinicians—medical professionals and nurses, by the representatives of patient organizations, and by pharmaceutical industry experts involved in drug development, in order to assist the efforts of each other through joint thinking. Within the framework of this fruitful cooperation, the pharmaceutical companies also lent financial support to the conference—in addition to their scientific contribution. The travel grants, make it possible for an increasing number of professionals involved in the research or the management of patients with angioedema to attend the Workshop.

The generous support by our Sponsors enabled us again to present the “*For HAE Patients*” award, as well as the “*Grant for Young Investigators*”. The major donors to this event are CSL Behring and Shire. Pharming Group NV, Swedish Orphan Biovitrum, BioCryst Phamaceuticals, KininX SAS also contributed the sponsorship of the Workshop. On occasion of this tenth, jubilee event, the “*For HAE Patients*” award goes to Bruce Zuraw (San Diego, USA), who will present his lecture ‘*Let the Treatment Fit the Disease*’ on the festive session of the scientific section on Day 1. His achievements in bettering the management of patients will be recalled by Antony Castaldo, the chair of the International Patient Organization for C1 Inhibitor Deficiencies. The concluding event of the conference will be the awarding of the ‘*Grant for Young Investigators’* to the top four young presenters.

The support referred to above made it possible to publish the submitted abstracts of the Workshop in the journal *Allergy, Asthma, and Clinical Immunology*, in order to make them available to an even broader range of professionals interested in this subject.

## Invited lectures

### I-1 C1 inhibitor—where we came from to where we are going

#### Alvin H. Schmaier

##### Case Western Reserve University, University Hospitals Cleveland Medical Center, Cleveland, OH, USA

###### **Correspondence:** Alvin H. Schmaier (schmaier@case.edu)

*Allergy, Asthma & Clinical Immunology* 2017,** 13(Suppl 2)**:I-1

C1 inhibitor (C1INH) is a SERPIN, serine protease inhibitor, which is the major regulator of activated forms of factor XII, the first component of complement, and accounts for 50% of plasma kallikrein inhibition. It is also regulates factor XIa, although clinically is not as important as alpha-1-antitrypsin and antithrombin. As a SERPIN, C1INH is regulated by negatively charged surfaces. Factor XIIa is protected from C1INH by artificial negatively charged surfaces, but biologic surfaces such as polyphates potentiate C1INH inhibition of activated C1 s. In addition to being a plasma protein made in the liver, it is present in platelets and endothelial cells. The role of C1INH in these cells of the intravascular compartment is not completely known. How C1INH is regulated also is not completely known. Gamma interferon up-regulates C1INH hepatic mRNA and protein and patients treated with gamma interferon have higher levels of C1INH. All forms of hereditary angioedema (HAE) are due to reduced C1INH. Type 1 HAE is a true deficiency of C1INH and Type 2 HAE is an abnormal functioning C1INH. Type 3 HAE is C1INH deficiency due to its consumption from a constitutively activated form of factor XII. A consumptive form of acquired C1NH may occur due to anti-idiotype antibody with C1 activation and secondary C1INH consumption and angioedema. In acute attacks of HAE, prekallikrein (PK) is activated to plasma kallikrein that is in-part inhibited by complexes with alpha-2-macroglobulin. Both plasma PK and high molecular weight kininogen (HK) are consumed in acute attacks of HAE. The absence of C1INH is associated with cleavage plasma kallikrein cleavage of HK. Since cleaved HK is cleared in about 10 h, it becomes a reliable test for determination of activated states of plasma kallikrein resulting from C1INH deficiency. The final common pathway for angioedema in HAE is bradykinin delivery to tissues. Intravascular factor XII and PK each account for about 50% of the constitutive plasma level of bradykinin. Although therapies for HAE are directed towards many manifestations of the disorder, inhibitors of bradykinin receptor activation, plasma kallikein inhibition, suppression of plasma factor XII or PK, and C1INH replacement, no therapy is directed towards underlying mechanism for initiation of the disorder. In fact, the basic mechanism for initiation of plasma kallikrein activation in acute attacks of HAE still is as yet unknown. Most assume that it is associated with contact activation and factor XIIa formation with secondary plasma PK activation. Yet classic contact activation disorders such as surface activation on artificial medical surfaces or gram negative sepsis are conditions not associated with angioedema. Accordingly acute attacks of HAE are not associated with thrombin formation to any great extent and thrombosis. Thus other alternatives for the initiation of this disorder need consideration. Only until we fully understand the pathogenesis of common and variant forms of this disease will we be able to institute complete and definitive therapy for these deficiency states.

### I-2 Angioedema and idiopathic systemic capillary leak syndrome

#### Marco Cicardi

##### Department of Biomedical and Clinical Sciences Luigi Sacco, University of Milan, ASST Fatebenefratelli Sacco, Milan, Italy

###### **Correspondence:** Marco Cicardi (marco.cicardi@unimi.it)

*Allergy, Asthma & Clinical Immunology* 2017,** 13(Suppl 2)**:I-2

Edema is typical feature of inflammatory reactions and of changes in hydrostatic and oncotic forces that modify Starling equation. A third form of edema is due to primary modification of vessels’ permeability upon specific stimulus and not in response to other pathologic events. This condition realizes locally in angioedema and systemically in idiopathic systemic capillary leak syndrome (ISCLS). Both conditions can be spontaneously recurring, both suddenly revert to normal without healing period, both spare pulmonary circulation. Pathologic consequences exclusively depend on fluid extravasation: local tumor in angioedema, hypovolemic shock in ISCLS. Identified mechanisms of angioedema comprehend mast cells’ degranulation, kallikrein dependent bradykinin generation, kallikrein independent bradykinin accumulation. The etiopathogenesis of ISCLS is unexplained. Angioedema diagnosis first rely on the clinical features of edema that can localize to skin, gastrointestinal and upper airway mucosa. Biochemical (C1 inhibitor and C4 measurements) and genetic (F12 genotyping) testing refine diagnosis. ISCLS is diagnosed on clinical criteria of recurrent hypovolemic shock with profound hypoproteinemia in absence of an obvious cause. Most patients with ISCLS present a monoclonal (IgG) gammopathy of uncertain significance. Both conditions can be lethal and attacks highly distressing and disabling. Upon resolution, angioedema leaves no permanent damages. Long-term complications of acute episodes in ISCLS are peripheral nerve palsy, chronic renal failure and stroke. Corticosteroids and epinephrine are indicated for acute mast cells dependent angioedema, while recurrences are prevented by high dose antihistamine. Initial reports suggest omalizumab to be effective in preventing antihistamine-resistant mast cells dependent angioedema. Several treatments replacing C1-INH, antagonizing kallikrein and blocking bradykinin receptor 2 are effective for on demand and/or prophylactic treatment of angioedema due to C1-INH deficiency. Other non-mast cells dependent angioedema are still without approved therapy. Scattered reports suggest potential efficacy of tranexamic acid in prophylaxis, efficacy of on demand bradykinin/kallikrein targeted drugs is still controversial.

### I-3 Angioedema-histamine or bradykinin?

#### Avner Reshef

##### Angioedema Center, The Sheba Medical Center, Tel-Hashomer, 5265601, Israel

###### **Correspondence:** Avner Reshef (avner.reshef@sheba.health.gov.il)

*Allergy, Asthma & Clinical Immunology* 2017,** 13(Suppl 2)**:I-3

The underlying physiology of angioedema (AE) involves swelling of the skin or submucosal surfaces, caused by leakage of plasma into the tissues, owing to increased vascular endothelial hyperpermeability. This is the final common pathway of all AE syndromes, which should be differentiated from edema formation due to increased hydrostatic venous pressure, reduced oncotic pressure or decreased lymphatic drainage.

At the vascular level, endothelial integrity is maintained by adherens junctions, consisting of homotypic dimers of *VE*-*cadherin* bridges that allow normal paracellular transport of solutes. These bridges are stabilized and protected by adjacent *catenin* molecules. Two chemical mediators: *histamine* (H) *and bradykinin* (BK) are playing a central role in most AE syndromes. Both can induce phosphorylation of adherens junctions, and *catenin*-mediated coupling of *VE*-*cadherins* with cytoskeletal *actomyosin*, resulting in opening of cell–cell junctions. This process was shown to be activated in hereditary AE by signalling through BK receptors (BKRB 1&2).

AE syndromes are clinically heterogeneous and therefore present a considerable diagnostic challenge. *Urticaria* is characterized by the appearance of wheals which involves superficial cutaneous layers, surrounded by a reflex erythema and associated with intense pruritus. *Angioedema* (hereditary and acquired) involves the deep dermis, subcutaneous tissue and mucous membranes, and is associated with circumscribed swelling and dull pain. Both conditions are chronic/relapsing, with attacks and remissions. Interestingly, attacks mediated by BK are often preceded by premonitory signs and symptoms (prodromes), but not H-mediated AE.

Despite the apparent dichotomy between these two entities, there is recent evidence for a possible interaction between H-mediated AE and BK-mediated AE syndromes. Rarely, C1-INH deficient patients may present with prodromal pruritic rash that may evolve into a typical edematous HAE attack, and there are few reports of urticaria in HAE patients. Conversely, BK is involved in allergic reactions. In experimental shock/edema induced by insect stings increased *FXIIa*-*C1*-*INH* and *kallikrein*-*C1*-*INH* complexes were demonstrated, and total plasma *high*-*molecular kininogens* (HK) were cleaved within minutes. Cleavage of HK plasma pools was also demonstrated in patients with food-induced anaphylaxis and mast cell (MC)-derived *tryptase* can induce vascular permeability by releasing BK. Additionally, IgE-mediated anaphylaxis was blocked in FXII-deficient, BKB2R knock-out and HK/PreKallikrein-deficient mice. Recently, ablation of FXII, BKRB2 receptors and depletion of C1-INH protected mice from activated MC *heparin* induced vascular leakage. Additionally, negatively charged *heparin* from activated MC was shown to induce excessive vascular leakage in C1-INH deficient mice, and to trigger FXII auto-activation, while pretreatment with BKB2R antagonist (icatibant) inhibit vascular hyperpermeability and hypotension.

In summary, both H and BK are attributable to vascular endothelial hyperpermeability in AE syndromes. Recent data indicate that there might be a ‘cross-talk’ between MC-mediated ‘allergic’ events, and activation of BK-generating contact cascade.

### I-4 Emergent nations of Eastern Europe and hereditary angioedema. A case report

#### Dumitru Moldovan

##### Romanian Network for Hereditary Angioedema, MediQuest Medical Center, Tirgu Mures, Romania

###### **Correspondence:** Dumitru Moldovan (moldovan.dumitru@gmail.com)

*Allergy, Asthma & Clinical Immunology* 2017,** 13(Suppl 2)**:I-4

After the Second World War Eastern Europe was left to the Communist USSR zone, which resulted in a widening gap between East and West. It was only in 1989 when a new era began in this part of the world. Romania (as well as other former communist countries) was going to join EU and NATO. The West–East exchange programs and freedom of travel greatly improved the condition of Romanian physicians. It was in 2005 when I was invited the first time to join a C1-inhibitor workshop in Budapest. Despite the big post-communist handicaps of the Romanian health system I decided to dedicate the next few years to developing an HAE comprehensive program in my country. Surrounded by a handful of enthusiastic colleagues, I started to look for sporadic cases in some personal databases of colleagues from immunology labs, and also asked for the addresses of those with low C1 inhibitor results. Since 2005 we have organized workshops related to the annual conferences of the Romanian Society of Allergology and Clinical Immunology and later meetings centered on HAE only. From the beginning the support of HAE experts from Europe and later from Israel and USA was significant. In these past 12 years, we have made presentations at the conferences of the national meetings of paediatrics, dermatology, internal medicine, emergency medicine and published papers in order to increase the awareness about this life-threatening disease. In the past 10 years, we have participated in 12 multicentre clinical trials. In 2006 we founded the Romanian Network for Hereditary Angioedema, a professional non-profit organization who allowed us to have our own website, to develop a Romanian HAE guideline and to put together a national registry of HAE patients, which currently includes 99 patients. With our support, the Association of the Romanian HAE patients was founded in 2012. Until 2013 no modern treatments were available. Recombinant C1-inhibitor and a C1-inhibitor concentrate were offered in ED in a small amount from 2015 and it was promised that the B2 receptor antagonist (icatibant) will be reimbursed through a national program that will start soon. Hopefully, the quality of life of these patients will improve considerably. There is still a lot to do to raise the awareness and expand the education of both physicians and patients about this disorder; however, our aim is above all to address the challenge of finding an estimated 250 patients that are missing from our database.

### I-5 Neutrophils and neutrophil signaling in inflammation

#### Attila Mócsai

##### Semmelweis University, School of Medicine, Budapest, Hungary

###### **Correspondence:** Attila Mócsai (mocsai.attila@med.semmelweis-univ.hu)

*Allergy, Asthma & Clinical Immunology* 2017,** 13(Suppl 2)**:I-5

Neutrophils are double-edged swords playing a critical role in innate immune defense but also contributing to tissue damage during excessive inflammation. Their exact role in those processes and the molecular details of their function are still poorly understood. We and others have recently shown that neutrophils are critically involved in various in vivo models of innate immunity and inflammation in experimental mice. Neutrophils are also able to release a number of pro-inflammatory mediators including chemokines, cytokines and lipid mediators such as LTB4. Many of the mediators released by neutrophils promote the recruitment or activation of other neutrophils, therefore establishing positive feedback amplification loops during neutrophil-mediated inflammation. Immunoreceptor-induced neutrophil responses required receptor-proximal Src-family kinases, Syk and PLCγ2 which were also critical for neutrophil-mediated in vivo inflammatory reactions. Those molecules were involved in the release of proinflammatory mediators from neutrophils but not in the intrinsic ability of the cells to migrate to the site of inflammation. Interestingly, the downstream gene expression regulator CARD9 was required for the release of chemokines and cytokines but not for LTB4 release or other classical neutrophil responses, providing a unique opportunity to test the in vivo role of neutrophil gene expression changes and neutrophil-derived chemokines/cytokines. Neutrophil-specific deletion of CARD9 attenuated various different in vivo inflammation models, providing the first direct evidence for the role of neutrophil gene expression changes during an in vivo inflammatory reaction.

## Round table discussion

### RT-1 Molecular analysis of SERPING1 gene: to whom, when and how?

#### Margarita López-Trascasa, Alberto López Lera

##### Unidad de Inmunología, Hospital Universitario La Paz, IdiPAZ, CIBERER U754, Madrid, Spain

###### **Correspondence:** Margarita López-Trascasa (mltrascasa@salud.madrid.org)

*Allergy, Asthma & Clinical Immunology* 2017,** 13(Suppl 2)**:RT-1

Hereditary angioedema due to C1 inhibitor deficiency (HAE-C1INH) can be diagnosed by two specific complement parameters: C4 levels and C1 inhibitor function. In patients with a family history, these measurements are generally sufficient to establish or discard the presence of the disease.

As a general rule, genetic studies represent an additional tool for reaching a conclusive diagnosis; especially in three situations which are relatively common in HAE-C1INH diagnosis: de novo cases, relatives of affected patients without clear evidences of HAE symptoms and those samples with inconclusive results following complement testing.

Nowadays, mutational studies are easier and cheaper. In addition to traditional Sanger sequencing, the development of integrated analysis platforms including RT-PCR (splicing mutations), RT-qPCR (RNA expression studies), MLPA and XL-PCR (large insertions/deletions) allows a complete, rapid and efficient identification of most of the disease-causing mutations. In our lab, these techniques are paralleled by the study of complement levels and function and integrated as a trademark (Complementest^®^).

We systematically perform a full HAE-C1INH study to every new patient referred due to suspicion of the disease. Upon compatible C4 levels and C1Inh function, we first attempt to characterize the mutation in the *propositus* and then to establish segregation of the mutation in their first degree relatives. In the case of newborns with an affected parent, a simple Sanger sequencing of the altered exon is enough for a straightforward diagnosis of point mutations.

When HAE is not associated with C1 inhibitor dysfunction or low C4 levels, HAE with normal C1 inhibitor (HAE-nC1INH) should be considered. In such cases, mutations in the ninth exon of coagulation F12 are present in less than 40% of cases, so that the clinical presentation, lack of response to allergic treatments and familial history should all be considered in support of a HAE diagnosis.

In addition to that, and although acquired angioedema with conserved C1q levels is very unusual and presents different features, genetic studies can unambiguously discriminate hereditary and acquired situations.

### RT-2 Pharmacogenetics of angiotensin-converting enzyme (ACE) inhibitor-associated angioedema

#### Nancy J. Brown

##### Department of Medicine, Vanderbilt University Medical Center, Nashville, TN, USA

###### **Correspondence:** Nancy J. Brown (nancy.j.brown@Vanderbilt.Edu)

*Allergy, Asthma & Clinical Immunology* 2017,** 13(Suppl 2)**:RT-2

Whereas hereditary angioedema results from increased production of kinins, angiotensin-converting enzyme (ACE) inhibitor-associated angioedema results from decreased degradation of bradykinin and other vasoactive substrates of ACE such as substance P. Candidate gene studies have examined the association between functional variants in genes encoding for enzymes involved in the degradation of bradykinin and other peptides and ACE inhibitor-associated angioedema. For example, aminopeptidase P (APP) degrades bradykinin when ACE is inhibited. A single nucleotide polymorphism (SNP) in the gene encoding membrane APP (*XPNPEP2*, −2399C>A or rs3788853) has been associated with decreased plasma APP activity and angioedema in families with anaphylactoid reactions during hemodialysis and/or angioedema during ACE inhibitor use [1]. Membrane APP is encoded for by an X-linked gene, however, and ACE inhibitor-associated angioedema is more common in women than in men. We have found that the −2399A allele is associated with decreased plasma APP activity in men and women, but that the variant allele is associated with ACE inhibitor-associated angioedema only in men [2]. Neprilysin also degrades bradykinin and substance P. Our group has observed an association between a polymorphism in the gene encoding neprilysin (*MME*) and ACE inhibitor-associated angioedema of African American ancestry in a case-control study in Tennessee as well as in the Ongoing Telmisartan Alone and in Combination with Ramipril Global Endpoint Trial (ONTARGET) study [3]. In a genome-wide association study in 175 individuals with ACE inhibitor-associated angioedema and 489 ACE inhibitor-exposed controls without angioedema from Nashville (Tennessee) and Marshfield (Wisconsin), we found no associations of genome-wide significance [3]. Of the SNPs that were associated modestly with ACE inhibitor-associated angioedema in Nashville/Marshfield analysis, two were also significantly associated with ACE inhibitor-associated angioedema (rs500766 and rs2724635) in ONTARGET cases versus controls. Rs500766 is a polymorphism in the gene encoding for protein kinase C θ (*PRKCQ*). In both the Nashville/Marshfield sample and in ONTARGET, the T allele was significantly associated with a reduced risk of ACE inhibitor-associated angioedema. Rs2724635 is a polymorphism in ETS variant gene 6 (*ETV6*), also known as TEL (or translocation ets leukemia), and the G allele was associated with an increased risk of ACE inhibitor-associated angioedema in both African Americans in the Nashville/Marshfield sample and in the ONTARGET sample. Both of these genes are involved in immune regulation and their association with ACE inhibitor-associated angioedema is intriguing as the risk of ACE inhibitor-associated angioedema is increased in patients with seasonal allergies and with immunosuppressant use [4, 5].

**References**

1. Duan QL, Nikpoor B, Dube MP, Molinaro G, Meijer IA, Dion P, et al. A variant in xpnpep2 is associated with angioedema induced by angiotensin i-converting enzyme inhibitors. Am J Hum Genet. 2005;77:617–26.

2. Woodard-Grice AV, Lucisano AC, Byrd JB, Stone ER, Simmons WH, Brown NJ. Sex-dependent and race-dependent association of xpnpep2 c-2399a polymorphism with angiotensin-converting enzyme inhibitor-associated angioedema. Pharmacogenet Genom. 2010;20:532–6.

3. Pare G, Kubo M, Byrd JB, McCarty CA, Woodard-Grice A, Teo KK, et al. Genetic variants associated with angiotensin-converting enzyme inhibitor-associated angioedema. Pharmacogenet Genom. 2013;23:470–8.

4. Byrd JB, Woodard-Grice A, Stone E, Lucisano A, Schaefer H, Yu C, et al. Association of angiotensin-converting enzyme inhibitor-associated angioedema with transplant and immunosuppressant use. Allergy. 2010;65:1381–7.

5. Straka B, Nian H, Sloan C, Byrd JB, Woodard-Grice A, Yu C, et al. Pollen count and presentation of angiotensin-converting enzyme inhibitor-associated angioedema. J Allergy Clin Immunol Pract. 2013;1:468–73 e461–464.

### RT-3 Genotyping C1-INH deficiency patients: methods, pitfalls and biases

#### Anastasios E. Germenis

##### Department of Immunology and Histocompatibility, School of Health Sciences, Faculty of Medicine, University of Thessaly, Larissa, Greece

###### **Correspondence:** Anastasios E. Germenis (agermen@med.uth.gr)

*Allergy, Asthma & Clinical Immunology* 2017,** 13(Suppl 2)**:RT-3

With every passing day, genotyping of subjects who may suffer from hereditary angioedema becomes more indispensable in the clinical practice. However, the pronounced allelic heterogeneity of *SERPING1* gene as well as the fact that approximately 20–25% of all unrelated patients with hereditary angioedema due to C1-inhibitor deficiency (C1-INH-HAE) represent sporadic cases carrying de novo *SERPING1* mutations with the same mutational spectrum as the familial ones, makes *SERPING1* a prime example of mutagenic lability. Over 450 different *SERPING1* mutations related with C1-INH-HAE have been detected up to now, scattered over all exons and exon/intron boundaries of the gene. The majority of C1-INH-HAE-related *SERPING1* mutations are missense mutations (34%) followed by frameshift alterations and small indels (31%), large gene rearrangements (17%), splice-site defects (10%), nonsense mutations (7%), and regulatory mutations (1%). Currently, the molecular analysis of *SERPING1* initiates with the prioritized amplification of all exons and the exon/intron boundaries by PCR and the detection of mutations by direct sequencing. If no mutation were found, further analysis for the identification of large gene rearrangements is taking place, usually performed by two different techniques, namely the long-range PCR and the multiplex ligation-dependent probe amplification (MLPA). This cumbersome and time-consuming approach is fraught with pitfalls as it is highly dependent on the users’ experience and knowledge. Sometimes, for example, a not yet reported missense mutation could be considered as the causal genetic defect because its detection during the early steps of the analysis has prevented the discovery of an indel leading to premature truncation of protein synthesis. Moreover, recently recognized damaging intronic alterations located in the exon/intron boundaries—and not only—may escape the conventional approach, as they require the design of specific primers. On the other hand, however, modern genotyping methods, like the next-generation sequencing (NGS), present many advantages towards a more meticulous *SERPING1* analysis. Therefore, the increasingly frequent use of genotyping angioedema patients in the clinical practice and research imposes the standardization of the methods and workflows in use for *SERPING1* analysis. Special attention should be paid in regard with the interpretation of the detected sequence variants according to the recently developed recommendations.

## Oral presentations

### O-1 Bradykinin displays a biased agonism profile in B2 receptor mutants identified in patients with hereditary angioedema

#### Rafael Filippelli-Silva^1,2^, Diego A. Duarte^3,4^, Renan P. Martin^1^, Camila L. Veronez^1,2^, Michel Bouvier^4^, Michael Bader^2^, Claudio M. Costa-Neto^3^, João B. Pesquero^1^

##### ^1^Department of Biophysics, UNIFESP, São Paulo, São Paulo, 04039-032, Brazil; ^2^Department of Molecular Biology of Peptide Hormones, Max-Delbrück-Center for Molecular Medicine, Berlin, Berlin, 13125, Germany; ^3^Department of Biochemistry and Immunology, FMRP-USP, Ribeirão Preto, São Paulo, 14049-900, Brazil; ^4^Department of Biochemistry and Molecular Medicine and Institute for Research in Immunology and Cancer, Université de Montréal, Montreal, QC, 6128, Canada

###### **Correspondence:** Rafael Filippelli-Silva (rafaelfilippelli@gmail.com)

*Allergy, Asthma & Clinical Immunology* 2017,** 13(Suppl 2)**:O-1

**Background:** Bradykinin (BK) plays an important role on blood pressure control, nociception, inflammation and has also been associated with the pathophysiology of Hereditary Angioedema (HAE). The main clinical aspects of HAE are mediated by BK, which exerts its actions by B_2_ receptor’s (B2R) activation, a member of the G protein coupled receptor (GPCR) family. The aim of the present work was to evaluate whether two B2R mutations found in HAE patients could alter the BK signal transduction pharmacological profile, therefore accounting relevant consequences for the disease’s pathophysiology.

**Materials and methods:** We used G-protein and β-arrestins biosensors constructs to perform BRET analyses and assess functional profiles of mutated B2R. All experiments were carried out in HEK293T cells transiently transfected either with the human wild-type or mutants B2R. Competition binding assays using radiolabeled BK (^3^H-BK) were performed in order to estimate BK affinity for mutant receptors. BRET assays to assess G-protein activation and β-arrestin1 and 2 recruitment were performed using an adapted method from that described by Quoyer et al. [1].

**Results:** Competition binding assays show that BK maintains a similar high affinity to the mutant receptors #1 and #2 (0.35 and 0.46 nM, respectively), when compared to wild-type receptor (0.39 nM). In functional analyses, BK triggered G-protein coupling with similar potencies in the wild-type and mutant receptors. On the other hand, BK could not trigger β-arrestin recruitment when binding to mutant receptors, whereas binding to wild-type receptor a clear dose-response curve for β-arrestin 1 and 2 recruitment was observed (potency of 32 and 16 nM, respectively).

**Conclusions:** These findings provide strong evidence for a G protein biased agonism profile [2] of endogenous BK when activating B2R mutants identified in patients having HAE. We believe the present data contributes to unveiling the molecular mechanisms involved in HAE and therefore sheds light to future approaches for treatment of this disease.

**Acknowledgements:** This study was supported by grants of FAPESP, CNPq, and CAPES.

**References**

1. Quoyer J, Janz JM, Luo J, Ren Y, Armando S, Lukashova V, Benovic JL, Carlson KE, Hunt SW 3rd, Bouvier M. Pepducin targeting the C-X-C chemokine receptor type 4 acts as a biased agonist favoring activation of the inhibitory G protein. Proc Natl Acad Sci USA. 2013;110(52):E5088-97.

2. Costa-Neto CM, Parreiras-E-Silva LT, Bouvier M. A pluridimensional view of biased agonism. Mol Pharmacol. 2016;90(5):587–95.

### O-2 Design of fusion proteins that bind B1 and B2 receptors for bradykinin: progress toward diagnostic tools

#### Xavier Charest-Morin, François Marceau

##### CHU de Québec-Université Laval, Quebec City, QC, G1V 4G2, Canada

###### **Correspondence:** Xavier Charest-Morin (xavier.charest-morin.1@ulaval.ca)

*Allergy, Asthma & Clinical Immunology* 2017,** 13(Suppl 2)**:O-2

**Background:** Few anti-bradykinin (BK) B_1_ and B_2_ receptor (B_1_R, B_2_R) antibodies are validated, especially for work in intact cells. Since kinins bind their receptor through their C-terminal region and that their N-terminal region is close to the extracellular fluid, fusion protein of the type *H*_*2*_ *N*-*Protein*-*Spacer*-*Agonist*-*COOH* were generated and evaluated for the detection of each receptor type. We recently reported the fusion of the enhanced green fluorescent protein (EGFP) to the C-terminal of each *spacer*-*agonist* module (the amphibian sequence maximakinin (MK) for B_2_Rs, (Asn-Gly)_n_-Lys-des-Arg^9^-BK for B_1_Rs, n = 5 or 15), yielded bright imaging tools of nanomolar potency that do not bind to peptidases. The specificity and design of ligand fusion proteins was further explored.

**Methods:** Using various molecular biology techniques, vectors encoding for different fusion proteins were generated. These vectors were then expressed in producers cells which were ultimately lysed to extract the fusion proteins. The cell lysate was cleared of cellular debris and used on recipient cells.

**Results:** Further studies showed that MK (the 19-mer DLPKINRKGPRPPGFSPFR possessing the BK sequence at its C-terminus) is largely a species-specific *spacer*-*agonist* module that does not bind well to human B_2_Rs, but supports the detection of rat and rabbit B_2_Rs. Moreover, the B_2_R putative agonist (Asn-Gly)_5_-BK has very little affinity for either human or rat B_2_Rs, proving that each *spacer*-*agonist* module has a critical structure-activity relationship for each receptor subtype. The fusion of the engineered soybean peroxidase APEX2 to appropriate *spacer*-*agonist* modules led to BK receptors ligands compatible with widely available luminescent or imaging peroxidase co-substrates and supported the detection of physiological levels of BK receptors. APEX2-(Asn-Gly)_n_-Lys-des-Arg^9^-BK constructions (n = 15, 30, 45 or 60) all bind human B_1_Rs. APEX2-(Asn-Gly)_15_-MK was developed as a B_2_R ligand and supports the detection of rat B_2_Rs in a specific manner. The fusion protein human serum albumin-MK did not bind B_2_Rs, showing that some proteins are incompatible with this strategy, possibly due to steric hindrance between the fused protein and the receptor. Other functional proteins, such as β-galactosidase and the Fc region of IgG, are being investigated for the generation of BK receptor ligands.

**Conclusions:** The fusion proteins of the type *H*_*2*_ *N*-*Protein*-*Spacer*-*Agonist*-*COOH* represent the basis for the generation of better tools for the detection of BK receptors in intact cells. Such probes could have diagnostic applications and support physiological and pathological investigations especially in HAE.

Supported by Canadian Institutes of Health Research and Fonds de Recherche du Québec-Santé.

### O-3 Comparing pathways of kinin formation in human whole blood: generating endogenous agonists of the bradykinin B_2_ and B_1_ receptors

#### François Marceau^1^, Georges-É. Rivard^2^, Arnaud Bonnefoy^2^, Éric Wagner^1^, Xavier Charest-Morin^1^

##### ^1^CHU de Québec-Université Laval, Quebec City, QC, Canada G1V 4G2, Canada; ^2^CHU Ste-Justine, Montreal, QC, Canada H3T 1C5, Canada

###### **Correspondence:** François Marceau (francois.marceau@crchudequebec.ulaval.ca)

*Allergy, Asthma & Clinical Immunology* 2017,** 13(Suppl 2)**:O-3

**Background:** Multiple pathways have been proposed to generate bradykinin (BK)-related peptides from blood, including some dependent of secretory products from neutrophils or platelets. We applied various forms of activation to fresh blood obtained from healthy subjects to compare kinin formation with reference to mechanisms, kinin concentrations, time course and validation of biological activity at both BK receptor subtypes.

**Materials and methods:** An enzyme immunoassay for BK (Phoenix Pharmaceuticals) was applied to reconstituted extracts of citrated blood incubated at 37 °C under gentle agitation for 0–2 h in the presence of activators and/or inhibitory drugs. Validation of the presence of biologically active kinin is necessary because the immunoassay is reactive with a limited BK C-terminal sequence only. The applied bioassays were the contractility of the human umbilical vein and signaling (c-Fos accumulation) in HEK 293a cells that express recombinant human B_2_ or B_1_ receptors (B_2_R, B_1_R).

**Results:** The ACE inhibitor enalaprilat did not induce immunoreactive BK (iBK) formation in human blood, but considerably potentiated the effect of the stimuli that were effective. Rapidly (5 min) and intensely (>200 ng/ml iBK) acting stimulants were recombinant tissue kallikrein (KLK-1) and Kontact-APTT, a particulate material that recruits the contact system. Corresponding extracts contracted the umbilical vein via the BK B_2_R and stimulated recombinant B_2_Rs; B_1_Rs were also stimulated by KLK-1-treated blood extracts. Recombinant tissue plasminogen activator was a slowly (≥1 h) acting stimulus inhibited by corn trypsin inhibitor and PKSI-527. Stimulating neutrophil secretion using cytochalasin B + f-Met-Leu-Phe or NETosis with interleukin-8 did not generate iBK. ADP (≥50 μM) was also inactive in this respect, even if the blood was submitted to shear stress in an aggregometer.

**Conclusions:** Comparing the proposed kinin generation pathways in whole normal blood, where endogenous inhibitors abound, put some of them in perspective, e.g. lack of effect of secreted neutrophil PR3 and of platelet activation, indirect and slow effect of fibrinolysis via the contact system. Both KLK-1 and the contact system ultimately recruit kinin receptors. Whether atypical kinin formation/potentiation participate to hereditary or acquired/sporadic angioedema attacks is plausible.

**Acknowledgements:** Supported by an Investigator-Initiated Research Grant from Shire International GmbH. We thank Ms. J. Bouthillier for technical help.

### O-4 A novel potential triggering factor of HAE attacks: complement MASP-1 increases endothelial cell permeability

#### Márta L. Debreczeni, Zsuzsanna Németh, Erika Kajdácsi, Endre Schwaner, László Cervenak

##### 3rd Department of Internal Medicine, Semmelweis University, Budapest, Hungary

###### **Correspondence:** Márta L. Debreczeni (martidebreczeni@gmail.com)

*Allergy, Asthma & Clinical Immunology* 2017,** 13(Suppl 2)**:O-4

**Background:** Hereditary angioedema resulting from the *deficiency* of *C1 inhibitor* (C1–INH–HAE) is characterized by increased endothelial permeability. Mannan-binding lectin-associated serine protease-1 (MASP-1)—one of the enzymes blocked by C1-inhibitor—is the key serine protease of complement lectin pathway. We previously showed that (1) MASP-1 can activate endothelial cells via protease activated receptor (PAR) cleavage and (2) endothelial cells become activated during C1–INH–HAE attacks. These findings led us to ask whether MASP-1 can increase endothelial permeability and thus take part in the triggering of edematous attacks. To answer this question it was necessary to develop a new and simple permeability test. Using human umbilical vein endothelial cell (HUVEC) culture as an in vitro model we also aimed to explore the mechanisms by which MASP-1 exerts its cellular effects.

**Results:** We have successfully optimized a new, simple, high-throughput and cheap method for permeability measurements, which is based on the reaction of biotinylated gelatin and a streptavidin-conjugated fluorescent dye.

In this test, MASP-1 could significantly and dose-dependently increase endothelial permeability 20 min after treatment. This effect was completely blocked by C1-inhibitor and a highly specific artificial MASP-1 inhibitor, SGMI-1. We demonstrated that the permeability increasing effect of MASP-1 is mediated mostly by PAR-1. We also found that MASP-1 induced endothelial permeability requires the activity of p38 mitogen activated protein kinase (p38 MAPK) and p160 Rho associated coiled-coil containing protein kinase (p160 ROCK). In fluorescence microscopy experiments we demonstrated that MASP-1 induces diphosphorylation of myosin light chains resulting in cytoskeletal actin rearrangement, which was also dependent on p160 ROCK activity.

Visualization of key molecules involved in cell adhesion showed that MASP–1 changes the molecular pattern of PECAM-1, VE-cadherin and ZO-1, and induces paracellular gap formation.

To analyze the long-term effects of MASP-1 on the gene expression profile of HUVECs we carried out an Agilent microarray. MASP-1 significantly changed the expression of 12 permeability-related genes; among others it down-regulated genes involved in cell junctions and actin cytoskeleton regulation, while it up-regulated B2 bradykinin receptor.

**Conclusion:** In our experiments MASP-1 was found to be a potent short-term permeability increasing agonist, but according to our microarray results it may also have a long-term effect on endothelial permeability. These findings raise the possibility that MASP-1 could play an important role in the pathomechanism of C1-INH-HAE, which makes SGMI-1 a potential target of future drug development.

**Supported by:** Hungarian Scientific Research Fund (OTKA K115623).

### O-5 Modeling the activation and inhibition by C1 inhibitor of lectin pathway proteases

#### Gábor Oroszlán, András Szilágyi, Ráhel Dani, Péter Závodszky, Péter Gál, József Dobó

##### Institute of Enzymology, Research Centre for Natural Sciences, Hungarian Academy of Sciences, Magyar tudósok krt. 2., 1117, Budapest, Hungary

###### **Correspondence:** József Dobó (dobo.jozsef@ttk.mta.hu)

*Allergy, Asthma & Clinical Immunology* 2017,** 13(Suppl 2)**:O-5

Proteases are generally synthesized as precursors, termed proenzymes or zymogens, and the fully active form is produced via limited proteolysis by another protease or autoactivation. The lectin pathway of the complement system is initiated by mannose binding lectin (MBL)-associated serine proteases, MASP-1, and MASP-2, which are known to be present as proenzymes. The third serine protease of the lectin pathway, MASP-3, was shown to be the major activator, and the exclusive “resting blood” activator of pro-factor D (pro-FD), producing factor D (FD), the initiator protease of the alternative pathway. Because only activated MASP-3 is capable to carry out this cleavage, it was presumed that a significant fraction of MASP-3 must be present in the active form in resting blood.

We demonstrated the presence of active MASP-3 in blood by a more direct technique. First MASPs were partially purified by affinity chromatography using immobilized MBL in the presence of inhibitors. Using this MASP pool only the zymogen form of MASP-1 was detected by Western blot, whereas MASP-3 was over 70% active. The active to zymogen ratio of MASP-3 showed little individual variation. It is enigmatic how MASP-3, which is not capable to autoactivate, is present mostly as an active enzyme, whereas MASP-1, which has a potent autoactivation capability, is predominantly proenzymic in resting blood.

In an attempt to explain this phenomenon we modeled the fluid-phase activation and subsequent inactivation by C1 inhibitor of lectin pathway proteases using available and newly determined kinetic constants. The model can explain extensive MASP-3 activation if we assume efficient intercomplex activation of zymogen MASP-3 by zymogen MASP-1, however the model is in good agreement with the fact that MASP-1 and -2 are predominantly proenzymic and part of them is present in the form inactive serpin-protease complexes.

Our approach can be useful to model protease—C1 inhibitor reactions in C1 inhibitor sufficient and deficient conditions in the future.

### O-6 Algorithm to distinguish histamine and bradykinin-induced angioedema in Emergency Department

#### Jacques Hébert^1^, Matthieu Vincent^2,3^, Jean-Nicolas Boursiquot^1^, Hugo Chapdeleine^4^, Marylin Desjardins^5^, Benoit Laramée^6^, Rémi Gagnon^1^, Nancy Payette^1^,Oleksandra Lepeshkina^1^

##### ^1^Centre hospitalier Universitaire de Québec, Université Laval, Quebec, Canada; ^2^CHU Ste-Justine, Université de Montréal, Montreal, Canada; ^3^Hôpital Charles Lemoyne, Greenfield Park, Canada; ^4^Centre Hospitalier Universitaire de Montréal, Université de Montréal, Montreal, Canada; ^5^Université McGill, Montreal, Canada; ^6^Clinique Médicale 504 Notre-Dame, Repentigny, Canada

###### **Correspondence:** Jacques Hébert (Hebert.j@videotron.ca)

*Allergy, Asthma & Clinical Immunology* 2017,** 13(Suppl 2)**:O-6

**Background:** Angioedema (AE) is characterized by localized swelling of subcutaneous tissues or mucosa of the upper respiratory or gastrointestinal tract. It accounts for as many as 80,000–112,000 Emergency Department (ED) visits annually with 18% resulting in hospitalisation [1]. There are two distinct subtypes of AE, caused by different pathological processes involving either mast-cell mediators, including histamine or bradykinin. The resulting AE often cannot be distinguished on clinical grounds and misdiagnosis may lead to inappropriate or delayed management which can be fatal particularly in patients with swelling of the upper respiratory tract.

In a real-life setting, most ED visits for AE, unless the patient is known to suffer from hereditary AE (HAE), will involve allergic or idiopathic AE, with or without concomitant urticaria or evidence of anaphylaxis. These forms of AE are typically mediated by histamine. Their management, usually familiar to ED staff, is made of adrenalin, antihistamines and corticosteroids [referred to as standard therapy (ST), Figure 1]. The key challenge in the ED, however, is recognizing and treating potential nonhistaminergic (bradykinin-mediated) AE. We suggest the distinction to be based on response to Standard Therapy.

**Results:** A satisfactory response to ST will support the diagnosis of histamine-induced AE and the patient will eventually be evaluated accordingly. In the case of non-optimal response, when inadequate dosing of antihistamines or potential beta-blockade have been addressed, bradykinin-mediated AE must be considered. Unlike histamine-mediated AE, bradykinin-mediated AE is not associated with urticaria, does not respond to antihistamines or corticosteroids, and is poorly responsive to adrenalin. It tends to be more severe, longer lasting, and much more likely to involve concurrent abdominal symptoms than its counterpart. Angiotensin-converting enzyme (ACE) inhibitor-associated angioedema represent the first cause of bradykinin-induced AE. ACE inhibitors then cause local elevations in plasma bradykinin because of inhibition of its normal breakdown. HAE is associated with increases in bradykinin following some trigger because of a reduced C1 esterase inhibitor (C1-INH) activity while the acquired form is found with lymphoproliferative diseases or autoimmune disorders as a result of the production of neutralizing autoantibodies against C1-INH or increased C1-INH consumption. Bradykinin-targeted first-line therapy [2] includes intravenous infusion of plasma-derived C1-INH (Berinert, 20 U/kg or Cinryze 1000 U) or recombinant C1-INH (Ruconest, 50–100 U/kg) or subcutaneous injection (30 mg) of icatibant, a bradykinin inhibitor (Firazyr) or ecallantide (Kalbitor), a highly specific plasma kallikrein inhibitor. Solvent detergent-treated fresh-frozen plasma (2 U) may be used as a second-line agent only in the absence of first-line agents. The selection of the therapeutic agent in individual patient management plans and the threshold for initiating treatment will be influenced by patient preferences and local policy.

**Conclusions:** This consensus algorithm focuses on the presentation of isolated AE disorders to the ED. It should permit a rapid identification of bradykinin-induced AE for specific therapy.

**References**

1. Joseph J. Moellman, Jonathan A. Bernstein, Christopher Lindsell. A consensus parameter for the evaluation and management of angioedema in the Emergency Department. Acad Emerg Med. 2014;21:469–84.

2. Iris M. Otani, Sandra C. Christiansen, Paula Busse, Carlos A. Camargo, Jr., Bruce L. Zuraw, Marc A. Riedl, and Aleena Banerji. Emergency Department management of hereditary angioedema attacks: patient perspectives. J Allergy Clin Immunol Pract. 2017;5:128–34.

### O-7 Bradykinin angioedema solution for biological diagnostic

#### Delphine Charignon^1,2^, Arije Ghannam^1,2^, Denise Ponard^3^, Christian Drouet^2,4^

##### ^1^KininX SAS, 38000, Grenoble, France; ^2^GREPI UE7408, Université Grenoble Alpes, Grenoble, France; ^3^CHU Grenoble, Grenoble, France; ^4^Centre de Référence des Angioedèmes (CREAK), Grenoble, France

###### **Correspondence:** Delphine Charignon (delphine.charignon@kininx.com)

*Allergy, Asthma & Clinical Immunology* 2017,** 13(Suppl 2)**:O-7

**Background:** Laboratory assays for AO diagnosis target complement while AO is subsequent to contact phase activation and bradykinin (BK) accumulation. The aim of this study was to compare diagnostic performances between methods targeting complement and contact phase.

**Materials and methods:** Diagnostic of hereditary angioedema with C1 inhibitor (C1Inh) deficiency (C1Inh-HAE) of patients was performed according to recommendations (1) and confirmed by genetic analysis of *SERPING1* gene. Patients taking tranexamic acid or danazol were excluded. Patients and blood donors provided informed consent to participate in the investigation.

Antigenic C4 was quantified by nephelometry. C1Inh function was measured by chromogenic assays targeting C1s using commercial kits (Technochrom, Technoclone GmbH or Berichrom, Siemens), by an in-house method (2) or by a new method targeting kallikrein (KK) (3). Spontaneous amidase activity was performed as described (4).

Diagnostic performance values and statistical analysis were performed using Xlstat^®^ software, and Mann–Whitney U tests, with *P* < 0.05 as statistically significant.

**Results:** Samples (n = 519) were assayed for C1Inh function; assay systems targeting C1s protease or a new assay targeting KK (3) were compared. Fifty eight samples displayed one discordance between methods. All methods presented with a very good specificity, while the sensitivities were different, mainly for intermediate or transient C1Inh deficiency. The in-house method presented a higher sensitivity than commercials kits, while its sensitivity was lower than the assay targeting KK.

A comparison between C4 and spontaneous amidase activity as companion test in C1Inh-HAE is described for 99 healthy subjects and 185 patients. Antigenic C4 and spontaneous amidase activity assays showed good specificity and high positive predictive value for C1Inh-HAE diagnosis. Nevertheless, spontaneous amidase activity displayed higher sensitivity and negative predictive values.

**Conclusion:** Assays targeting contact phase (C1Inh using KK or spontaneous amidase activity) displayed higher sensitivity than assay targeting complement (C1Inh using C1s or antigenic C4), in line with its pertinence for AO physiopathology. The higher sensitivity is advantageous for angioedema screening and biological diagnosis.

**References**

1. Cicardi M, Aberer W, Banerji A, Bas M, Bernstein JA, Bork K, et al. Classification, diagnosis, and approach to treatment for angioedema: consensus report from the Hereditary Angioedema International Working Group. Allergy. 2014;69:602–16.

2. Drouet C, Alibeu C, Ponard D, Arlaud GJ, Colomb MG. A sensitive method to assay blood complement C1-inhibitor activity. Clin Chim Acta. 1988;174:121–30.

3. Ghannam A, Sellier P, Defendi F, Favier B, Charignon D, López-Lera A, et al. C1 inhibitor function using contact-phase proteases as target: evaluation of an innovative assay. Allergy. 2015;70:1103–11.

4. Defendi F, Charignon D, Ghannam A, Baroso R, Csopaki F, Allegret-Cadet M, et al. Enzymatic assays for the diagnosis of bradykinin-dependent angioedema. PLoS ONE. 2013;8:e70140.

### O-8 Assay for functional C1 inhibitor deficiency: an ELISA that improves upon the chromogenic method

#### Kusumam Joseph^1,3^, Baby G. Tholanikunnel^1^, Daniel J. Sexton^2^, Allen P. Kaplan^1^

##### ^1^Department of Biochemistry and Molecular Biology, Medical University of South Carolina, Charleston, SC, 29425, USA; ^2^Shire Pharmaceuticals, Lexington, MA, 02421, USA; ^3^*Present Address* Shire Pharmaceuticals, Lexington, MA, USA

###### **Correspondence:** Kusumam Joseph (josephkusum@gmail.com)

*Allergy, Asthma & Clinical Immunology* 2017,** 13(Suppl 2)**:O-8

**Background:** The current diagnostic assay for functional C1 inhibitor (C1INH) measurement employs inhibition of activated C1s of the complement cascade by C1INH, utilizing either a chromogenic assay or a complex ELISA method. Since both the methods have limitations, there is a need to develop a more specific, sensitive and reliable method.

**Results:** We have previously reported that C1INH deficiency can be determined by ELISA methodology based on the concept that C1INH inhibits plasma kallikrein as well as activated factor XII. Employing either enzyme as ligand, we demonstrated superiority to the commercial ELISA method that is based on inhibition of activated C1. Our method is more accurate and sensitive. Although the commercial chromogenic assay is sensitive and specific than the commercial ELISA, a direct comparison between chromogenic and our ELISA was not done. We report results of a direct comparison of the chromogenic assay that the commercial laboratories use with our ELISA method employing either factor XIIa or kallikrein as the C1 inhibitor ligand. We assayed 30 normal controls and 57 patients with type I/II HAE. The values for normal controls by chromogenic assay was 146 ± 25; by factor XIIa ELISA was 148 ± 36; and by kallikrein ELISA was 129 ± 37 whereas the values for type I/II HAE were 37 ± 22 (chromogenic); 23 ± 12 (factor XIIa ELISA) and 19 ± 11 (kallikrein ELISA). The p value for the chromogenic assay comparing normal plasma to HAE plasma was <0.0001. The p value for the factor XIIa ELISA (or Kallikrein ELISA) comparing normal plasma to HAE plasma was also <0.0001. A comparison of HAE patients done by the chromogenic assay vs. our factor XIIa ELISA (or kallikrein ELISA) also had a p value of <0.0001.

**Conclusion:** The chromogenic method had greater uncertainty; one of the HAE plasma was misdiagnosed and 7 were considered equivocal. The factor XIIa ELISA (or kallikrein ELISA) method is rapid, easily scaled up for large number of samples and appears superior to the chromogenic method.

Funded by Dyax Corp., Burlington, MA, USA and Shire Human Genetics Therapies, Lexington MA, USA.

### O-9 Emerging role for phospholipase enzymes in hereditary angioedema with C1-INH deficiency

#### Stefania Loffredo^1^, Maria Bova^1^, Anne Lise Ferrara^1^, Angelica Petraroli^1^, Chiara Suffritti^2^, Nòra Veszeli^3^, Andrea Zanichelli^2^, Henriette Farkas^3^, Marco Cicardi^2^ and Gianni Marone^1,4^

##### ^1^Division of Clinical Immunology and Allergy and Center for Basic and Clinical Immunology Research (CISI), University of Naples Federico II, Naples, Italy; ^2^Department of Biomedical and Clinical Sciences L. Sacco, University of Milan, Milan, Italy; ^3^Hungarian Angioedema Center 3rd Department of Internal Medicine, Semmelweis University, Budapest, Hungary; ^4^CNR Institute of Experimental Endocrinology and Oncology “G. Salvatore”, Naples, Italy

###### **Correspondence:** Stefania Loffredo (stefanialoffredo@hotmail.com)

*Allergy, Asthma & Clinical Immunology* 2017,** 13(Suppl 2)**:O-9

**Introduction:** Phospholipase enzymes (PL) play central role in numerous cellular events including inflammation and immunoregulation by catalysing the lysis of phosphorylated lipids. Four distinct families of PL have been described and are named A, B, C and D. These enzyme family members are categorised based on the stereospecifically numbered sites within phospholipids where they promote cleavage. Secreted PLA_2_ (sPLA_2_) cleaves the sn-2 acyl chain, releasing arachidonic acid and lysophosphatidic acid. Upon downstream modification by cyclooxygenases, arachidonic acid is modified into active compounds called eicosanoids that include prostaglandins and leukotrienes, which are vascular permeability factors. Moreover, sPLA_2_, released in the blood and biological fluids, exert other biological effects relevant to the initiation and regulation of inflammatory and immune responses.

Lipoprotein-associated PLA2 (Lp-PLA_2_) also known as platelet-activating factor acetylhydrolase is a PLA_2_ that catalyzes the hydrolysis of acetyl ester at the sn-2 position of PAF. It has been shown that bradykinin modulates vascular permeability through the activation of PL. Therefore we supposed that PLA_2_ may have a role in pathogenesis of angioedema. The aim of this study was to analyze the plasma activity of PLA_2_ in patients with C1-INH-HAE.

**Methods:** 71 controls and 105 C1-INH-HAE patients were studied. PLA_2_ and LP-PLA_2_ activity was measured by fluorescent assay. Angiogenic factors [vascular endothelial growth factors (VEGF-A), angiopoietins 1 and 2] were evaluated by ELISA.

**Results:** Plasma activity of sPLA_2_ and LP-PLA_2_ were higher in C1-INH-HAE patients in remission than in controls. There was no correlation between the activity of sPLA_2_ and LP-PLA_2_. Interestingly, LP-PLA_2_ activity was higher in C1-INH-HAE patients experiencing more than 12 attacks per year than in those who had less than 12 attacks. sPLA_2_ activity was decreased in patients with higher Ang1 and Ang2 levels. Whereas, there was positive correlation between activity of LP-PLA_2_ and Ang1 and negative correlation between LP-PLA_2_ and Ang2 and Ang2/Ang1 ratio (an index of vascular permeability). Moreover, the activity of these mediators were decreased during attack compared with basal conditions.

**Conclusions:** Plasma activity of PLA_2,_ that alter vascular permeability through their activation by bradykinin and eicosanoid production, are increased in patients with C1-INH-HAE in remission and decreased during attacks. Based on these observations that support immunomodulator role of PLA_2_, we hypothesize that these enzymes along with an increased release of bradykinin, VEGFs and Angs, induce a state of “vascular pre-conditioning” that may change the threshold for development of angioedema attacks.

### O-10 An observation of contact system activation in the early angioedema suggests a systemic process prior to a local process

#### Arije Ghannam^1,2^, Samuel Luyasu^3^, Bertrand Favier^2^, Ludovic Martin^4,5^, Christian Drouet^2,4^

##### ^1^KininX SAS, Grenoble, 38000, France; ^2^GREPI UE7408, Université Grenoble Alpes, Grenoble, France; ^3^Cliniques du Sud Luxembourg, Arlon, Belgium; ^4^Centre de Référence des Angioedèmes (CREAK), Grenoble, France; ^5^Department of Dermatology, Angers University Hospital, Angers, France

###### **Correspondence:** Arije Ghannam (arije.ghannam@kininx.com)

*Allergy, Asthma & Clinical Immunology* 2017,** 13(Suppl 2)**:O-10

**Background:** The emergence of clinical angioedema (AO) is subsequent to the enzymatic production, AO attacks results from a local endothelial permeability in the affected tissue. Contact phase activation and bradykinin (BK) generation mediate this process.

According to the model established by Hofman et al., a systemic activation process of the contact system can result in localized angioedema attacks. Here we report an observation of patient with systemic contact phase activation earlier to localized AO.

**Materials and methods:** Citrate plasma of a patient was submitted for AO exploration. 3 samples were harvested at different times, (a) 13 h before attack during a regular consultation, (b) during attack, (c) 21 days after attack. C1 Inhibitor function, spontaneous amidase activity, kinin measurement and kininogen cleavage were performed as described previously [1–5].

The patient is a female subject, 24 years, without drugs, admitted for angioedema in emergency department during crisis: abdominal cramps, respiratory gene and AO of the eyelids.

**Results:** The subject does not carry the missense mutation 1032A/G of exon 9 of the *F12* gene. C1Inh function is normal. Biological analyses showed very important increased kinin forming with increased spontaneous amidase activity 306.9 nmol/mL min (reference 2.4–0.7 nmol/mL min) and decreased proenzyme activatability 523 nmol/mL min (reference 2422–4560 nmol/mL min) 13 h before the crisis; while during the crisis and local AO manifestations, proenzymes were reconstituted 5755 nmol/mL min and spontaneous amidase activity is still slightly increased 19.2 nmol/mL min. This was associated with kininogen (HK) cleavage for the two samples 13 h before crisis with 0 ng of native HK (nHK, references 44.5–186 ng/mL) and 99% of cleaved HK (cHK, reference 1–15.6%), and during crisis with 0 ng of nHK and 98% of cHK. In agreement with HK cleavage, bradykinin (BK) generation curve of plasma upon contact phase activation is greatly impaired.

The results of the sample at day 21 after crisis showed normalisation of precedent parameters (nHK 129 ng/mL, cHK 13%, BK generation, spontaneous amidase activity 11.2 nmol/mL min, proenzyme 5635 nmol/mL min).

**Conclusion:** The symptomatology of angioedema is posterior to the enzymatic activity that produces kinins; the delay of 24–36 h should be the time required for the permeability process.

Kinetics of biological events before crisis, in crisis, after restoration demonstrate that a systemic contact phase activation occurs prior to the local AO and clinical manifestation.

**Consent to publish:** Written consent to publish was obtained from the patient.

**References**

1. Drouet C, Alibeu C, Ponard D, Arlaud GJ, Colomb MG. A sensitive method to assay blood complement C1-inhibitor activity. Clin Chim Acta. 1988;174(2):121–30.

2. Ghannam A, Sellier P, Defendi F, Favier B, Charignon D, López-Lera A, et al. C1 inhibitor function using contact-phase proteases as target: evaluation of an innovative assay. Allergy. 2015;70(9):1103–11.

3. Defendi F, Charignon D, Ghannam A, Baroso R, Csopaki F, Allegret-Cadet M, et al. Enzymatic assays for the diagnosis of bradykinin-dependent angioedema. PLoS ONE. 2013;8(8):e70140.

4. Baroso R, Sellier P, Defendi F, Charignon D, Ghannam A, Habib M, et al. Kininogen cleavage assay: diagnostic assistance for kinin-mediated angioedema conditions. PLoS ONE. 2016;11(9):e0163958.

5. Molinaro G, Cugno M, Perez M, Lepage Y, Gervais N, Agostoni A et al. Angiotensin-converting enzyme inhibitor-associated angioedema is characterized by a slower degradation of des-arginine9-bradykinin. J Pharmacol Exp Ther. 2002;303:232–7.

### O-11 Novel high-resolution follow-up of the coagulation and fibrinolytic parameters in a single angioedematous attack of a C1-INH-HAE patient

#### Nóra Veszeli^1,2^, Erika Kajdácsi^1^, Kinga Viktória Kőhalmi^1,2^, György Temesszentandrási^2^, Katalin Várnai^3^, László Cervenak^1^; Lilian Varga^1,2^, Henriette Farkas^1,2^

##### ^1^Research Laboratory, 3rd Department of Internal Medicine, Semmelweis University, Budapest, 1125, Hungary; ^2^Hungarian Angioedema Center, 3rd Department of Internal Medicine, Semmelweis University, Budapest, 1125, Hungary; ^3^Department of Laboratory Medicine, Semmelweis University, Budapest, 1085, Hungary

###### **Correspondence:** Nóra Veszeli (veszeli.nora88@gmail.com)

*Allergy, Asthma & Clinical Immunology* 2017,** 13(Suppl 2)**:O-11

**Background:** Hereditary angioedema due to C1-inhibitor deficiency (C1-INH-HAE) is a potentially life-threatening rare disease, characterized by recurring and spontaneously resolving edematous attacks. Previously many studies published on the activation of the plasma enzyme systems during edematous attacks, nevertheless kinetic follow-up has never been performed. For the first time, we aimed to study the kinetics of parameters in the coagulation and fibrinolytic systems in a spontaneously resolved edematous attack of a C1-INH-HAE patient. Furthermore, we aimed to study the kinetics of these parameters in a healthy subject during a 24-h period, served as control.

**Materials and methods:** In a 56-year-old female with C1-INH-HAE we monitored the severity of the symptoms during the entire observation period and altogether twelve blood samples were obtained. Blood samples collected from a healthy volunteer at 5 different times during a 24-h period served as a control. We measured factors XI and XII activities (FXIa, FXIIa), as well as concentration of prothrombin fragment 1 + 2 (F1 + 2), thrombin-antithrombin (TAT)-complex, D-dimer, fibrinogen.

**Results:** After a 24-h symptom-free period and another 19-h prodromal period, the patient had a 29-h-long edematous attack in multiple skin locations, and was followed up for another day. It is remarkable that during prodromal stage—which was characterized by erythema marginatum—the levels of D-dimer, F1 + 2 and TAT-complex were as constantly low as those levels measured in the healthy control. Levels of F1 + 2 and TAT-complex were significantly elevated at the moment of the onset of edematous symptoms whereas level of D-dimer was elevated after 6 h. Levels of all three parameters reached maximum 12 h after reaching the maximum severity score. Highest level of D-dimer was almost 100-fold higher than the levels measured during prodromal stage. Fibrinogen levels were constantly elevated during prodromal stage whereas during edematous attack, fibrinogen levels were similarly low as levels were measured in the healthy control. In the healthy control subject, all measured parameters were stable during 24-h observational period.

**Conclusions:** This study was a part of a project aimed to the better understanding of the mechanisms leading to the onset and to the resolution of edematous attack. Real-time monitoring of F1 + 2 and TAT complex suggest that thrombin contribute to the development of edema formation. We confirmed that D-dimer is a prominent biomarker of an ongoing edematous attack. Elevated levels of fibrinogen before the onset edematous symptoms raise the possibility of using it as a predictive marker.

**Consent to publish:** Both subjects gave written informed consent for the publication.

This study was supported by the ÚNKP-16-3 New National Excellence Program of the Ministry of Human Capacities”.

### O-12 Population pharmacokinetics of subcutaneous versus intravenous C1-INH concentrate for the prevention of HAE attacks

#### Bruce Zuraw^1^, Marco Cicardi^2^, Annette Feussner^3^, Michael A. Tortorici^4^, Dipti Pawaskar^4^

##### ^1^University of California Medical Center, San Diego, CA, USA; ^2^Università Degli Studi Di Milano, Milan, Italy; ^3^CSL Behring, Marburg, Germany; ^4^CSL Behring, King of Prussia, PA, USA

###### **Correspondence:** Bruce Zuraw (bzuraw@AD.UCSD.EDU)

*Allergy, Asthma & Clinical Immunology* 2017,** 13(Suppl 2)**:O-12

**Background:** The COMPACT program of studies showed that a high-concentrate subcutaneous (SC) formulation of C1-INH (CSL830) was safe and effective in preventing hereditary angioedema attacks (HAEAs). In this analysis, we aim to use data from three studies to develop a population pharmacokinetics (POPPK) model to characterise the disposition of C1-INH functional activity (C1-INH[f]) after intravenous (IV) and subcutaneous (SC) administration.

**Materials and methods:** The population C1-INH(f) data in the subjects treated with C1-INH were analyzed by nonlinear mixed effects modeling using the package NONMEM (v7.2) to characterise the pharmacokinetics (PKs) of C1-INH concentrate. PK parameters such as clearance (CL), volume of distribution (Vd), bioavailability (F) and baseline C1-INH(f) were estimated using the first-order conditional estimation method.

**Results:** A total of 124 subjects (108 with HAE and 16 healthy volunteers) were included in the PK analysis dataset, which comprised of a total of 2103 C1-INH(f) observations. C1-INH(f) following administration of C1-INH was adequately described by a one-compartment model with first-order absorption and elimination, with inter-individual variability estimated for all the parameters tested. Separate baseline parameters were included in the model for HAE patients and healthy volunteers. The population mean bioavailability of C1-INH was 0.427. Body weight effect on CL of C1-INH(f) was included in the final model with the weight exponents on CL estimated to be 0.738. The population mean (95% CI) CL and Vd were estimated to be approximately 0.830 IU/h∙% (0.727, 0.942 IU/h∙%) and 43.3 IU/% (35.1, 51.5 IU/%), respectively. The final population PK model was used to simulate C1-INH(f) versus time profiles in 1000 virtual HAE patients. The prediction revealed higher C_trough_ with 40 IU/kg (40.2%) and with 60 IU/kg (48.0%) compared with 1000 IU IV (~30%) and a lower peak-to-trough ratio with a more consistent elevation of the C1-INH(f) for the 40 and 60 IU/kg SC doses compared to the 1000 IU IV dose.

**Conclusions:** C1-INH(f) was well described by a one-compartment model with first order absorption. Body weight was a significant covariate that affected CL. The PK profile for SC administration of C1-INH shows higher C_trough_ with a more consistent and evenly distributed plasma level of C1-INH(f) compared to the profile for IV administration.

### O-13 Pharmacodynamic effects of subcutaneous C1-INH for the prevention of HAE attacks

#### Marco Cicardi^1^, Huamin Henry Li^2^, John Anderson^3^, Jonathan A. Bernstein^4^, Henriette Farkas^5^, Ying Zhang^6^, Dipti Pawaskar^6^, Ingo Pragst^7^, Michael A. Tortorici^6^, on behalf of COMPACT investigators

##### ^1^Università Degli Studi Di Milano, Milan, Italy; ^2^Institute for Asthma and Allergy, Chevy Chase, MD, USA; ^3^Alabama Allergy & Asthma Centre, Birmingham, AL, USA; ^4^Bernstein Allergy Group, Cincinnati, OH, USA; ^5^Semmelweis University, Budapest, Hungary; ^6^CSL Behring, King of Prussia, PA, USA; ^7^CSL Behring, Marburg, Germany

###### **Correspondence:** Marco Cicardi (marco.cicardi@unimi.it)

*Allergy, Asthma & Clinical Immunology* 2017,** 13(Suppl 2)**:O-13

**Background:** The Phase 3 COMPACT trial showed that a high-concentrate subcutaneous (SC) formulation of C1-INH (CSL830) provided dose-dependent, physiologically-relevant increases in trough C1-INH functional activity (C1-INH[f]), and demonstrated its safety and effectiveness in preventing hereditary angioedema attacks (HAEAs). Data from this trial was used to (1) study an exposure–response (E–R) relationship between C1-INH(f) and risk of an HAEA and (2) explore the relationship between C1-INH(f) and levels of complement factor 4 (C4), a marker of activation of the complement system.

**Materials and methods:** Data from the COMPACT program were used to develop a population E–R model. An interval-censored repeated time-to-event (TTE) model was developed that enabled the C1-INH(f) to be directly related to HAEAs. The final model included two components, a baseline hazard and a non-linear drug effect. The relationship between C1-INH(f) and C4 antigen was visually inspected in an exploratory manner.

**Results:** Ninety subjects from the COMPACT Phase 3 study experienced 1425 censored HAEAs. A strong, inverse relationship between C1-INH(f) and HAEAs was found, with the final TTE model estimating that 50, 70 and 90% reductions in relative risk of an HAEA correspond to C1-INH(f) of 33.1, 40.3 and 63.1%, respectively. Based on the trough C1-INH(f) levels over a steady-state dosing interval (C_trough_) for 40 IU/kg (40.2%) and 60 IU/kg (48.0%) C1-INH, it is estimated that 50 and 67% of a HAE population would see a 70% reduction in relative risk of HAEAs, respectively. Moreover, C1-INH(f) and C4-antigen levels appear to have a linear relationship in HAE subjects in levels of C1-INH(f) up to ~50%, after which the relationship shows signs of saturability.

**Conclusions:** There is a close inverse relationship between C1-INH(f) after SC C1-INH injection and the occurrence of HAEAs. Simulations based on E–R suggest that the prevention of HAEAs is maximised when C1-INH(f) is restored to the normal range (>70%). There is a linear relationship between C1-INH(f) and C4-antigen levels up to ~50% after which saturability is observed. This suggests that there may be potential diagnostic value in monitoring C1-INH(f) and C4 levels during prophylaxis with C1-INH.

### O-14 Safety and efficacy of a C1 inhibitor for the prevention of hereditary angioedema attacks in children: interim results of a Phase 3 study

#### Emel Aygören-Pürsün^1^, Kraig Jacobson^2^, Dumitru Moldovan^3^, Jim Christensen^4^, Arthur Van Leerberghe^5^, Yi Wang^6^, Jennifer Schranz^6^, Inmaculada Martinez-Saguer^7^, Daniel Soteres^8^

##### ^1^Department for Children and Adolescents, Angioedema Centre, University Hospital Frankfurt, Goethe University, Frankfurt, Germany; ^2^Oregon Allergy Associates, Allergy and Asthma Research Group, Eugene, OR, USA; ^3^University of Medicine & Pharmacy, Mures County Hospital, Tirgu Mures, Romania; ^4^Nevada Access to Research & Education Society, Las Vegas, NV, USA; ^5^Shire, Brussels, Belgium; ^6^Shire, Lexington, MA, USA; ^7^Hemophilia Center Rhein Main, Mörfelden-Walldorf, Germany; ^8^Asthma & Allergy Associates, Colorado Springs, CO, USA

###### **Correspondence:** Emel Aygören-Pürsün (Aygoeren-Puersuen@em.uni-frankfurt.de)

*Allergy, Asthma & Clinical Immunology* 2017,** 13(Suppl 2)**:O-14

**Background:** Cinryze, a human plasma-derived C1 inhibitor (C1-INH) is approved for routine prophylaxis against hereditary angioedema (HAE type I and II) attacks in adults, adolescents, and children. We present interim efficacy and safety results as well as the pharmacokinetics and pharmacodynamics of two doses of intravenous (IV) C1-INH in patients 6–11 years of age.

**Methods:** This is an ongoing randomized, phase 3, multi-centre, single-blind, crossover study (NCT02052141). We present data from 6 patients who completed the study. Following a 12-week baseline observation period (BOP), patients were randomised to receive 500U or 1000U IV C1-INH every 3–4 days for 12 weeks, and then switched to the other dose for 12 weeks. The primary efficacy endpoint was the number of monthly attacks in a treatment period. Secondary efficacy endpoints included attack severity and the number of attacks requiring acute treatment. Clinical and laboratory safety were evaluated. Blood samples were collected before and 1 h after doses 1, 12, and 24 to measure functional C1-INH activity, C1-INH antigen, and C4.

**Results:** Six patients (all female with HAE type I and a median age of 10.5 years) had a mean (SD) of 2.26 (1.62) attacks per month during the BOP. The mean (SD) difference in the number of attacks between the BOP and treatment periods was −1.89 (−1.31) and −1.89 (−1.11) for the 500 and 1000 U doses, respectively, i.e. an 84.8% (20.1) and 88.1% (13.4) reduction in the number of attacks. The safety profile of either dose was comparable. No deaths, serious adverse events (AEs), thrombotic or thromboembolic events occurred. Frequently reported treatment-related AEs included fatigue and irritability. Baseline-adjusted C1-INH functional activity (corrected for its pre-first dose level) observed pre-dose 12 (mean [SD] of 0.145 [0.152] U/ml for 500 U and 0.210 [0.098] U/mL for 1000 U; normal range: >0.68 U/ml) was approximately maintained to dose 24. An hour post-dosing, C1-INH functional activity and antigen concentration increased two-fold or more. Mean (SD) baseline-adjusted C1-INH antigen concentrations pre-dose 12 were 0.039 (0.024) g/L for 500 U and 0.044 (0.032) g/L for 1000 U (normal range: 0.21–0.39 g/L). For dose 12, C4 concentration was variable with a mean (SD) of 36.2 (36.7) mg/L for 500 U and 49.0 (42.1) mg/L for 1000 U, (normal range: 160–700 mg/L).

**Conclusion:** In this 24-week study, twice-weekly IV administration of 500 and 1000 U C1-INH in 6 children aged 6–11 years was safe and well-tolerated, and demonstrated efficacy in reducing HAE attack frequency.

### O-15 BCX7353, a once-daily oral kallikrein inhibitor, is effective and safe in the prophylaxis of acute attacks in patients with hereditary angioedema: results from the first interim analysis of the APeX-1 study

#### Emel Aygören-Pürsün^1^, Urs Steiner^2^, Vesna Grivcheva Panovska^3^, Henriette Farkas^4^, William Rae^5^, Werner Aberer^6^, Aarnoud Huissoon^7^, Anette Bygum^8^, Markus Magerl^9^, Andrea Zanichelli^10^, Jochen Graff^11^, Hilary Longhurst^12^, Ramón Lleonart^13^, Lei Fang^14^, Melanie Cornpropst^15^, Desiree Clemons^15^, Amanda Mathis^15^, Phil Collis^15^, Sylvia Dobo^15^, William P. Sheridan^15^, Marcus Maurer^9^, Marco Cicardi^10^

##### ^1^Department for Children and Adolescents, University Hospital Frankfurt, Germany; ^2^Department of Clinical Immunology, University Hospital Zurich, Zurich, Switzerland; ^3^PHI University Clinic of Dermatology, Skopje, Republic of Macedonia; ^4^Hungarian Angioedema Center, 3rd Department of Internal Medicine, Semmelweis University, Budapest, Hungary; ^5^The University Hospital Southampton NHS Foundation Trust, Southampton, UK; ^6^Medizinische Universität Graz, Graz, Austria; ^7^Heart of England NHS Foundation Trust, Birmingham, UK; ^8^JHAE Centre Denmark, Odense University Hospital, Odense, Denmark; ^9^Department of Dermatology and Allergy, Charité-Universitätsmedizin, Berlin, Germany; ^10^University of Milan Department of Biomedical and Clinical Sciences, Milan, Italy; ^11^Projektgruppe Translationale Medizin & Pharmakologie TMP, Fraunhofer-Institut für Molekularbiologie und angewandte Oekologie IME, Frankfurt, Germany; ^12^Department of Immunology Barts Health NHS Trust, London, UK; ^13^Hospital Universitari de Bellvitge, Barcelona, Spain; ^14^PharStat, Inc., Durham, NC, USA; ^15^BioCryst Pharmaceuticals, Durham, NC, USA

###### **Correspondence:** Emel Aygören-Pürsün (Aygoeren-Puersuen@em.uni-frankfurt.de)

*Allergy, Asthma & Clinical Immunology* 2017,** 13(Suppl 2)**:O-15

**Background:** BCX7353 is a potent once daily oral kallikrein inhibitor. A Phase 2, double-blind, dose-ranging, placebo-controlled, 3-part, parallel-group study (APeX-1) evaluated the efficacy, safety, pharmacokinetics and pharmacodynamics of BCX7353 as a prophylactic treatment in HAE patients. A planned interim analysis of a subset of Part 1 is reported (28 of 36 planned subjects).

**Materials and methods:** In Part 1, patients with HAE Type I or II with a recent history of frequent angioedema attacks were randomized 1:1 to receive 28 days of treatment with BCX7353 350 mg QD or placebo. Parts 2 and 3 will include an analysis of lower doses (125 and 250 mg [Part 2] and 62.5, 125 and 250 mg [Part 3]). Subjects recorded details of HAE attacks in a diary. Efficacy was assessed by the number of attacks over the entire and effective dosing period (EDP, Days 8–29) when BCX7353 steady-state conditions were achieved. Plasma samples were drawn for BCX7353 concentrations and kallikrein inhibition at steady state. Safety was monitored by adverse events (AEs), laboratory assessments, vital signs, physical exam findings and ECGs.

**Results:** Twenty-eight subjects with a mean of 1.0 HAE attacks per week were enrolled and included in the interim analysis. Four subjects were excluded from the per-protocol (PP) population: two subjects on BCX7353 discontinued early (non-treatment emergent liver enzyme elevations and gastroenteritis with liver enzyme elevations) and two subjects (one per treatment) did not have confirmed Type I or II HAE. The least-squares mean HAE attack rates during the EDP in the PP population were 0.92 (placebo) and 0.34 (BCX7353) attacks per week, translating to a reduction in attacks of 63% (p = 0.006). Four of 11 subjects (36%) were attack-free on BCX7353 during the EDP compared to 1 of 13 on placebo (7.7%; PP population). All trough concentrations drawn on BCX7353-treated subjects at steady-state exceeded the pre-defined minimum therapeutic target (fold range 2.3–7.0, mean 4.7). Mean ex-vivo plasma kallikrein activity was suppressed by an average of 82–89%, and this was sustained over the dosing interval. There were no SAEs and no severe drug-related AEs. BCX7353-treated subjects had a numerically higher number of mild to moderate gastrointestinal AEs; additionally, BCX7353-related gastrointestinal AEs may have been reported as HAE attacks.

**Conclusions:** Once-daily BCX7353 was associated with a clinically meaningful, statistically significant reduction in attacks in subjects with HAE. BCX7353 was generally safe and well tolerated. These findings support the ongoing evaluation of a range of doses of BCX7353 for prophylaxis of angioedema attacks in HAE patients.

### O-16 A Phase 3 open-label extension study of the efficacy and safety of lanadelumab for the prevention of angioedema attacks in patients with hereditary angioedema: trial design

#### Marc A. Riedl^1^, Jonathan A. Bernstein^2^, Timothy Craig^3^, Aleena Banerji^4^, Markus Magerl^5^, Marco Cicardi^6,7^, Hilary J. Longhurst^8^, Mustafa Shennak^9^, William Yang^10^, Jennifer Schranz^11^, Jovanna Baptista^11^, Paula Busse^12^

##### ^1^University of California-San Diego School of Medicine, La Jolla, CA, 92122, USA; ^2^Department of Internal Medicine/Allergy Section Cincinnati, University of Cincinnati College of Medicine, Cincinnati, OH, 45267, USA; ^3^Department of Medicine and Pediatrics, Penn State Hershey Allergy, Asthma and Immunology, Hershey, PA, 17033, USA; ^4^Division of Rheumatology, Allergy and Immunology, Department of Medicine, Massachusetts General Hospital, Harvard Medical School, Boston, MA, 02114, USA; ^5^Department of Dermatology and Allergy, Charité-Universitätsmedizin Berlin, Berlin, Germany; ^6^Department of Biomedical and Clinical Sciences, Luigi Sacco, University of Milan, Milan, Italy; ^7^Luigi Sacco Hospital Milan, Milan, Italy; ^8^Department of Immunology, Barts Health NHS Trust, London, E1 2ES, UK; ^9^Triumpharma Inc., Amman, Jordan; ^10^Ottawa Allergy Research Corporation, University of Ottawa Medical School, Ottawa, ON, K1G 6C6, Canada; ^11^Shire, Lexington, MA, 02421, USA; ^12^Division of Clinical Immunology and Allergy, Department of Medicine, Icahn School of Medicine at Mount Sinai, New York, NY, 10029, USA

###### **Correspondence:** Marc A. Riedl (mriedl@ucsd.edu)

*Allergy, Asthma & Clinical Immunology* 2017,** 13(Suppl 2)**:O-16

**Background:** Lanadelumab (DX-2930/SHP-643) is a long-acting, highly-specific, potent, human monoclonal antibody targeting plasma kallikrein that received fast track and breakthrough therapy designations. Results from a Phase 1b study (NCT02093923) did not identify safety signals and supported efficacy of lanadelumab in preventing hereditary angioedema (HAE) attacks. A pivotal randomized, double-blind (DB), placebo-controlled, parallel arm study (NCT02586805) is ongoing, and an open-label extension (OLE; NCT02741596) is currently enrolling patients (pts). Here, we describe the design of a Phase 3 OLE study to evaluate the long-term safety and efficacy of lanadelumab for prevention of angioedema attacks in patients with HAE.

**Materials and methods:** This OLE will include pts (≥12 years old; Type 1/2 HAE) rolling over from the DB study and an additional 50–100 pts who did not participate in the DB study (non-rollover). The non-rollover population will include pts switching to lanadelumab from another prophylactic therapy. Rollover pts will initially receive a single 300 mg subcutaneous dose of lanadelumab and will not receive another dose until after their first HAE attack. Thereafter, lanadelumab 300 mg q2 weeks will be administered until Day 350, followed by a 4-week safety follow-up. Non-rollover patients will be dosed q2 weeks regardless of their first attack. Pts may qualify to self-administer lanadelumab. The primary objective of the OLE will be to assess long-term safety. In the phase 1b study, 25% pts had local adverse effects following lanadelumab treatment vs 23.1% following placebo. Secondary objectives include evaluation of efficacy (time to first HAE attack to determine outer bounds of the dosing interval, attack rate, number attacks requiring acute treatment, are moderate/severe, or are associated with high-morbidity). Lanadelumab 300 and 400 mg was associated with a 100 and 88% reduction in attacks, respectively, in the Phase 1b study. Immunogenicity, pharmacokinetics/pharmacodynamics, quality of life, characteristics of breakthrough attacks, self-administration and safety/efficacy in pts switching to lanadelumab from another prophylactic therapy will be evaluated.

**Results:** Results of the OLE are expected in 2018.

**Conclusions:** Results of this study will provide additional important data on the long-term safety, efficacy and dosing frequency of lanadelumab, a first-in-class subcutaneous therapy for prevention of angioedema attacks in patients with HAE.

### O-17 Safety study of ATN-249, a new oral kallikrein inhibitor for hereditary angioedema

#### Ira Kalfus, Andrew McDonald, Shawn Qian

##### Attune Pharmaceuticals, LLC, New York City, NY 10019, USA

###### **Correspondence:** Ira Kalfus (ikalfus@aol.com)

*Allergy, Asthma & Clinical Immunology* 2017,** 13(Suppl 2)**:O-17

**Background:** Hereditary angioedema (HAE) is a rare, potentially life-threatening disease characterized by acute skin and mucosal oedema. Current treatments of HAE are limited by route of administration and adverse events, since all HAE drugs are administered intravenously or subcutaneously, and may be associated with drug-specific adverse effects. There is a need for safe orally-administered therapies that control plasma kallikrein activity, prevent HAE attacks, and are well-tolerated. Approximately 900 compounds were screened on the basis of chemical structure, selectivity for plasma kallikrein, and kallikrein inhibition. This study evaluated the safety of ATN-249, a novel, potent, and selective orally administered plasma kallikrein inhibitor that potentially treats HAE by blocking kallikrein-mediated production of bradykinin.

**Materials and methods:** Genotoxicity was evaluated in bacterial reverse mutation, chromosomal aberration, and micronucleus assays. Good laboratory practice (GLP) repeat dose (28-day) toxicity was evaluated in rats and monkeys. Metabolite profiles were measured by dose recovered in intact rats and bile duct cannulated (BDC) rats. Safety pharmacology was evaluated in the functional observational battery (FOB) assessment in rats and in cardiovascular and respiratory telemetry studies in monkeys.

**Results:**

- *Genotoxicity* No genotoxicity was noted in bacterial reverse mutation, chromosomal aberration, and micronucleus assays.
- *Toxicity* No noteworthy findings on clinical signs, hematology, coagulations, serum chemistry, urinalysis, and gross necropsy in GLP 28-day rat and monkey toxicity studies at doses up to 100 mg/kg/day.
- *Metabolism* Following a single oral administration:
  - In intact rats, 100% of ATN-249 was recovered within 0–168 h, with 99% in feces.
  - In BDC rats, 97% of ATN-249 was recovered within 0–72 h; with 52% recovered in feces and 39% recovered in bile.
  - Previously, bioavailability was shown to be >50% in rats.
- *Safety pharmacology* No adverse effect was observed in rat FOB studies or monkey cardiovascular and respiratory studies.

**Conclusions:** Studies demonstrating the absence of genotoxicity, absence of adverse events in rat and monkey toxicity, high bioavailability, comprehensive recovery following oral administration, and absence of adverse events in safety pharmacology suggest that ATN-249’s safety profile is desirable and appropriate for further clinical evaluation. These results, along with previously published high potency and high selectivity results, suggest ATN-249 has a wide therapeutic window with once-daily dosing potential, and may be a novel, safe, potent, and selective orally-administered plasma kallikrein inhibitor for treatment of hereditary angioedema (HAE).

### O-18 FXIIa-mediated kallikrein activity discriminates between HAE and normal plasma samples and is a pharmacodynamic marker for CSL312

#### Anthony Roberts, Con Panousis, Tim Green, Andreas Gille

##### Global Research, CSL Limited, Parkville, VIC, Australia

###### **Correspondence:** Anthony Roberts (anthony.roberts@csl.com.au)

*Allergy, Asthma & Clinical Immunology* 2017,** 13(Suppl 2)**:O-18

**Background:** CSL312 is a FXIIa antagonist fully human monoclonal antibody in development for Hereditary Angioedema (HAE). A Phase 1 study in healthy subjects is underway to assess safety, tolerability and pharmacokinetics. FXIIa-mediated kallikrein activity is being used as an exploratory biomarker to assess CSL312 pharmacodynamics.

**Methods:** Plasma samples were diluted in buffer containing dextran sulphate to initiate contact activation. Chromogenic substrate, H-D-Pro-Phe-Arg-pNA (S-2302) was used to monitor kallikrein enzymatic activities in normal human plasma and HAE patient plasma in the presence of varying CSL312 concentrations. Rates of substrate cleavage were derived from the changes in absorbance over time.

**Results:** A fit-for-purpose quasi-quantitative assay of FXIIa-mediated kallikrein activity has been developed. Precision both within and between assays was well within 30% CV. The assay is sensitive to spiked concentrations of CSL312 in the range of 1–10 µg/mL. The assay also discriminates HAE and healthy plasma samples. Less than 10 µg/mL of CSL312 added to HAE plasma restored amidolytic activity to levels seen in normal subjects. The CSL312 concentration needed to treat HAE samples to achieve a target kallikrein activity may be used to predict effective CSL312 doses in HAE patients. Data will also be presented showing characterisation of different HAE patient samples using this assay.

**Conclusions:** CSL312 is a potent inhibitor of FXIIa-mediated kallikrein activity in normal and HAE plasma. This assay shows promise to monitor CSL312 effects and inform clinical dosing regimens.

### O-19 Putative role of KLKB1 gene on HAE pathophysiology

#### Renan P. Martin, Rafael Filippelli-Silva, Camila L. Veronez, João B. Pesquero

##### Department of Biophysics, UNIFESP, São Paulo, São Paulo, 04039-032, Brazil

###### **Correspondence:** Renan P. Martin (renan.paulo.martin@gmail.com)

*Allergy, Asthma & Clinical Immunology* 2017,** 13(Suppl 2)**:O-19

Hereditary angioedema (HAE) is an autosomal dominant inherited disease characterized by painful edema episodes with variable localization and severity. Most of diagnosed patients present either mutation on C1 inhibitor gene (SERPING1), with abnormal levels or functionality (HAE type I and II, respectively) or Factor 12 (FXII) gene (HAE-FXII). These mutations leads to exacerbated bradykinin (BK) production, the peptide responsible for edema formation. In addition, there are several HAE patients of unknown molecular etiology (HAE-U), presenting same symptoms but no mutation in both classical genes. Symptoms are widely variable in patients presenting the same mutation, even in the same family. This variability highlights the role of several other modulator genes on HAE pathophysiology. In order to determine these candidates, 15 genes were sequenced in patients with HAE-U using next generation sequencing (NGS) method. From the genes analysed, KLKB1 seems to participate as a causative gene, wherein two missense mutations were identified on the HAU-U patients. Those mutations were submitted to “in silico” analysis. Mutation p.Gln442Pro was classified as benign on Mutation Taster, PolyPhen2 and SIFT, whereas, Ingenuity analysis revels a gain of function for this mutation. The mutation p.Asp619Glu was classified on “in silico” analysis as probably pathogenic by Mutation Taster, possible pathogenic on PolyPhen2 and tolerable on SIFT. In addition, the two variants were visualized on YASARA software, in order to determine possible intra-molecular interactions. Gln442 seems to make a hydrogen bound with Val444 residue, but this interaction occurs on the C-terminal carbon. Therefore, substitution on amino-acid side chain might not play a major role on this interaction. In the case of Asp619, it is highly conserved among several species and could interact with Lys507 residue. This pocket is located at helix 5 close to disulfide bound, responsible for maintenance of light and heavy chains linked, after FXIIa cleavage. Changes in this pocket could lead the protein to assume a new conformation, modifying interactions between kallikrein and FXIIa, either favoring or hindering its activation. Thus, the present findings improve the knowledge on HAE disease field pointing KLKB1 as new gene ascribed to HAE pathophysiology, which improves HAE diagnoses and patients life quality.

This study was supported by grants of FAPESP, CNPq, and CAPES.

### O-20 Genotyping unknown hereditary angioedema (U-HAE) and idiopathic non-histaminergic acquired angioedema (InH-AAE): preliminary evidence of pathogenic variants

#### Maria Zamanakou^1^, Gedeon Loules^1^, Dorottya Csuka^2^, Maria Bova^3^, Fotis Psarros^4^, Faidra Parsopoulou^5^, Matthaios Speletas^5^, Davide Firinu^6^, Tiziana De Pasquale^7^, Alessandra Zoli^8^, Anna Radice^9^, Stefano Pizzimenti^10^, Andrea Zanichelli^11^, Emmanouil Manoussakis^12^, George N. Konstantinou^13^, Henriette Farkas^2^, Marco Cicardi^11^, Anastasios E. Germenis^5^

##### ^1^CeMIA SA, Larissa, Greece; ^2^Hungarian Angioedema Center, 3rd Department of Internal Medicine, Semmelweis University, Budapest, Hungary; ^3^Department of Translational Medicine, University of Naples, Naples, Italy; ^4^Department of Allergology, Navy Hospital, Athens, Greece; ^5^Department of Immunology & Histocompatibility, School of Health Sciences, Faculty of Medicine, University of Thessaly, Larissa, Greece; ^6^Department of Medical Sciences and Public Health, University of Cagliari, Cagliari, Italy; ^7^Department of Allergy, Hospital of Civitanova Marche, Civitanova Marche, Italy; ^8^Department of Clinical Immunology, Ospedali Riuniti, Ancona, Italy; ^9^Unit of Immunoallergology, Azienda Ospealiera Universitarta Careggi, Florence, Italy; ^10^Department of Allergy, University of Torino, Turin, Italy; ^11^Department of Biomedical and Clinical Sciences Luigi Sacco, University of Milan, Milan, Italy; ^12^Allergy Unit, 2nd Pediatric Clinic, University of Athens, “P & A Kyriakou” Children’s Hospital, Athens, Greece; ^13^Department of Allergy and Clinical Immunology, 424 General Military Training Hospital, Thessaloníki, Greece

###### **Correspondence:** Anastasios E. Germenis (agermen@med.uth.gr)

*Allergy, Asthma & Clinical Immunology* 2017,** 13(Suppl 2)**:O-20

**Background:** Despite the fact that cases of U-HAE and InH-AAE are increasingly recognized, the pathogenesis of these diseases remains still unknown. The aim of this study was to identify genetic alterations possibly linked with this type of angioedema.

**Materials and methods:** 29 unrelated patients with idiopathic recurrent angioedema (males/females 8/21, aged 43 ± 21 years) diagnosed like U-HAE (18) or InH-AAE (11) were submitted to targeted genotyping focused on 55 genes involved in bradykinin metabolism and function, including *SERPING1* and *F12* genes. 61 unrelated patients with HAE due to C1-inhibitor deficiency (C1-INH-HAE) were genotyped as controls. A next-generation sequencing (NGS) platform (Ampliseq custom panel, Thermo Scientific) was developed by which all coding regions and exon-intron splice junctions of these genes (minimum coverage 90%) were analyzed. Analysis of primary data was conducted with Ion Reporter software v.5.2 (Thermo Scientific). Polymorphisms (UCSC Common SNPs) for which no disease associations are reported in the ClinVar database were excluded and pathogenicity of variants was predicted by bioinformatics analysis using SIFT and PolyPhen tools, in comparison to their global (1000 Genomes Global Minor Allele Frequency, ExAC) and European frequency (5000 Exomes European Minor Allele Frequency).

**Results:** In 24/29 patients various heterozygous or homozygous genetic alterations of the analyzed genes were detected, alone (8) or in combination (16). These alterations represent either unreported or extremely rare variants some of which were bioinformatically predicted as deleterious. Every one of these variants was found only once, except for the p.Ile342Thr of *F13B* (3), the p.Val266Ile of *DPP4* (2) and the p.Lys330Glu of *PLG* (2). Nearly none of these variants was detected after genotyping 61 patients with C1-INH-HAE. Functional polymorphisms of other genes were also detected combined or not with the above variants.

**Conclusions:** We provide evidence indicating the existence of a heterogeneous genetic background linked with 72% of U-HAE and 100% of InH-AAE cases. Family segregation and/or functional studies are required for the pathogenicity or the disease modifying effect of these genetic alterations to be confirmed. As far as some of the detected alterations may affect the concentration of coded enzymes, their carriage has to be taken into account when biochemical studies of U-HAE or InH-AAE are performed.

### O-21 Hereditary angioedema with normal C1 inhibitor: a cohort of Italian patients

#### Maria Bova^1^, Valeria Bafunno^2^, Andrea Zanichelli^3^, Davide Firinu^4^, Vincenzo Montinaro^5^, Tiziana Maria Angela De Pasquale^6^, Suffritti Chiara^3^, Mauro Cancian^7^, Alessandra Zoli^8^, Maurizio Margaglione^2^, Marco Cicardi^3^

##### ^1^Department of Translational MedicalSciences, University of Naples, Federico II, Naples, Italy; ^2^Medical Genetics, Department of Clinical and Experimental Medicine, University of Foggia, Foggia, Italy; ^3^Department of Biomedical and Clinical Sciences LuigiSacco, University of Milan, Milan, Italy; ^4^Department of Medical Sciences, University of Cagliari, Cagliari, Italy; ^5^Division of Nephrology, University of Bari, Bari, Italy; ^6^Civitanova Marche Hospital, Civitanova Marche, Italy; ^7^Department of Medicine, University of Padua, Padua, Italy; ^8^Department of Clinical Immunology, Ospedali Riuniti, Ancona, Italy

###### **Correspondence:** Maria Bova (bovamaria@virgilio.it)

*Allergy, Asthma & Clinical Immunology* 2017,** 13(Suppl 2)**:O-21

**Rationale:** Hereditary angioedema with normal C1-inhibitor (nC1-INH-HAE) with and without factor XII mutations (FXII-HAE and U-HAE respectively) are familial disorders. We present the genetic and clinical features of patients with nC1-INH-HAE followed up in centers of the Italian Network for Angioedema (ITACA).

**Methods:** 105 patients with personal or family history of angioedema and normal plasma levels of C1 inhibitor were studied. C1-INH function was measured by chromogenic assay; C1-INH and C4 antigen levels were measured by immunologic assay.

All patients were investigated for mutations in the whole F12 gene coding region by direct DNA sequencing.

**Results:** 85 patients had angioedema symptoms. Of these patients, 22 females (median age 41.1 years, range 12–78), belonging to 9 unrelated families, had the same mutation in F12 gene, leading to the most common disease-causing aminoacid substitution, p.Thr309Lys. They were diagnosed as FXII-HAE. The haplotype analysis by using intragenic SNPs confirmed the hypothesis of a common founder. 20 subjects (11 males) in 8 FXII-HAE families were asymptomatic carriers of the same mutation.

63 patients (37 females; median age 43.53 years, range 12–81) had history of angioedema in their 39 families and no mutation in F12 gene. They were diagnosed as U-HAE. Sequencing analysis revealed the presence of different SNPs that have been previously described as not affecting protein activity or function.

Accordingly, the minimum prevalence of FXII-HAE and U-HAE in Italy in 2016 is 37:59,394,000 inhabitants and 60:59,394,000 respectively, equivalent to 1:1,605,243 for FXII-HAE and 1:989.900 for U-HAE.

**Conclusions:** This is the first report on the complete sequencing of F12 gene in 105 Italian patients with nC1-INH-HAE. Italian patients with FXII-HAE appear to come from the same common ancestor as those from other countries.

This nationwide survey of C1-INH-HAE provides for Italy a prevalence lower than in other European countries. We hyphotesize a disomogeneous geographical distribution of nC1-INH-HAE among European countries.

### O-22 Management of patients with hereditary angioedema with normal C1 inhibitor and without *F12* gene mutations

#### Konrad Bork^1^, Karin Wulff^2^, Guenther Witzke^1^, Jochen Hardt^3^

##### ^1^Department of Dermatology, University Mainz, Mainz, Germany; ^2^University Medicine, Ernst Moritz Arndt University, Greifswald, Germany; ^3^Department of Medical Psychology and Medical Sociology, University Mainz, Mainz, Germany

###### **Correspondence:** Konrad Bork (bork@hautklinik.klinik.uni-mainz.de)

*Allergy, Asthma & Clinical Immunology* 2017,** 13(Suppl 2)**:O-22

**Background:** Hereditary angioedema (HAE) with normal C1-inhibitor (C1-INH) may be associated with mutations in the gene coding for the coagulation factor XII (HAE-FXII) or with gene alterations still unknown (HAE-unknown). The management including the investigational use of various drugs in patients with HAE-unknown is reported.

**Methods:** Patients with HAE-unknown were treated for acute attacks or received long-term prophylaxis. Efficacy of drugs for acute attacks was determined by recording time to first relief and time to resolution of symptoms and compared to untreated attacks. Efficacy of drugs and other measures for long-term prophylaxis was determined by recording the number of attacks before and during treatment.

**Results:** In 11 patients a total of 28 treatments in an intensive care unit because of upper airway obstruction were reported. Four patients required intubation and two patients needed cricothyrotomia. All patients survived. Fourteen patients were treated for 52 acute attacks with C1-INH concentrate. Eleven patients reported an excellent efficacy for 42 treated attacks. Two of them observed a low efficacy in 8 further attacks. In two patients with tongue swelling C1-INH concentrate was not effective. Six patients were treated for 131 attacks with icatibant. All patients reported an excellent efficacy in 129 attacks. In two attacks further treatment was required. One patient was treated with fresh frozen plasma, however ineffectively. Concerning long-term prophylaxis, 11 women received desogestrel for a total of 33 years. Three of them became symptom-free. Desogestrel was not effective in 3 women and partially effective in 5 women. Seven patients received tranexamic acid for a total of 31 years. During this time period 4 patients were symptom-free. In two patients tranexamic acid was partially effective and in a further patient ineffective. Danazol was effective in 4 patients and ineffective in 2 patients. Two patients were treated with stanozolol for a total of 8 years. Stanozolol was partially effective in both patients.

**Conclusions:** In HAE with normal C1-INH and without mutations in the *F12* gene various treatment measures are completely or partially effective.

### O-23 Hereditary angioedema presenting in childhood is diagnosed in adulthood by non-paediatric physicians: findings from the Icatibant Outcome Survey

#### Hilary Longhurst^1^, Werner Aberer^2^, Laurence Bouillet^3^, Teresa Caballero^4^, Anette Bygum^5^, Anete S Grumach^6^, Christelle Pommie^7^, Irmgard Andresen^7^, Andrea Zanichelli^8^, Marcus Maurer^9^

##### ^1^Department of Immunology, Barts Health NHS Trust, London, UK; ^2^Department of Dermatology and Venereology, Medical University of Graz, Graz, Austria; ^3^National Reference Centre for Angioedema, Internal Medicine Department, CHU Grenoble Alpes, Université Grenoble Alpes, Grenoble, France; ^4^Allergy Department, Hospital La Paz Institute for Health Research (IdiPaz), Biomedical Research Network on Rare Diseases (CIBERER, U754), Madrid, Spain; ^5^Department of Dermatology and Allergy Centre, Odense University Hospital, Odense, Denmark; ^6^Faculty of Medicine ABC, São Paulo, Brazil; ^7^Shire, Zug, Switzerland; ^8^Department of Biomedical and Clinical Sciences Luigi Sacco, University of Milan, ASST Fatebenefratelli Sacco, Milan, Italy; ^9^Department of Dermatology and Allergy, Allergie-Centrum-Charité, Charité-Universitätsmedizin Berlin, Berlin, Germany

###### **Correspondence:** Hilary Longhurst (hilary.longhurst@bartshealth.nhs.uk)

*Allergy, Asthma & Clinical Immunology* 2017,** 13(Suppl 2)**:O-23

**Background:** Hereditary angioedema with C1 inhibitor deficiency (C1-INH-HAE) is a rare, often severe disease characterized by recurrent swellings of the skin and mucous membranes. A previous study enrolling 171 patients with C1-INH-HAE type I/II showed that although symptoms presented in childhood (median age 12 years), there was an 8.5-year delay from the time of symptom onset to diagnosis [1]. In this analysis, data from the Icatibant Outcome Survey (IOS) was used to determine which physicians are diagnosing C1-INH-HAE, to help identify where to focus awareness efforts.

**Methods:** The IOS is an international observational study monitoring the safety and effectiveness of icatibant for the treatment of angioedema attacks. All study participants provided written informed consent. Data were collected from 12 countries between July 2009 and January 2017.

**Results:** There were 683 patients (400 [58.6%] females) with C1-INH-HAE type I/II in this analysis. A family history of C1-INH-HAE was found in 500 (80.0%) of 625 patients with available data. Median (interquartile range) ages at first symptom onset and at diagnosis were 12.0 (5.0, 18.0) and 21.0 (13.2, 33.8) years, respectively. The majority of patients with available information on who made the diagnosis at baseline (n = 470) were diagnosed by a specialist (395, 83.9%) or a non-emergency room physician (76, 16.1%). Specialists who made the most diagnoses were allergologists (139, 29.5%), clinical immunologists (86, 18.3%), and dermatologists (76, 16.1%). Paediatricians and paediatrician-immunologists only diagnosed 12 (2.5%) patients and 10 (2.1%) patients, respectively.

**Conclusion:** Our findings confirm that despite the fact that C1-INH-HAE symptoms present in childhood or adolescence, paediatricians rarely diagnosed patients. Raising disease awareness among paediatricians may reduce the diagnostic delay, possibly allowing for more efficient referral of symptomatic patients to appropriate specialists.

*Trial Registration* NCT01034969

**Reference**

1. Zanichelli A, Magerl M, Longhurst H, Fabien V, Maurer M. Hereditary angioedema with C1 inhibitor deficiency: delay in diagnosis in Europe. Allergy Asthma Clin Immunol. 2013;9:29–32.

### O-24 How age, gender, and concomitant diseases influence the clinical course of hereditary angioedema

#### Inmaculada Martinez Saguer, Carmen Escuriola Ettingshausen, Zeynep Gutowski, Karin Andritschke, Richard Linde

##### Haemophilia Centre Rhine Main, Frankfurt-Mörfelden, Germany

###### **Correspondence:** Inmaculada Martinez Saguer (inmaculada.martinez@hzrm.de)

*Allergy, Asthma & Clinical Immunology* 2017,** 13(Suppl 2)**:O-24

**Background:** Hereditary angioedema due to C1-inhibitor deficiency (C1-INH-HAE) is a rare autosomal dominant disease, manifesting as potentially life-threatening oedema. Frequency and severity of these attacks vary at different stages of life and hormonal changes influence the clinical course of C1-INH-HAE. Several reports suggest a link with autoimmune conditions. The aim of this study is to analyse the potential influence of age and concomitant diseases on the clinical course of C1-INH-HAE.

**Materials and methods:** Adult patients with C1-INH-HAE, treated with human pasteurised and nanofiltered C1-esterase inhibitor concentrate and with a patient diary covering the previous 12 months, were selected and allocated into 2 age groups: 18–65 years and 65 years and older. Prodromal symptoms, attack triggers, location and severity and the occurrence of concomitant diseases were analysed retrospectively. Descriptive statistics were derived using SAS version 9.1.

**Results:** Patient diaries were available from 150 patients with C1-INH-HAE (53 men, 97 women), with a mean age (range) of 44.1 years (18–95), 134 patients being younger than 65 years (48 men, 86 women) and 16 being older (5 men, 11 women).

The most common concomitant diseases in younger patients were arterial hypertension and autoimmune thyroiditis (both in 22 of 134 patients), followed by allergic rhinitis (19 patients). This age group experienced 4840 attacks with 12% being severe and with pain being the most common first sign of an impending attack. In the small cohort of older patients, a similar pattern was seen: concomitant arterial hypertension (10 of 16 patients) was followed by autoimmune thyroiditis (5 patients), allergic rhinitis and hypercholesteremia (3 patients each). Only 4% of 818 attacks were severe and first signs manifested most commonly as erythema.

In men, the most common concomitant disease was arterial hypertension (15 of 53 patients), followed by allergic rhinitis and hypercholesteremia (9 patients each). Annual mean attack frequency (standard deviation [SD]) was 29.3 (45.2) with concomitant diseases and 26.6 (33.4) without. Women most commonly suffered from concomitant autoimmune thyroiditis (25 of 97 patients), followed by arterial hypertension (17 patients) and allergic rhinitis (13 patients). Mean attack frequency (SD) was 48.3 (55.2) with concomitant diseases and 28.6 (35.9) without.

**Conclusions:** Differences in the clinical course of C1-INH-HAE (attack frequency, severity, and first signs) were seen that could possibly be attributed to age or gender differences, or the influence of concomitant diseases. However, larger patient collectives will need to be studied to draw definitive conclusions.

### O-25 Comorbidities of patients with hereditary angioedema due to C1-inhibitor deficiency in Hungary

#### Kinga V. Kőhalmi, Noémi Andrási, Tamás Szilágyi, Nóra Veszeli, Lilian Varga, Henriette Farkas

##### Hungarian Angioedema Centre, 3rd Department of Internal Medicine, Semmelweis University, Budapest, 1125, Hungary

###### **Correspondence:** Kinga V. Kőhalmi (kinga.viktoria.kohalmi@gmail.com)

*Allergy, Asthma & Clinical Immunology* 2017,** 13(Suppl 2)**:O-25

**Background:** Limited data are available on the comorbidities in patients with hereditary angioedema due to C1-inhibitor deficiency (C1-INH-HAE). Our aim was to assess the prevalence of comorbidities and its effect on C1-INH-HAE among our patients.

**Materials and methods:** We surveyed 139 adult C1-INH-HAE patients (59 males, 80 females [mean age: 43.45, min: 19.04, max: 85.22 years [y]), according to the data of the Hungarian HAE Registry and the patient’s documentation assessed between 1996 and 2017. In our survey, we assessed the prevalence of hypertension, ischaemic heart disease (IHD), cerebrovascular diseases (CVD), diabetes mellitus (DM) and neoplasms according to different age groups (19–24, 25–34, 35–44, 45–54, 55–64, 65–74, >75 years) and compared these data to the Hungarian Central Statistical Office’s data (HCSO). We surveyed allergic diseases and appendectomy in C1-INH-HAE patients—without age groups—and we compared the data to the data of HCSO.

**Results:** In our adult patients with C1-INH-HAE, hypertension occurred significantly rarer in every age groups (19–24 years: p = 0.0469; 45–54 years: p = 0.0005; 55–64 years: p < 0.0001; 65–74 years: p = 0.0001) compared to HCSO data, except between 25–34 years (p = 0.0198) and >75 years (p < 0.0001). IHD appeared in significantly fewer cases in 45–54 years (p = 0.0002), 65–74 years (p < 0.0001), >75 years (p < 0.0001) old C1-INH-HAE patients compared to the Hungarian population. CVD had significantly lower prevalence in our C1-INH-HAE patients in the 45–54 years age group (p = 0.0337), while significantly higher prevalence in 35–44 years (p = 0.0002). DM’s prevalence was significantly lower in the 55–64 years (p = 0.0142), 65–74 years (p < 0.0001) and >75 years (p < 0.0001) C1-INH-HAE group compared to HCSO data. Regarding neoplasms, the results are controversial: there was significantly higher incidence in our C1-INH-HAE patients in the 25–34 years age group (p < 0.0001), but significantly lower prevalence in the >75 years (p = 0.0002) compared to HCSO’s data. These results are not influenced by danazol taking. Prevalence of allergic diseases (p = 0.0048) and of appendectomy (p < 0.0001) were significantly higher in the C1-INH-HAE group compared to the Hungarian population.

**Conclusions:** We can conclude that in high proportion of the age groups, hypertension, IHD and DM occur more rarely in the Hungarian C1-INH-HAE patients, therefore regarding these diseases, bradykinin may have a protective role in C1-INH-HAE. Regarding CVD and neoplasms, the results are controversial, while allergic diseases have a higher prevalence in C1-INH-HAE. Reasonably, appendectomy occurred with a higher prevalence in C1-INH-HAE patients according to the misdiagnosis of this rare disease.

### O-26 Launching a new website for the HAE International Nursing Organization

#### Iris Leibovich-Nassi^1^, Karin Andritschke^2^, Christine Symons^3^, John Dempster^4^, Avner Reshef^1^, Henriette Farkas^5^

##### ^1^Angioedema Research Center, Sheba Medical Center, 5265601, Tel-Hashomer, Israel; ^2^Rhein-Main Haemophilia Centre (HZRM), Moerfelden, 64546, Germany; ^3^Derriford Hospital, Plymouth, PL6 8DH, UK; ^4^Barts Health, Whitechapel, London, E1 1BB, UK; ^5^Hungarian Angioedema Center, 3rd Department of Internal Medicine, Semmelweis University, Budapest, 1125, Hungary

###### **Correspondence:** Iris Leibovich-Nassi (Iris.leibovich@sheba.health.gov.il)

*Allergy, Asthma & Clinical Immunology* 2017,** 13(Suppl 2)**:O-26

**Background:** The International Hereditary Angioedema (HAE) Nurses Organization (IHNO) is a global initiative, aims to help as many patients as possible to lead independent and productive lives, advance the quality of care in angioedema, and attain excellence in the nursing treatment, focusing on the patients and their families.

All participating nurses have an extensive experience with all forms of angioedema in specialty centers in their countries.The main purpose of the organization is to increase the impact of expert nurses on patient management and implementation of new treatment protocols, in line with global practice parameters, which balances technological developments, social needs and economic considerations.

**Methods:** A new internet website was constructed specifically for the IHNO.

The website will serve the following purposes:

1. Assist to develop consensus on global nursing treatment parameters.
2. Construct training programs directed at self-treatment with the available intravenous and subcutaneous medications.
3. Raise awareness of this rare genetic disease among health-care professionals.
4. Establish a global forum for discussions and exchange of ideas.
5. Publish presentations, posters and educational materials.
6. Academic and research publications in the field of angioedema nursing.
7. Help to organize meetings and conferences.
8. Open section for patient education and training.
9. Publish results of new studies and advancements in the field.
10. Communicate with the Pharma to encourage clinical studies and educational initiatives.

Nurses will be encouraged to register to the IHNO and receive information through the website.

**Conclusions:** The new website will foster better communication between the nurses, increase awareness of health-care providers and other medical professionals and help the IHNO to achieve its proclaimed goals.

### O-27 Patient’s educational therapeutic program “EDUCREAK”: fourth year assessment

#### Isabelle Boccon-Gibod, Anne Pagnier, Audrey Lehmann, Laurence Bouillet

##### CHUGA, Grenoble, France

###### **Correspondence:** Isabelle Boccon-Gibod (iboccon-gibod@chu-grenoble.fr)

*Allergy, Asthma & Clinical Immunology* 2017,** 13(Suppl 2)**:O-27

**Introduction:** The French National Reference Center (CREAK), licensed in 2006 within the Rare Diseases National Plan has set up a diagnostic and therapeutic network for HAE patients nationwide. Therapeutic management is a serious challenge. In France, the Health Authorities (HAS) and the National institute for health care prevention and education has published a methodology guide devoted to structuring a therapeutic education program for patients with chronic disease. HAE is a chronic disease with unpredictable and severe acute attacks that are potentially life threatening. Treatment has two main objectives: treat acute attacks and limit their occurrence in the short and long term. The acute treatment should be administered as soon as possible for better efficiency and patient safety. Self-administration should be encouraged for greater patient autonomy and safety (reducing delay to receive treatment injection). In 2012, the CREAK developed a patient’s educational therapeutic program “EDUCREAK”, licensed by Regional Agencies (ARS). This program allows patients and close family or partners to acquire skills for greater autonomy and safety in day to day disease management. At the end of the fourth year, this program has been assessed successfully by the French Health Care Agencies.

**Methods:** A standard detailed educative approach was applied, adapted to the specific problematic of HAE patients in relation to the severity of the disease and quality of life (AE-QoL and HAE-QoL score levels were assessed). In 2016, after 4 program’s years, questionnaires were sent to HAE patients and Health Care Professionals, and patients’ educational records have been analyzed. The program was developed in cooperation with patients: two referent HAE patients were included in this approach and were active in the program development.

**Results:** This specific program has included 250 individual educational visits or workshops per year. Four years after its institution, 93 patients have participated in this program. Almost all patients and Health Care Professional (HCP) Team have a positive perception of program usefulness for short and long term: disease awareness, better management of the attacks and improvement of quality of life.

**Conclusion:** EDUCREAK program set up those 4 last years for HAE patients has allowed a positive dynamic attitude for educational HCP team with a project focused on the HAE patients. It improved patients’ quality of life, autonomy and safety.

**References**

1. Boccon-Gibod I. Hereditary angioedema: treatment and educational therapeutic program. Paris: Presse Médicale; 2015.

2. Prior N, et al. Psychometric field study of hereditary angioedema quality of life questionnaire for adults: HAE-QoL. JACI Pract. 2016;4(3):464–73.e4

### O-28 Smartphone application to record data on attack and treatment patterns in patients with hereditary and acquired angioedema: findings from a Danish cohort

#### Kristian B. Kreiberg, Anette Bygum

##### HAE Centre Denmark, Department of Dermatology and Allergy Centre, Odense University Hospital, Odense, Denmark

###### **Correspondence:** Kristian B. Kreiberg (Kristian.buur.kreiberg2@rsyd.dk)

*Allergy, Asthma & Clinical Immunology* 2017,** 13(Suppl 2)**:O-28

**Background:** A minimal set of variables to be recorded for hereditary angioedema (HAE) attacks has been defined [1]. However, reporting even these minimal requirements appears difficult for some patients, often resulting in missing data or retrospective reporting. We hypothesize that utilizing a user-friendly reporting platform in the form of a smartphone application, will increase compliance and make real time registration easier. The primary purpose is to investigate real-life data on attack duration and attack frequency.

**Materials and methods:** The study is a retrospective observational study performed at HAE Centre Denmark at Odense University Hospital. Data from the first three years of utilizing the application was reviewed (September 2013–2016). A total of 69 patients had utilized the application, all provided written informed consent to access data from the application and medical records, and of these 44 fulfilled the inclusion criteria: a verified diagnosis of HAE or acquired angioedema (AAE) and at least 8 months of consecutive recording. Patients were instructed to make entries when attacks commenced, when treatment was initiated, and when attacks resolved.

**Results:** Forty-two patients had HAE, two had AAE. The 44 patients recorded a total of 2156 attacks. Based on average attack frequency, patients were classified as having a mild (<1 attack/month), moderate (between 1 and 3 attacks/month) or severe (>3 attacks/month) disease activity. Mean attack duration in mild, moderate and severe groups were 23.0 h (±24.2), 22.0 h (±20.9) and 16.1 h (±15.1) respectively. Time from attack onset to treatment in the mild, moderate, and severe groups were 6.1 h (±12.9), 4.4 h (±12.0), and 1.5 h (±5.8) respectively. In the mild, moderate and severe groups 72, 87 and 100% of patients self-administered on-demand treatment respectively. Long-term prophylaxis (LTP) was utilized by 17, 40, and 64% of patients in the mild, moderate and severe groups respectively. In general men with HAE had an attack rate 0.61 times that of women with HAE [P < 0.001, 95% CI (0.51; 0.72)], when correcting for age at disease onset. For every year symptom onset in HAE was delayed, attack rate decreased by a factor of 0.97 [P < 0.001, 95% CI (0.95; 0.98)], when correcting for gender.

**Conclusions:** Attack duration was lower among patients with severe disease activity, possibly due to proportionately more patients utilizing LTP, better skills in self-administration, and early treatment. Females and patients who presented symptoms early in life were found to have increased attack rates.

**Reference**

1. Cicardi M, Bork K, Caballero T, Craig T, Li HH, Longhurst H, Reshef A, Zuraw B on behalf of HAWK (Hereditary Angioedema International Working Group). Evidence-based recommendations for the therapeutic management of angioedema owing to hereditary C1 inhibitor deficiency: consensus report of an International Working Group. Allergy. 2012;67:147–57.

### O-29 Hereditary angioedema in Latin America: are we improving?

#### Anete S. Grumach^1^, Sandra Nieto^2,3^, Raquel Martins^4^, Renata Martins^4^, Alejandra Menendez^5^, Solange O. R. Valle^6^, Margarita Olivares^7^, Maria E. Hernandez-Landeros^8^, Elma Nievas^9^, Natalia Fili^10^, Olga M. Barrera^11^, René Bailleau^12^, Ana Maria Gallardo-Olivos^13^, Masumi Grau^14^, Julian Rodriguez-Galindo^15^, Marlon J. O. Carabantes^16^, Edison Zapata-Venegas^17^, Mario Martinez Alfonso^18^, Maria Rosario-Grauert^19^, Manuel Ratti^20^, Daniel Vaszquez^21^, Dario Josviack^22^, Luis Fernando Landivar-Salinas^23^, Oscar M. E. Calderón-Llosa^24^, Rolando Campilay-Sarmiento^25^, Pablo Raby^26^, Jose Fabiani^27^

##### ^1^Clinical Immunology, ABC School of Medicine, Santo Andre, SP, Brazil; ^2^National Institute of Pediatrics, Mexico City, Mexico; ^3^Mexican Association of Patients with Hereditary Angioedema, Mexico, Mexico; ^4^Brazilian Association of Patients with Hereditary Angioedema (ABRANGHE), São Paulo, Brazil; ^5^Association of Patients with Hereditary Angioedema from Argentina, Buenos Aires, Argentina; ^6^Federal University of Rio de Janeiro, RJ, Brazil; ^7^Group of Clinical and Experimental Allergology (GACE), Medellín, Colombia; ^8^Medicina Clinica del Este “Rosa Rangel”, Xalapa, Veracruz, México; ^9^Allergy Clinic, Buenos Aires, Argentina; ^10^Allergist, Salta Capital, Salta, Argentina; ^11^Jefa de la Clínica de Alergias e Inmunología del Instituto de Neumologia y Alergias del Hospital San Fernando, Panamá, Panama; ^12^Clinics of Allergy, Buenos Aires, Argentina; ^13^Hospital Barros Luco Trudeau, Santiago, Chile; ^14^Hospital El Pino, University of Santiago, Santiago, Chile; ^15^San Pablo Clinic, Lima, Peru; ^16^Allergy and Clinical Immunology, Hospital Nacional Rosales,San Salvador, El Salvador; ^17^Hospital Quito N.1, National Police of Equador, Quito, Equador; ^18^Clinics of Allergy, San Jose, Costa Rica; ^19^Allergy Clinic, Montevideo, Uruguay; ^20^Allergy Clinic, Assuncion, Paraguay; ^21^Allergy Sevice “Clinica Estrada”, Buenos Aires, Argentina; ^22^Institute of Respiratory Medicine, Rafaela, Santa Fe, Argentina; ^23^Clinical Allergist, Santa Cruz, Bolivia; ^24^Medical Center San Sebastian, Piura, Peru; ^25^Hospital Barros Luco Trudeau, Santiago, Chile; ^26^Hospital El Pino, University of Santiago, Santiago, Chile; ^27^Researcher in Hereditary Angioedema at the Argentinian Institute of Allergy and Founder of Latin American Association of Hereditary Angioedema (ALAeh), Buenos Aires, Argentina

###### **Correspondence:** Anete S. Grumach (asgrumach@gmail.com)

*Allergy, Asthma & Clinical Immunology* 2017,** 13(Suppl 2)**:O-29

**Background:** Prevalence of Hereditary Angioedema is approximately 1:50,000 inhabitants. Latin America population in estimated as 635,000,000, so we would expect 12,700 HAE patients. In 2013, less than 5% of the patients had been identified. Misdiagnosis, difficult access to biochemical tests and no access to therapy have been the routine in developing countries. We organized a meeting, supported by the Latin American Association for HAE (ALAeh), in order to update our situation.

**Methods:** Physicians interested on HAE and its development in their country were invited to present the identified patients.

**Results:** Thirteen countries reported 1603 cases (2F:1M). Excluding Argentina, Brazil and Mexico, 144 patients were diagnosed in the other 10 countries in comparison with 35 previously reported in 2013, out of 6 countries. Mean age at diagnosis is 29 years old although most reported initial symptoms less than 10 years of age. Clinical symptoms were distributed as previously reported in other populations but upper airways obstruction occurred in 1/3 of the patients. The accomplishment in Chile was extraordinary with a program that improved from 4 to 51 confirmed HAE patients in 4 years (1:353,000). Ten out of 13 countries do not have access to on demand therapy.

**Conclusions:** Latin America doubled the number of HAE patients identified in the last 4 years. Moreover, most countries improved the diagnosis although on demand therapy is not accessible. The registry has to be established in order to have accurate data from the Continent.

### O-30 Can routine prophylactic subcutaneous C1-inhibitor [C1-INH(SC)] alleviate psychological and physical disabilities caused by HAE? Findings from the COMPACT study (NCT01912456)

#### William R. Lumry^1^, Timothy Craig^2^, Marco Cicardi^3^, Bruce L. Zuraw^4^, Hilary Longhurst^5^, Henriette Farkas^6^, Henrike Feuersenger^7^, Douglas J. Watson^7^, Thomas Machnig^7^, on behalf of the Investigators of the COMPACT (NCT01912456) study

##### ^1^AARA Research Center, Dallas, TX, 75231, USA; ^2^Department of Medicine and Pediatrics, Penn State University Allergy, Immunology and Respiratory Research, Hershey, PA, 17033, USA; ^3^Department Biomedical and Clinical Sciences Luigi Sacco University of Milan, ASST Fatebenefratelli Sacco, 20157, Milano, Italy; ^4^Department of US HAEA Angioedema Center, UC San Diego, La Jolla, CA, 92193, USA; ^5^Department of Immunology, Barts Health NHS Trust, London, E1 1RD, UK; ^6^Department of Internal Medicine, Hungarian Angioedema Center, Semmelweis University, Budapest, 1085, Hungary; ^7^CSL Behring GmbH, 35002, Marburg, Germany

###### **Correspondence:** Thomas Machnig (thomas.machnig@cslbehring.com)

*Allergy, Asthma & Clinical Immunology* 2017,** 13(Suppl 2)**:O-30

**Background:** The painful and life-threatening hereditary angioedema (HAE) attacks are associated with oedema leading to disability and impairment in daily activities [1]. This is very stressful for patients and their families and may be associated with anxiety and depression [2]. Here, findings from the subject-reported outcome measures in the Phase 3 COMPACT study are reported.

**Materials and methods:** In this study, 90 subjects with HAE were randomized to 1 of the 4 treatment sequences to receive 40 or 60 IU/kg C1-INH(SC) twice-weekly for 16 weeks, preceded or followed by placebo for 16 weeks. Analyses were carried out in those subjects who provided at least one subject–reported outcome assessment (Quality-of-Life [QoL] population). The exploratory analyses evaluated subject–reported outcome measures at screening and post-treatment. The measure, work productivity and activity impairment (WPAI) was assessed after 5, 8, 11, and 14 weeks of each treatment period while the Hospital Anxiety and Depression Scale (HADS) and Treatment Satisfaction Questionnaire for Medication (TSQM) measures were assessed after 5 and 14 weeks of each treatment period. The absolute mean/median values at each assessment during the active and placebo periods and the within-subject differences for each dose (and both doses combined) of C1-INH(SC) versus placebo were calculated for the individual domains of each measure at week 14.

**Results:** *Absenteeism*, *Work Productivity Loss* (WPAI) and *Overall Satisfaction* (TSQM) domain scores reflected some impairment at baseline. The baseline (HADS) *Anxiety* scores were in the high normal range. Treatment with C1-INH(SC) (both doses combined; n = 58) demonstrated treatment differences compared to placebo (mean [95% CI]) on the *Anxiety* HADS domain (−1.05 [−1.79, −0.31]) and the WPAI domains of *Presenteeism* (−15.86 [−25.21, −6.52]), *Work Productivity Loss* (−19.97, [−30.84, −9.10]), and *Activity Impairment* (−19.83 [−27.28, −11.88]). Possible treatment effects of both doses of C1-INH(SC) compared to placebo after 14 weeks were also observed for TSQM *Effectiveness* and *Overall Satisfaction domain scores*.

**Conclusions:** Results of these exploratory subject reported outcomes suggest that prophylactic C1–INH(SC) treatment may alleviate some of the psychological and physical impairments due to HAE.

**References**

1. Lumry WR, Miller DP, Newcomer S, Fitts D, Dayno J. Quality of life in patients with hereditary angioedema receiving therapy for routine prevention of attacks. Allergy Asthma Proc. 2014;35:371–6.

2. Quality-of-life issues with HAE. http://www.allabouthae.com/professional/quality-of-life-issues-hae.aspx. Accessed 2 Mar 2017.

### O-31 Health related quality of life in adult patients with C1-INH-HAE

#### Donatella Lamacchia^1^, Adriana Hernanz^2^, Ana Alvez^2^, Mariana Lluncor^2^, Maria Pedrosa^2^, Rosario Cabañas^2^, Nieves Prior^2^, Teresa Caballero^2,3^

##### ^1^Department of Translational Medical Sciences, Center of Basic and Clinical Immunology Research, University of Naples Federico II, Naples, Italy; ^2^Department of Allergy, Hospital La Paz Institute for Health Research (IdiPaz), Madrid, Spain; ^3^Biomedical Research Network on Rare Diseases (CIBERER, U754), Madrid, Spain

###### **Correspondence:** Donatella Lamacchia (donatella.lamacchia@gmail.com)

*Allergy, Asthma & Clinical Immunology* 2017,** 13(Suppl 2)**:O-31

**Background:** C1-INH-HAE is a rare disease with impairment in health related quality of life (HRQoL) as measured by generic HRQoL questionnaires (SF-36, E5QD). We aimed to study HRQoL in adult patients with C1-INH-HAE by means of generic (E5QD), symptom specific (AE-QoL) and disease-specific (HAE-QoL) questionnaires.

**Methods:** The study was approved by Hospital La Paz Ethics Committee (PI-2297). Spanish patients ≥18 year. with C1-INH-HAE were included. C1-INH-HAE activity was measured by HAE-AS (range 0–30). HRQoL was measured by E5QD (range 0–1), HAE-QoL (range 25–135) and AE-QoL (range (0–100). Data were entered into an excel database and statistical analysis was performed using SPSS v. 20 for IOS.

**Results:** 58 patients (35 females, 60.3%; 23 males, 39.7%) were included. Mean age was 45.6 ± 14.1 year. Mean HAE-AS was 7.0 ± 4.6. Mean HAE-QoL score was 100.8 ± 25.8, whereas mean adjusted AE-QoL score was 33.6 ± 23.4. Mean E5QD value was 0.927 ± 0.098. There was a significant negative correlation between HAE-QoL and AE-QoL scores (−0.897, p < 0.0001). The most affected dimension in HAE-QoL was “perceived control over disease” and the least affected “treatment difficulties” (with a 39 and 17% mean decrease with respect to dimension maximum score). The most affected dimension in AE-QoL was “fear/shame” (38.9 ± 26.3) and the least affected “functioning” (38.9 ± 28.4).

There was a positive weak correlation between age and HAE-QoL score (0.2965) (p = 0.024) and negative weak correlation between age and AE-QoL score (−0.2399) (p = 0.072).

HRQoL was more impaired in females than males. HAE-QoL scores (mean ± SD) were higher in males (107.8 ± 20.3) than females (96.2 ± 28.2), whereas AE-QoL scores (mean ± SD) were lower in males (27.5 ± 20.6) vs females (37.5 ± 24.5), but without significant differences. When studying HAE-QoL and AE-QoL domains the only significant differences between males and females were regarding “emotional role/social functioning” (p = 0.0397) and “mental health” (p = 0.0462) dimensions in HAE-QoL.

C1-INH-HAE disease activity (HAE-AS score) was negatively correlated with HAE-QoL (−0.704, p < 0.001) and E5QD (−0.622, p < 0.001) scores and positively correlated with AE-QoL score (0.736, p < 0.001).

**Conclusions:** HAE-QoL and AE-QoL scores show a high and significant negative correlation. Both HAE-QoL and AE-QoL had a moderate correlation with C1-INH-HAE activity as measured by HAE-AS. Perceived control over disease was the most affected dimension in HAE-QoL.

### O-32 Study of health-related quality of life and disease activity in adults with HAE in Sweden

#### Patrik Nordenfelt^1,2^, Mats Nilsson^3^, Anders Lindfors^4^, Carl-Fredrik Wahlgren^5,6^, Janne Björkander^1,3^

##### ^1^Department of Clinical and Experimental Medicine, University of Linköping, Linköping, Sweden; ^2^Department of Internal Medicine, County Hospital Ryhov, Jönköping, Sweden; ^3^Futurum-Academy for Health and Care, Region Jönköping County, Jönköping, Sweden; ^4^Department of Pediatrics, Astrid Lindgren Children’s Hospital, Karolinska University Hospital, Stockholm, Sweden; ^5^Dermatology Unit, Department of Medicine Solna, Karolinska Institutet, Stockholm, Sweden; ^6^Department of Dermatology, Karolinska University Hospital, Stockholm, Sweden

###### **Correspondence:** Patrik Nordenfelt (patrik.nordenfelt@telia.com)

*Allergy, Asthma & Clinical Immunology* 2017,** 13(Suppl 2)**:O-32

**Background:** Hereditary angioedema (HAE) is known to affect health-related quality of life (HR-QoL) [1, 2]. To better understand the impact on HR-QoL of diseases with recurrent angioedema, like HAE, two instruments, Angioedema Quality of Life (AE-QoL) and Angioedema Activity Score (AAS) have been developed [3, 4].

**Aim:** To better understand the impact HAE has on HR-QoL in Swedish adults, we used the new instruments AE-QoL and AAS in combination with the generic HR-QoL instruments EuroQoL 5 Dimension 5 level (EQ-5D-5L) and RAND-36.

**Methods:** All identified allegeable adults with HAE received a questionnaire with RAND-36, EQ-5D-5L, AE-QoL and AAS instruments. AAS was filled in during 4 weeks. A questionnaire about medication and sick leave was completed.

RAND-36 gives results in 9 dimensions from 0 to 100, where 100 is the most favourable result [5]. EQ-5D-5L gives a value ranging from sometimes negative values to 1, where 1 is the best [6]. AE-QoL produces results 0–100 in four dimensions, where 0 is the most favourable. The AAS increases with the disease activity.

**Results:** Sixty-four adults (26 males and 38 females) between 18 and 91 years responded. Response rate was 48%. Females had significantly lower HR-QoL than males measured with the RAND-36 instrument on two dimensions; general health, scoring 50 (females) vs 75 (males), and energy/fatigue scoring 50 (females) vs 70 (males), p < 0.05.

Sick leaves due to HAE were reported from 36 patients during 4 weeks, 9 had in median 2 (0.25–8) sick days and 27 had no sick leave. Patients with sick leave reported impaired HR-QoL with EQ-5D-5L = 0.71 (0.1–1.0) vs 0.88 (0.55–1.0) in patients with no sick leave. The corresponding values for patients with sick leave or not were for AE-QoL = 53.7 (27.9–69.1) vs 25.7 (0–58.8) and the pain dimension of RAND-36 = 50 (12.5–57.5) vs 83.8 (32.5–100), p < 0.05.

Patients who reported any AAS had significantly more impaired HR-QoL on all dimensions. The Spearman rank order correlation between AAS and EQ-5D-5L was *r*(46) = −0.39, p < 0.05; between AAS and the dimensions of AE-QoL, it ranged between *r*(46) = 0.44 and *r*(46) = 0.75, p < 0.05. For AAS and the dimensions of RAND-36, the correlations varied between *r*(46) = −0.26 and *r*(46) = −0.67, p < 0.05, except for physical function.

**Conclusion:** Swedish females with HAE were more affected in their HR-QoL than males. Impairment in HR-QoL correlated with sick leave due to HAE and with disease activity, measured with AAS.

**References**

1. Caballero T, Aygoren-Pursun E, Bygum A, Beusterien K, Hautamaki E, Sisic Z, et al. The humanistic burden of hereditary angioedema: results from the burden of illness study in Europe. Allergy Asthma Proc. 2014;35:47–53.

2. Nordenfelt P, Dawson S, Wahlgren CF, Lindfors A, Mallbris L, Bjorkander J. Quantifying the burden of disease and perceived health state in patients with hereditary angioedema in Sweden. Allergy Asthma Proc. 2014;35:185–90.

3. Weller K, Groffik A, Magerl M, Tohme N, Martus P, Krause K, et al. Development and construct validation of the angioedema quality of life questionnaire. Allergy. 2012;67(10):1289–98.

4. Weller K, Groffik A, Magerl M, Tohme N, Martus P, Krause K, et al. Development, validation, and initial results of the angioedema activity score. Allergy. 2013;68:1185–92.

5. Hays RD, Morales LS. The RAND-36 measure of health-related quality of life. Ann Med. 2001;33:350–7.

6. Herdman M, Gudex C, Lloyd A, Janssen M, Kind P, Parkin D, et al. Development and preliminary testing of the new five-level version of EQ-5D (EQ-5D-5L). Qual Life Res. 2011;20:1727–36.

## Poster presentations

### P-1 *SERPING1* gene typing in the era of next-generation sequencing (NGS)

#### Gedeon Loules^1^, Maria Zamanakou^1^, Dorottya Csuka^2^, Matthaios Speletas^3^, Fotis Psarros^4^, Faidra Parsopoulou^3^, Markus Magerl^5^, Marcus Maurer^5^, Henriette Farkas^2^, Anastasios E. Germenis^3^

##### ^1^CeMIA SA, Larissa, Greece; ^2^Hungarian Angioedema Center, 3rd Department of Internal Medicine, Semmelweis University, Budapest, Hungary; ^3^Department of Immunology & Histocompatibility, School of Health Sciences, Faculty of Medicine, University of Thessaly, Larissa, Greece; ^4^Department of Allergology, Navy Hospital, Athens, Greece; ^5^Department of Dermatology and Allergy, Charité-Universitätsmedizin Berlin, Berlin, Germany

###### **Correspondence:** Anastasios E. Germenis (agermen@med.uth.gr)

*Allergy, Asthma & Clinical Immunology* 2017,** 13(Suppl 2)**:P-1

**Background:** With every passing day, genotyping of subjects who may suffer from hereditary angioedema (HAE) becomes more indispensable in the clinical practice. The conventional approach to the detection of disease-causing *SERPING1* variants is time-consuming and fraught with many pitfalls.

**Materials and methods:** A NGS custom platform (NGS-HAE) was designed using the Ion AmpliSeq ThermoFisher Scientific Designer^®^, in order to analyze *SERPING1* in its full length (all exons, introns, promoter, 5′- and 3′-untranslated regions-UTRs). A 100% coverage of all translated regions and UTRs was achieved with missing areas located only in intronic regions (overall coverage ≈ 83%). An intermediate version of the platform analyzing up to 825 amplicons was also tested aiming to examine its ability to detect copy number variations (CNVs). Annotation of variants and CNVs analysis was performed by variantCaller v5.04.0^®^ and Ion Reporter software v.5.2^®^ (Thermo Scientific). Ninety-three conventionally typed DNA samples from HAE patients carrying different *SERPING1* variants (52 single nucleotide variants—SNVs, 28 indels, 13 large defects) were blindly analyzed for the forward validation of NGS-HAE, with reverse validation still pending.

**Results:** NGS-HAE results were concordant with conventional *SERPING1* typing in 88/93 cases (51/52 SNVs, 26/28 indels and 11/13 large defects). Out of the five discrepancies observed, the conventional approach had miss-assigned two substitutions as large defects, while the NGS-HAE analysis algorithm was not able to identify one small deletion and two nucleotide substitutions. Hotspot regions were introduced in the variantCaller analysis for the detection of these three unidentified variants, elevating the concordance rate in regard with SNVs detection to 100%. Moreover, in 12/28 indels nomenclature differences were reported, with Ion Reporter assignment verified by Sanger reanalysis. Loss of heterozygosity of intronic mutations was observed in 6/13 large defects, indicating the presence of large deletions. CNV analysis resulted in the identification of CNVs of altered extend in all samples bearing large defects as well as false positive CNVs in samples bearing SNVs.

**Conclusions:** Our NGS custom platform represents a time- and cost-efficient screening approach for *SERPING1* typing that is valid for the detection of the vast majority of disease-associated SNVs. Given that conventional typing methods and workflows are highly dependent on the users’ experience and knowledge, the NGS-HAE could be a useful initial approach for detecting HAE-causing *SERPING1* variants.

### P-2 Hereditary angioedema: report from the Czech registry

#### Roman Hakl^1^, Pavel Kuklínek^1^, Irena Krčmová^2^, Jana Hanzlíková^3^, Martina Vachová^3^, Radana Zachová^4^, Marta Sobotková^4^, Jana Strenková^5^, Jiří Litzman^1^

##### ^1^Department of Clinical Immunology and Allergology, St. Anne’s University Hospital in Brno, Masaryk University, Brno, Czech Republic; ^2^Department of Clinical Immunology and Allergology, University Hospital in Hradec Králové, Charles University, Hradec Kralove, Czech Republic; ^3^Department of Immunology and Allergology, University Hospital in Plzeň, Charles University, Plzeň, Czech Republic; ^4^Department of Immunology, University Hospital in Motol, Charles University, Motol, Czech Republic; ^5^Institute of Biostatistics and Analyses at the Faculty of Medicine and the Faculty of Science of the Masaryk University, Brno, Czech Republic

###### **Correspondence:** Roman Hakl (roman.hakl@fnusa.cz)

*Allergy, Asthma & Clinical Immunology* 2017,** 13(Suppl 2)**:P-2

**Background:** Hereditary angioedema (HAE) is a rare, autosomal dominant disorder characterized by recurrent attacks of subcutaneous or sub-mucosal oedema. Symptoms are extremely variable in frequency, localization and severity. Laryngeal attacks are potentially life threatening, the patients are at risk of suffocation during the attack.

**Methods:** The goal of this study was to analyse HAE attacks in the Czech Republic (CR) between March 2012 to December 2016. Data were collected from the Czech National Registry of Primary Immunodeficiencies.

**Results:** The registry contains data of 150 HAE patients (females 81, males: 69; HAE type I 86.7%, HAE type II 13.3%), showing HAE prevalence 1.42 per 100,000 inhabitants. 2515 attacks in 119 (females: 65, males: 54; HAE type I 86.6%, HAE type II 13.4%) patients were recorded. The potential triggering factors for HAE attacks included stress (9.2%), trauma (7.1%) and infection (4.7%). However, in most attacks triggering factor was not identified (71.9%). The most frequent were abdominal attacks (54.7%) followed by peripheral oedema (33.8%). Laryngeal oedema was presented in 10.6% of attacks. 20.9% attacks were combined. Prodromal symptoms (most often erythema marginatum, weakness or nausea) were reported by 13.6% of attacks. 2060 attacks (81.9%) were actively treated (65.8% icatibant, 21.5% recombinant C1-INH, 5.9% plasma derived, highly purified, nanofiltered C1 inhibitor (pnfC1-INH), 0.5% plasma derived, nanofiltered pdC1-INH (nfC1-INH), 0.1% fresh frozen plasma, 4.2% increase in androgens dosage, 1.8% increase in tranexamic acid dosage). Treatment had to be repeated in 312 attacks (15.1%). Hospitalization was necessary in 25 attacks (1.2%). Emergency medical service (EMS) was used in 11 attacks (0.5%).

**Conclusions:** The analysis of HAE attacks gives further insight into their course. Our results show marked clinical variability in HAE patients. The fact that in more than 15% of attacks required repeated treatment of single attack shows that although various therapeutic approaches are available, it is still difficult to choose the best therapeutic approach for a concrete patient.

### P-3 Radiation as a trigger of attacks in a misdiagnosed patient with hereditary angioedema and Hodgkin’s disease

#### Maria Palasopoulou, Gerasimina Tsinti, Anastasios E. Germenis, Matthaios Speletas

##### Department of Immunology & Histocompatibility, School of Health Sciences, Faculty of Medicine, University of Thessaly, Larissa, Greece

###### **Correspondence:** Anastasios E. Germenis (agermen@med.uth.gr)

*Allergy, Asthma & Clinical Immunology* 2017,** 13(Suppl 2)**:P-3

**Background:** Understanding triggers associated with angioedema attacks can help towards a better recognition of impending attacks and the implementation of preventive behavioral or treatment measures.

**Case presentation:** A 34-year-old woman presented to our Outpatient Clinic on December 2012 with a typical history of recurrent angioedema attacks of various severity since her age of 14 years. A mean frequency of 2.5 attacks per year was reported with involvement of her hands, face, neck, tongue (twice) and abdomen. Stress and infections had been recognized as triggering factors of these attacks. The clinical picture had been attributed to food allergy and, although ineffective, corticosteroids were administered systematically, continuously at least for the last 10 years. Type I hereditary angioedema due to C1-inhibitor deficiency (C1-INH-HAE) was diagnosed after the measurement of C4, antigenic and functional C1-inhibitor levels and the diagnosis was confirmed by *SERPING1* genotyping that revealed a novel, de novo missense mutation (c.239C>G, p.A80G). On demand treatment with human C1-esterase inhibitor (Berinert^®^) or icatibant (Firasyr^®^) resulted in successful control of the attacks and great improvement of the patient’s quality of life. A second pregnancy, 8 months after diagnosis, aggravated the frequency and the severity of attacks but terminated normally by on demand administration of human C1-esterase inhibitor. Twenty-one months after the diagnosis of angioedema, the patient suffered from a cervical lymphadenopathy and a diagnosis of Hodgkin’s lymphoma was performed. The lymphoma was proved refractory to an ABVD regimen (doxorubicin, bleomycin, vinblastine, and dacarbazine) administered initially along with radiotherapy due to local disease as well as to the administration of nivolumab (PD-1 blocker) that followed. Lymphoma diagnosis was followed by exacerbation of angioedema. Especially, radiotherapy sessions were triggering severe edema attacks at the sites of radiation, a fact that necessitated the prophylactic administration of human C1-esterase inhibitor before each session. Furthermore, the administration of nivolumab resulted also in exacerbation of angioedema attacks, especially in abdomen.

**Conclusion:** Coincidence of C1-INH-HAE with Hodgkin’s lymphoma has not been reported as yet. However, the emergence of lymphoma in this patient cannot be considered unrelated to such a long-standing administration of corticosteroids. Radiation and nivolumab treatment are reported as triggering factors of angioedema attacks for a first time in the literature.

**Consent to publish:** Written consent to publish was obtained from the patient.

### P-4 The *KLKB1*-Ser143Asn polymorphism: a new genetic biomarker predicting the age of disease onset in patients with hereditary angioedema due to C1-INH deficiency (C1-INH-HAE)

#### Panagiota Gianni^1^, Gedeon Loules^2^, Maria Zamanakou^2^, Maria Kompoti^1^, Dorottya Csuka^3^, Fotis Psarros^4^, Markus Magerl^5^, Dimitru Moldovan^6^, Marcus Maurer^5^, Matthaios Speletas^1^, Henriette Farkas^3^, Anastasios E. Germenis^1^

##### ^1^Department of Immunology & Histocompatibility, University of Thessaly, School of Health Sciences, Faculty of Medicine, Larissa, Greece; ^2^CeMIA SA, Larissa, Greece; ^3^Hungarian Angioedema Center, 3rd Department of Internal Medicine, Semmelweis University, Budapest, Hungary; ^4^Department of Allergology, Navy Hospital, Athens, Greece; ^5^Department of Dermatology and Allergy, Charité-Universitätsmedizin Berlin, Berlin, Germany; ^6^Allergy and Lung Function Office, University of Medicine and Pharmacy, Tirgu Mures, Romania

###### **Correspondence:** Anastasios E. Germenis (agermen@med.uth.gr)

*Allergy, Asthma & Clinical Immunology* 2017,** 13(Suppl 2)**:P-4

**Background:** Our previous studies have shown that, in patients with C1-INH-HAE, *SERPING1* mutations and the carriage of the *F12*-46C/T polymorphism are independently associated with the age of disease onset [1, 2]. Plasma kallikrein is involved in the cascade of bradykinin production and activation of factor XII, and the G allele of its S143N (rs3733402) variant has been recently associated with less plasma kallikrein activity [3]. Our aim was to investigate the possible contribution of the *KLKB1*-Ser143Asn polymorphism (rs3733402) in C1-INH-HAE clinical phenotype.

**Materials and methods:** 218 type I C1-INH-HAE patients (age at analysis 37.03 ± 18 years) from 100 European families (41 Hungarian, 32 Greek, 16 German, 11 Romanian) were studied. All patients were previously genotyped for *SERPING1* and *F12* variants. The *KLKB1*-Ser143Asn polymorphism was detected by direct sequencing of exon 5. The possible associations of Ser143Asn polymorphism with the clinical features, combined or not with the carriage of *SERPING1* mutations and/or the *F12*-46C/T polymorphism, were explored by the use of generalized estimating equations (GEE), an extension of the generalized linear model that accounts for the within-subject correlations.

**Results:** 117 patients were genotyped as heterozygous, 57 as homozygous for the A allele and 44 as homozygous for the G allele (allele frequency: G = 47%, A = 53%). In a GEE linear regression model with age at disease onset as dependent variable and adjusted for carriage of the *F12*-46C/T polymorphism, the presence of the G allele of *KLKB1*-Ser143Asn polymorphism was significantly associated with a delayed disease onset by 4.4 years compared with patients carrying the A allele (p < 0.011).

**Conclusions:** The presence of the G allele of *KLKB1*-Ser143Asn polymorphism is an independent genetic factor strongly correlated with a delayed disease onset in patients with type I C1-INH-HAE.

**References**

1. Speletas M, Szilagyi A, Psarros F, Moldovan D, Magerl M, Kompoti M, et al. Hereditary angioedema: molecular and clinical differences among European populations. J Allergy Clin Immunol. 2015;135:570–3.

2. Speletas M, Szilágyi Á, Csuka D, Koutsostathis N, Psarros F, Moldovan D, et al. *F12*-46C/T polymorphism as modifier of the clinical phenotype of hereditary angioedema. Allergy. 2015;70:1661–4.

3. Gittleman HR, Merkulova A, Alhalabi O, Stavrou EX, Veigl ML, Barnholtz-Sloan JS, Schmaier AH. A cross-sectional study of KLKB1 and PRCP polymorphisms in patient samples with cardiovascular disease. Front Med. 2016;3:17.

### P-5 Acquired angioedema due to C1INH deficiency. Description of 12 cases and screening for free and complexed anti-C1INH autoantibodies

#### Albert López-Lera, Sofia Garrido, Margarita López-Trascasa

##### Unidad de Inmunología, CIBERER U754, Hospital Universitario La Paz, Madrid, Spain

###### **Correspondence:** Albert López-Lera (alberlole@gmail.com)

*Allergy, Asthma & Clinical Immunology* 2017,** 13(Suppl 2)**:P-5

**Introduction:** Angioedema due to acquired deficiency of C1Inhibitor (C1INH) (AAE) is an extra-rare disorder leading to bradykinin-triggered angioedema episodes. AAE is frequently associated to B cell proliferation and the development of haemotological diseases, while inactivating anti-C1INH autoantibodies may be detected (AAE type II) or not (AAE type I) in the patients’ plasma. Whether detectable or not, these autoantibodies are thought to drive AAE physiopathology by turning C1INH from an inhibitor into a substrate of plasma proteases FXII, FXI, Kallikrein (KK), C1s, C1r and Tissue Plasminogen Activator, although experimental evidence of this mechanism is only available for C1s. Protease deregulation in AAE exhausts plasma C1INH and frequently leads to severe complement C1q consumption, both features being diagnostic landmarks of AAE.

**Objective**: To analyze the clinical and biochemical characteristics of 12 AAE cases and the negative effects of anti-C1INH autoantibodies on C1INH conformation, residual inhibitory activity on KK and C1s, and contact system activation status in plasma.

**Results**: At the time of diagnosis, 10/12 of patients had low C1INH levels, autoantibodies were initially detected in 6/12 and C1q was undetectable in 9/12. Residual C1INH inhibitory activity was extremely variable and did not correlate with the observed complement consumption. Purified IgG from one patient dose-dependently induced C1INH cleavage in the presence of KK. Moreover, among autoantibody-negative patients and by using a novel in-house ELISA assay, we for the first time detected circulating C1INH:IgG or C1INH:IgM in 5/6 otherwise autoantibody-negative and in all positive cases in our series.

**Conclusion**: The detection of plasma C1INH:Ig complexes in autoantibody-negative patients is clinically relevant and indicates that anti-C1INH autoantibodies could be underestimated by conventional screening methods and represent an additional diagnostic tool.

### P-6 Role of vascular permeability factors in patients with hereditary angioedema with C1 inhibitor deficiency

#### Anne Lisa Ferrara^1^, Stefania Loffredo^1^, Maria Bova^1^, Nóra Veszeli^2^, Angelica Petraroli^1^, Chiara Suffritti^3^, Andrea Zanichelli^3^, Marco Cicardi^3^, Henriette Farkas^2^, Gianni Marone^1,4^

##### ^1^Division of Clinical Immunology and Allergy and Center for Basic and Clinical Immunology Research (CISI), University of Naples Federico II, Naples, Italy; ^2^Hungarian Angioedema Center 3rd Department of Internal Medicine, Semmelweis University, Budapest, Hungary; ^3^Department of Biomedical and Clinical Sciences L. Sacco, University of Milan, Milan, Italy; ^4^CNR Institute of Experimental Endocrinology and Oncology “G. Salvatore”, Naples, Italy

###### **Correspondence:** Anne Lisa Ferrara (anneliseferrara@gmail.com)

*Allergy, Asthma & Clinical Immunology* 2017,** 13(Suppl 2)**:P-6

**Background:** Hereditary Angioedema with C1 Inhibitor Deficiency (C1-INH-HAE) is a rare inherited genetic disease characterized by recurrent acute swelling episodes of the skin, gastrointestinal tract and upper airways resulting from increased vascular permeability. Reduced activity of C1-INH may result in an instability of kinin pathway with the generation of bradykinin inducing in increased vascular permeability. Bradykinin increases the release of nitric oxide and Vascular Endothelial Growth Factor (VEGF) from endothelial cells. VEGF, Angiopoietin 1 (Ang1) and Ang2 are released at sites of inflammation and/or angiogenesis regulating vascular permeability.We have previously demonstrated that plasma concentrations of VEGF-A, VEGF-C, Ang1, and Ang2 were higher in patients with C1-INH-HAE in remission than in healthy controls. The level of these mediators was correlated with severity of disease phenotype measured by number of attacks per year [1]. There are no data on the role of vascular permeability factors during angioedema attack and in patients on prophylaxis. In this study we analyzed plasma concentration of VEGF-A, VEGF-C, Ang1 and Ang2 in patients with C1-INH-HAE, both in remission and during acute attacks and we compared angiogenic factors plasma levels in patients taking or not prophylactic treatment.

**Methods**: Ninety-four untreated patients and 29 patients undergoing prophylaxis with attenuated androgens were studied. Then, we analysed 20 C1-INH-HAE patients in remission and during angioedema attack. Concentrations of angiogenic (VEGF-A, Ang1, Ang2) and lymphangiogenic (VEGF-C) factors were evaluated by ELISA.

**Results:** We could not detect differences in the levels of the tested factors between untreated patients and patients undergoing prophylaxis with attenuated androgens. However, these are preliminary data that we plan to expand in a follow-up study.

VEGF-A, VEGF-C and Ang2 levels were not modified during attack compared to basal conditions. By contrast, Ang1 levels (a vascular stabilizer) were increased during acute phase and the ratio Ang2/Ang1 (an index of vascular permeability) was decreased.

**Conclusions:** The results of this study show that the plasma concentrations of vascular permeability factors during acute attack changes scenario compared to basal conditions. There is an increase of vascular stabilizer and a decrease of index of vascular permeability. Moreover, preliminary experiments suggest that the prophylaxis do not modify vascular permeability factors concentration maybe because vascular preconditioning is not influenced by androgens.

**Reference**

1. Loffredo S, Bova M, Suffritti C, Borriello F, Zanichelli A, Petraroli A, Varricchi G, Triggiani M, Cicardi M, Marone G. Elevated plasma levels of vascular permeability factors in C1 inhibitor-deficient hereditary angioedema. Allergy. 2016;71:989–96.

### P-7 Expression of bradykinin B1 and B2 receptors on lymphocytes and monocytes during the remission and attack in patients with hereditary angioedema (HAE)

#### Wojciech Dyga^1^, Anna Bogdali^1^, Aleksander Obtułowicz^2^, Mikolajczyk Tomasz^3^, Ewa Czarnobilska^1^, Krystyna Obtulowicz^1^

##### ^1^Department of Clinical and Environmental Allergology, Jagiellonian University Medical College, Sniadeckich 10, 31-531, Kraków, Poland; ^2^Department of Dermatology, Jagiellonian University Medical College, Skawinska 8, Kraków, Poland; ^3^Department of Internal and Agricultural Medicine, Jagiellonian University Medical College, Kraków, Poland

###### **Correspondence:** Wojciech Dyga (wojciech.dyga@uj.edu.pl)

*Allergy, Asthma & Clinical Immunology* 2017,** 13(Suppl 2)**:P-7

Bradykinin (BK) and its receptors (BDKRB1 and BDKRB2 receptors) play important roles in a wide variety of physiological and pathological processes. As mediators of pain and inflammation, they exert a variety of biological effects on endothelium and peripheral circulation.

The aim of our study is finding a distribution pattern of the both bradykinin receptors on monocytes and lymphocytes CD4+ and CD8+ in attack of angioedema and remission of the disease.

**Method and material:** Peripheral blood mononuclear cells (PBMC) collected from 10 healthy volunteers (7 women and 3 men in the age 20–62 year) and 10 patients with hereditary angioedema type I in attack and remission of the disease (5 women and 5 men in the age 26–50 year) were isolated. Subsequently, PBMC were stained to distinguish: lymphocytes (anti-CD3, anti-CD4, anti-CD8), monocytes (anti-CD14, anti-CD16, anti HLA-DR) and their expression of B1 (anti-BDKRB1) and B2 (anti-BDKRB2) receptors and flow cytometry was performed. Also the analysis of B1 and B2 gene expression by real time PCR as a preliminary study was performed.

**Results:** We found the higher expression of BDKRB2 mainly on monocytes during attack of angioedema compering to the remission and to the reference group. The expression of BDKRB2 was highly variable in lymphocytes CD4 and CD8 both during the attack and remission. Expression of BDKRB1 was residual on both types of lymphocytes. The expression of BDKRB1 detected on monocytes was at the same level in both groups under the study. As preliminary results of gene expression the presence of mRNA for B1 and B2 receptors in PBMC was observed.

**Conclusion:** In patients with HAE and in the reference group the expression of BDKRB1 and BDKRB2 was detected both on monocytes and lymphocytes.

The expression of BDKRB1 was low contrary to the expression of BDKRB2, which was generally high in attack of angioedema. There was a huge variation between individuals in expression of BDKRB2 on lymphocytes during attack while on monocytes the expression was constantly elevated. Further studies will be performed to examine expression BDKRB2 on lymphocytes and monocytes collected form patients during HAE attack.

### P-8 Genetic variants in *SERPING1* and *F12* genes in Polish patients with hereditary angioedema type I & II

#### Anna Bogdali^1^, Teofila Książek^2^, Ewa Czarnobilska^1^, Krystyna Obtulowicz^1^

##### ^1^Department of Clinical and Environmental Allergology, The Jagiellonian University Medical College, Kraków, Poland; ^2^Department of Medical Genetics, Faculty of Medicine, Jagiellonian University Medical College, Kraków, Poland

###### **Correspondence:** Anna Bogdali (am.bogdali@uj.edu.pl)

*Allergy, Asthma & Clinical Immunology* 2017,** 13(Suppl 2)**:P-8

**Background:** Hereditary angioedema type I is a genetic disorder provoked by C1INH deficiency. The aim of the study was to find mutations in *SERPING1* and *F12* in polish patients with hereditary angioedema type I.

**Materials and methods:** The blood samples from 46 of adult patients (33 females and 13 males, age range: 18–70 years old) were collected for DNA isolation. Subsequently, *SERPING1* and *F12* coding sequence were analyzed by Sanger sequencing method, respectively for 46 and 9 patients (Applied Biosystems^®^—3500 Genetic Analyzer).

**Results:** We found mutations in *SERPING1*gene in 26 /45 patients (58%). Nevertheless, we expect more findings when our study will be completed. In our data we found several not previously reported pathogenic variants in *SERPING1* gene. We additionally sequenced *F12* gene in 9 patients carrying a mutation in *SERPING1*. In one case we found a mutation in *SERPING1* (exon 8) and a mutation in *F12* gene (exon 2). Specific pathogenic variants occurred in most cases in all family members affected with HAE. All mutations in *SERPING1* were indicated in a heterozygous state. The increased frequency of pathogenic variants was observed in 3rd and 8th exon of *SERPING1* gene.

**Conclusion:**

1. Mutations in *SERPING1* gene were presented in 58% symptomatic patients with C1INH hereditary angioedema. In one patient the mutation in exon 8 of *SERPING1* gene was joined with a mutation in exon 2 in *F12* gene.
2. Mutations in *SERPING1* gene were unique for every family with hereditary angioedema. This information can be useful for future targeted genetic testing for estimation of risk of familial re-occurrence of C1INH hereditary angioedema.

### P-9 Development of a set of sensitive assays for measuring enzyme/c1-inhibitor complexes

#### Anna Koncz^1^, Dominik Gulyás^1^,Nóra Veszeli^1^, Erika Kajdácsi^1^, László Cervenak^1^, Péter Gál^2^, József Dobó^2^, Henriette Farkas^1^, Lilian Varga^1^

##### ^1^3rd Department of Internal Medicine, Semmelweis University, Budapest, Hungary; ^2^Institute of Enzymology, Research Centre for Natural Sciences, Hungarian Academy of Sciences, Budapest, Hungary

###### **Correspondence:** Anna Koncz (anna.koncz16@gmail.com)

*Allergy, Asthma & Clinical Immunology* 2017,** 13(Suppl 2)**:P-9

**Background:** The C1-inhibitor (C1-INH) is the key regulator of complement and of contact systems and participates in the control of coagulation and fibrinolytic systems. Measuring its serum concentration is important in life-threatening angioedemas resulting from C1-INH deficiency (C1-INH-HAE). The quantity of enzyme/C1-INH complexes in the blood is possibly proportional to the level of the in vivo activation of cascade-like plasma enzyme systems. However, simultaneous, parallel determination of C1-INH-containing activation complexes has not yet been accomplished. Accordingly, it is also yet unknown, which enzyme predominates in the complexes formed with C1-INH during the edematous episodes of C1-INH-HAE. Therefore, we aimed to develop a set of sensitive, quantitative, sandwich ELISA assays for the determination of FXI/C1-INH, FXII/C1-INH, C1s/C1-INH, and C1r/C1-INH complexes.

**Materials and methods:** We developed ELISA assays using commercially available antibodies specific to FXI, FXII, C1s and C1r, as well as an affinity-purified anti-C1-INH IgG produced by our laboratory. Similarly, commercially available active FXII, and FXI, as well as active recombinant C1s, C1r and commercial plasma-derived C1-INH—further purified by ion-exchange chromatography—were used to generate the complexes required for the standards. We checked the quality of the complexes with SDS PAGE. The ELISA assays were tested on various types of blood samples (serum, and plasma anticoagulated with EDTA, citrate, hirudine, or EDTA+ protease inhibitor cocktail). The samples were collected from healthy volunteers.

**Results:** The complexes were produced from molar equivalent quantities of active enzymes and of C1-INH. In average 85.3% (70–96) of the initial enzymes formed complex with C1-INH, based on SDS-PAGE analyses. In each developed sandwich-ELISA assay the detection threshold was less than 0.01% of the plasma concentration of the enzyme. The accuracy of the recovery of the complexes was 92% (75.17–119) on average. Except C1r/C1-INH complex measurement, the serum levels of the complexes were always higher than plasma values. Intra- and inter-assay variation was 11.88% (7.51–17.55) and 15.18% (9.01–19.3), respectively.

**Conclusions:** We successfully developed a set of ELISAs for the sensitive determination of various enzyme-inhibitor complexes, which makes the simultaneous investigation of C1-INH-regulated activation systems possible in C1-INH-HAE. This could greatly contribute both to a better understanding of the pathomechanism of this disease, and to the exploration of attackkinetics. In addition to C1-INH-HAE, this method might prove suitable for studying other disorders related to the impaired regulation of plasma enzyme systems.

**Supported by:** OTKA 112110.

### P-10 Results from an interim analysis of a Ruconest treatment registry in Europe

#### R. Hakl^1^, M. Staevska^2^, H. Farkas^3^, M. Jesenak^4^, K. Hrubiskova^5^, L. Bellizzi^6^, A. Relan^6^, M. Cicardi^7^

##### ^1^St. Anne’s University Hospital of Brno, Brno, Czech Republic; ^2^University Hospital “Alexandrovska” Medical University of Sofia, Sofia, Bulgaria; ^3^Semmelweis University of Budapest, Budapest, Hungary; ^4^University Hospital of Martin, Martin, Slovakia; ^5^Bratislava University Hospital, Bratislava, Slovakia; ^6^Pharming Technologies BV, Leiden, The Netherlands; ^7^Hospital Luigi Sacco University of Milan, Milan, Italy

###### **Correspondence:** L. Bellizzi (L.bellizzi@pharming.com)

*Allergy, Asthma & Clinical Immunology* 2017,** 13(Suppl 2)**:P-10

**Background:** Hereditary angioedema (HAE) due to C1 inhibitor deficiency (C1-INH-HAE) is characterized by recurrent episodes of disabling and painful swelling. Ruconest is a recombinant human C1 inhibitor that is approved for treatment of HAE attacks. A treatment registry was established in Europe to review the adverse event profile and efficacy of Ruconest following single and repeated treatment with Ruconest.

**Methods:** Patients with C1-INH-HAE were enrolled following a decision to treat with Ruconest and after providing written informed consent. Medical history and baseline HAE information was collected at a screening visit. Treatment decisions were at the discretion of the health care professionals (HCP) involved in the patients’ care according to their standards for the management of C1-INH-HAE and in line with the approved Ruconest summary of product characteristics. Following treatment with Ruconest, the HCP entered data using a web-based questionnaire about the attack, response to therapy, and any adverse events.

**Results:** As of 28 February 2017, 45 C1-INH-HAE patients (18 male/27 female, ages 22–76 years) were treated with Ruconest in the registry for 1351 attacks in 7 European countries.

The average age at diagnosis for these patients was 24 years (range 4–68). Prior to entry in the registry, these patients experienced an average of 30 HAE attacks in the preceding year. Of the treated patients, 28.8% were on maintenance therapy/prophylaxis at enrollment. There were 653 (48.3%) abdominal, 528 (39%) peripheral, 203 (15%)facial, 21 (1.6%) laryngeal, and 24 (1.8%) urogenital attacks, including 76 attacks that involved two and one attack that involved three locations.

The mean Ruconest dose provided was 3268 units 43 U/kg (range 18–67 U/kg), patients reported relief within 4 h in 97.8% (1322/1351) of the attacks. Almost all attacks (1349/1351, 99.8%) were treated with a single dose of Ruconest. Two attacks treated with an initial dose of 2100 U (33 and 28 U/kg) received a second dose of 2100 U. No hypersensitivity or thrombotic/thromboembolic events were reported. No patients had any related serious adverse events.

**Conclusions:** The Ruconest treatment registry provides real-world data on the treatment of 1351 HAE attacks that is consistent with previous reports on the safety and efficacy of Ruconest therapy.

### P-11 The challenging management of idiopathic systemic capillary leak syndrome: survey from an Italian case series

#### Maddalena A. Wu^1^, Antonio Castelli^2^, Riccardo Colombo^2^, Gianmarco Podda^3^, Andrea Zanichelli^1^, Chiara Suffritti^1^, Marta Del Medico^1^, Avner Reshef^4^, Emanuele Catena^2^, Marco Cicardi^1^

##### ^1^Department of Biomedical and Clinical Sciences “Luigi Sacco”, University of Milan, Luigi Sacco Hospital, Milan, Italy; ^2^Intensive Care Unit, ASST Fatebenefratelli Sacco, Luigi Sacco Hospital, Milan, Italy; ^3^Medicina III, Azienda Ospedaliera San Paolo, Dipartimento di Scienze della Salute, Università degli Studi di Milano, Milano, Italy; ^4^Allergy & Immunology, Angioedema Center, Sheba Medical Center, Tel-Hashomer, Israel

###### **Correspondence:** Maddalena A. Wu (maddalena.wu@unimi.it)

*Allergy, Asthma & Clinical Immunology* 2017,** 13(Suppl 2)**:P-11

**Background:** Idiopathic systemic capillary leak syndrome (ISCLS) presents with recurrent potentially life-threatening episodes of hemoconcentration and hypovolemic shock. Due to the rarity of the disease (about 250 cases described) and to misdiagnosis, evidence based approaches as well as validated protocols are still lacking.

**Aim:** To report our experience on the treatment of acute shock emergency in patients with ISCLS.

**Materials and methods:** Analysis of records from 12 ISCLS patients (9 men), from a cohort of 22, admitted once or several times to hospital for hypovolemic shock.

**Results:** Mean age at symptoms’ onset was 51.5 years (range 35–68). ISCLS crises occurred with variable frequency (from 3 to 8), timing of the acute phase (mean duration 3–4 days) and severity. Mean follow-up was 6 years (1 month to 18 years).

*Prodromal symptoms* (identified in all subjects) consisted of: arterial hypotension (11/12 patients), fatigue (10/12), oliguria (10/12), worsening edema (9/12), weight gain (7/12), presyncopal/syncopal episodes (6/12), abdominal pain (6/12), nausea, vomiting, diarrhea (5/12), arthromyalgia (5/12), sore throat, dysphonia, cough (5/12), dizziness (4/12), high temperature (4/12), thirst/polydipsia (3/12), headache (2/12), diaphoresis (3/12), dyspnea (3/12), altered consciousness (3/12), livedo reticularis (1/12).

*The acute phase* could develop very rapidly, with alteration of vital parameters, mild to moderate alteration of consciousness and finally distributive shock, with marked hypotension, tachycardia, oligoanuria, edema (often with “stone-like” consistency, paresthesia or pain). Blood tests always revealed high hemoglobin (highest recorded value 25.8 g/dl) and hematocrit (up to 72%), hypoproteinemia (minimum serum albumin 9 g/L). IgG monoclonal band was present in all patients without changes outsides and during shock.

*Treatment* was based on crystalloids (11/12 patients), amines (11/12); 10/12 patients were treated with small boluses of high molecular weight plasma expanders (e.g. 250 mL bolus of colloids every 4 h), 7/12 steroids, 5/12 albumin, 5/12 diuretics, 1/12 methylene blue, 1/12 iv Ig. Long-term prophylaxis consisted of: verapamil (11/12), theophylline (8/12), terbutaline (2/12), iv Ig (2/12).

*Complications* recorded were: acute renal failure (10/12), compartment syndrome and neuropathy (7/12), rhabdomyolysis (5/12), myocardial edema (5/12), pericardial effusion (5/12), pleural effusion or abdominal free fluid (4/12), cerebral involvement (2/12), acute pulmonary edema (2/12), DVT (1/12). CVVH was performed in 4/12 cases, oro-tracheal intubation in 1/12, fasciotomy in 1/12, ECMO in 1/12.

**Conclusions:** ISCLS may induce severe shock associated with hemocencentration and hypoproteinemia.

Fluid replacement and amines should be minimized, with judicious use of colloids to maintain adequate perfusion. Careful surveillance of potential complications is warranted.

### P-12 Complex interplay between autonomic and contact/complement systems underlying attacks of hereditary angioedema due to C1 inhibitor deficiency

#### Maddalena A. Wu^1^, Francesco Casella^1^, Francesca Perego^1^, Chiara Suffritti^1^, Nada Afifi Afifi^1^, Eleonora Tobaldini^2^, Andrea Zanichelli^1^, Nicola Montano^2^, Marco Cicardi^1^

##### ^1^Department of Biomedical and Clinical Sciences, University of Milan, Milan, Italy; ^2^Department of Clinical Sciences and Community Health, IRCCS Ca’ Granda Foundation, Ospedale Maggiore Policlinico, University of Milan, Milan, Italy

###### **Correspondence:** Maddalena A. Wu (maddalena.wu@unimi.it)

*Allergy, Asthma & Clinical Immunology* 2017,** 13(Suppl 2)**:P-12

**Background:** Recurrences of angioedema due to C1 inhibitor deficiency (C1-INH-HAE) may have variable frequency and severity from patient to patient and in the same patient throughout life. Stressful events and hormonal changes may be triggers of attacks.

**Aim of the study:** To assess the hypothesis of a bidirectional cross-talk/interplay between autonomic nervous system (ANS) modulation and contact/complement system activation in the development of angioedema attacks.

**Materials and methods:** 23 HAE patients (6 males, mean age 47.5 ± 11.4 years) during remission and 24 healthy volunteers (8 males, mean age 45.3 ± 10.6 years) underwent recording of ECG, beat-by-beat blood pressure (BP), respiratory activity during rest (R; 10′) and 75-degrees-head-up tilt (T; 10′), together with blood withdrawals for C1INH, C4 and cleaved high molecular weight kininogen (HK). In a subgroup of patients, plasma catecholamines were also evaluated. Spectral analysis of heart rate variability allowed to extract low (LF) and high (HF) frequency components, markers of sympathetic and vagal modulation respectively.

**Results:** SAP was significantly higher in C1-INH-HAE patients than in controls (134.0 ± 19.0 vs 112.1 ± 17.4 mmHg, p = 0.001 at R, 141.4 ± 28.8 vs 121.7 ± 17.3 mmHg, p = 0.01 during T), while only in controls T induced a significant increase in mean SAP and SAP variance.

LF nu increased significantly after orthostatic challenge in both groups (69.7 ± 26.1 after T vs 57.7 ± 24.9, p < 0.05 in patients and 78.0 ± 20.7 vs 51.5 ± 21.2, p < 0.01 in controls), but only in healthy subjects there was a significant increase of LF/HF ratio, index of sympathovagal balance.

As expected, plasma C1-INH antigen and function as well as C4 antigen were markedly lower in C1-INH-HAE patients than in controls. Both proteins showed a tendency to increase after tilt.

Noradrenaline was higher in patients at R and increased in both groups after tilt test.

Cleaved kininogen (cHK), marker of contact system activation, was increased in C1-INH-HAE patients compared to controls. Tilt test induced a significant increase in cHK only in HAE patients (49.5 ± 7.5 after T vs 47.1 ± 7.8% at R, p = 0.01 in patients and 40.0 ± 6.1 vs 38.7 ± 7.2%, p = 0.06 in controls).

**Conclusions:** Our data are consistent with an alteration of ANS modulation in HAE patients, who present increased sympathetic activation at rest and blunted response to orthostatic challenge. Tilt test-induced increased HK cleavage suggests a link between stress and bradykinin production.

### P-13 Early versus late administration of icatibant in patients with hereditary angioedema

#### Irmgard Andresen^1^, Werner Aberer^2^, Teresa Caballero^3^, Laurence Bouillet^4^, Hilary J. Longhurst^5^, Andrea Zanichelli^6^, Anette Bygum^7^, Anete S.Grumach^8^, Christelle Pommie^1^, Marcus Maurer^9^, for the IOS Study Group

##### ^1^Shire, Zug, Switzerland; ^2^Department of Dermatology and Venereology, Medical University of Graz, Graz, Austria; ^3^Department of Allergy, Hospital La Paz Institute for Health Research (IdiPaz), Biomedical Research Network on Rare Diseases (CIBERER, U754), Madrid, Spain; ^4^National Reference Centre for Angioedema, Internal Medicine Department, Grenoble University Hospital, Grenoble, France; ^5^Department of Immunology, Barts Health NHS Trust, London, UK; ^6^Department of Biomedical and Clinical Sciences Luigi Sacco, University of Milan, Luigi Sacco Hospital, Milan, Italy; ^7^Department of Dermatology and Allergy Centre, Odense University Hospital, Odense, Denmark; ^8^Clinical Immunology, Faculty of Medicine ABC, Santo André, Brazil; ^9^Department of Dermatology and Allergy, Allergie-Centrum-Charité, Charité-Universitätsmedizin Berlin, Berlin, Germany

###### **Correspondence:** Irmgard Andresen (iandresen@shire.com)

*Allergy, Asthma & Clinical Immunology* 2017,** 13(Suppl 2)**:P-13

**Background:** Relationship of the timing of icatibant self-treatment to demographic and treated-attack characteristics for patients with hereditary angioedema due to C1-inhibitor deficiency are poorly understood.

**Methods:** Data from the Icatibant Outcome Survey was used to evaluate early versus late icatibant self-treatment (patients with median time-to-first injection <1 h versus ≥1 h from attack onset, respectively).

**Results:** Of 270 patients analyzed, 105 (38.9%) had median time-to-first injection <1 h (median [Q1, Q3] for 625 icatibant-treated attacks, 0.25 h [0.0, 0.5]) with no gender difference observed between early and late treating groups. Early self-treatment varied across countries, ranging from 69.6% (Germany/Austria) to 11.6% (France). Early treaters vs late treaters treated attacks localized to skin, abdomen and larynx at a similar rate (P = 0.6105, P = 0.3398 and P = 0.8219 respectively). Similarly, no statistically significant difference between early vs later treater groups was observed based on grouped attack severity (very mild/mild/moderate vs severe/very severe; P = 0.164). Comparing early versus late treatment, respectively, a significant reduction (P < 0.001) in median (Q1,Q3) time to resolution [4.5 h (1.0, 11.5) versus 8.0 h (3.0, 24.5)] and attack duration [5.0 h (1.5, 12.0) versus 13.0 h (6.0, 33.0)] was observed (243 patients; 1353 attacks with complete information on time to treatment, time to resolution and duration of attack).

**Conclusion:** Early treaters had shorter time to resolution and attack duration compared to late treaters, possibly indicating the importance of early access to icatibant in the face of HAE attacks. Differences in local practice patterns, icatibant availability, and tendency of early treaters to treat any symptoms without delay may drive prevalence of early use across countries. These and other findings from this analysis are hypothesis generating and should be further evaluated.

### P-14 Treatment administrated in attacks in hereditary angioedema during pregnancy and breastfeeding

#### Marta Sánchez-Jareño^1^, Maria Pedrosa^1^, Ana Álvez^1^, Rosario Cabañas^1^, Teresa Caballero^1,2^

##### ^1^Department of Allergy, Instituto de Investigación Hospital Universitario La Paz (IdiPaz), Madrid, Spain; ^2^CIBERER, U754, Madrid, Spain

###### **Correspondence:** Marta Sánchez-Jareño (martasanchezjare@gmail.com)

*Allergy, Asthma & Clinical Immunology* 2017,** 13(Suppl 2)**:P-14

**Background and objective:** There are only a few studies on treatment given in pregnancy and breastfeeding in patients with hereditary angioedema (HAE).

The aim of this study was to describe the pattern of treatment in acute attacks of angioedema during pregnancy and breastfeeding.

**Methods:** Retrospective analysis of data collected during pregnancies and lactation of patients attended at the Allergy Department of Hospital University La Paz by physicians specialized in the management of HAE. The following variables were collected: number of attacks in the previous six months (T1), pregnancy (T2) and breastfeeding (T3), severity of the attack, time to treatment administration, time to onset of improvement, time to complete improvement, percentage (%) of improvement 4 h after and type of acute treatment received.

**Results:** We reviewed 395 attacks of 13 patients, 18 pregnancies (2 abortions). There were no significant differences in the number of attacks among T1, T2 and T3. The number of attacks in pregnancy and breastfeeding was positively correlated (Pearson = 0.798, p = 0.017). There were no differences in the percentage of acute attacks treated with C1-inhibitor concentrate (pdC1INH) between the three periods. There were more acute attacks treated in T2 (68.9%) than in T1 (54.1%) (p = 0.044). Acute attacks were treated earlier in T2 and T3 compared to T1, although without significant differences (p = 0.327).

The time to onset of improvement was significantly different among groups (p < 0.001) (T2 3.61 ± 2.79) (T3 2.70 ± 2.45) (T1 5.54 ± 6.41). There were no differences in the time to complete improvement (p = 0.179). The percentage of improvement after 4 h was higher in T3 (57.0% ± 34.2) than in T2 (40.1% ± 33.5) (p = 0.007). The total duration of attacks was positively correlated with the time to treatment administration (p < 0.001). Mean total duration of attacks was not significantly different among groups (p = 0.980) (T1 29.28 ± 15.42) (T2 29.81 ± 76.17) (T3 28.02 ± 32.30).

**Conclusions:** This is the first study that provides data about treatment outcomes in acute attacks during pregnancy and breastfeeding in patients with hereditary angioedema. Time to onset of improvement and percentage of improvement at 4 h in breastfeeding and in pregnancy was shorter compared to the situation prior to pregnancy, although further studies are needed to confirm these results.

### P-15 Late onset of angioedema attacks due to C1 inhibitor deficiency: a diagnostic challenge

#### Marcin Stobiecki, Krystyna Obtułowicz, Wojciech Dyga, Ewa Czarnobilska

##### Medical College, Jagiellonian University, Kraków, 31-531, Poland

###### **Correspondence:** Marcin Stobiecki (mrstobiecki@gmail.com)

*Allergy, Asthma & Clinical Immunology* 2017,** 13(Suppl 2)**:P-15

**Background:** The angioedema attacks due to C1 inhibitor deficiency appear in the course of C1-INH hereditary angioedema (HAE) and C1-INH acquired angioedema (AAE). The early onset of angioedema attacks as well as positive family history are characteristic for HAE. In most cases C1-INH HAE swelling attacks start in the first decade of life. Contrary to HAE the late onset of angioedema attacks (>40 years) and negative family history are characteristic for AAE.

**Materials and methods:** The aim of the study was to analyze patients with late onset of angioedema attacks from the group of above 350 persons with angioedema attacks due to C1-INH deficiency registered in our center.

**Results:** The late onset of angioedema attacks due to deficiency of C1-INH was found in the group of 10 patients (2.9%) (Table 1). In 6 patients we confirmed AAE (group A) but in 4 cases we have no proof of any other disease or abnormality and they are still under observation (group B).

**Conclusions:** Late onset of angioedema attacks due to C1-INH deficiency may appear in patients with negative family history. Precise work up and differential diagnosis with AAE is very important.

Clinical course of the disease in the group B is identical to HAE. We also observed efficacy of treatment with C1 INH concentrates.

**Table 1:** Patients with late onset of angioedema attacks due to deficiency of C1-INH

	Group A: AAE patients	Group B: no proof for AAE
No of patients	6	4
Female/male	4/2	3/1
The age of first onset	56 ± 6 years	48 ± 6 years
Other diseases	2 patients—lymphoma, 3 patients—other npl disease (liver, kidney, breast), 1 patient—Lupus Erythematosus	1 patient—hypertension, 1 patient—renal cysts (autoimmunity and allergy diseases excluded)
Max. frequency of attacks	3–4/year	3–4/year
The most frequent localization of the attacks	facial, laryngeal	facial, laryngeal, abdominal
aC1INH	0.03–0.04 g/L	0.03–0.15 g/L
fC1INH	1.9–55%	1.4–19%
C4	0.01–0.03 g/L	0.02–0.1 g/L
C1q	39–46 mg	–
Treatment—positive response	2 patients—remission of symptoms after chemotherapy of lymphoma, 4 patients—pdC1 INH	4 patients—pdC1 INH, 1 patient rC1 INH

### P-16 Hereditary angioedema nationwide genetic study in Belarus

#### Irina Guryanova, Ekaterina Polyakova, Viktar Lebedz, Andrej Salivonchik, Svetlana Aleshkevich, Mikhail Belevtsev

##### Belorusian Research Center for Pediatric Oncology, Hematology and Immunology, Minsk region, Borovlyani, 223053, Belarus

###### **Correspondence:** Irina Guryanova (guryanovairina1985@gmail.com)

*Allergy, Asthma & Clinical Immunology* 2017,** 13(Suppl 2)**:P-16

**Introduction:** Belarus is a country in Eastern Europe with a population of 9464 million. About 100,000 children are born every year. For HAE diagnostic we use algorithm, that was shown in review by Bowen et al. Allergy, Asthma & Clinical Immunology 2010.

**Materials and methods:** During the diagnostic procedures, the levels of *C3c/C4* as well as the level of *C1*-*INH* are being determined (using *“Thermo Scientific Konelab Primer 60i”* and *“Dade Behring BN ProSpec”*). C1 function test and C1q test are carried out using “*Infosheet Anthos multimode fluorometer Zenyth 1100”.*

Next, in case of HAE type I we sequence all exons of Serping1 gene using *“Applied Biosystems 3130 Genetic Analyzer”*. In case of HAE type II we sequence three exons for finding one of this mutation in Serping1 gene: c.84-138del55, p.Ile274Val, p.Gly429Arg, p.Val454Glu, p.Arg456Glu, p.Arg458Thr(Arg), p.Arg465Val, p.Arg466Ser(His,Leu,Cys), p.Thr467Pro, p.Val473Met, p.Phe477Ser, p.Leu481Phe(Arg), p.Phe489Arg, p.Gly493Arg, p.Phe498Ser (information from https://www.nextprot.org).

**Results:** Among 49 patients we find mutations in 24 persons (22 with HAEI and 2 with HAE II). All patients with HAE I have low levels of C1 and C4. Two patients with HAE II have normal level of C1 and low level of C4.

- We find 9 splicing, 6 missense, 7 frameshift and 2 nonsense mutations. 10 of them have been found that were not described in the consulted database (http://www.ensembl.org/index.html), but in general only 6 mutations didn’t mention in ensemble.org, because we have duplicates of mutations.
- 4 small deletions affecting exon 3 (c.520-524delATCGC, p.Ser173fs254X), exon 8 (c.1293delA, p.Leu430fs449X), exon 7 (c.1106delA, p.Glu368fs396X) and exon 5 (c.744-745delCA, p.247Serfs255X) have been find. All of them are producing frame-shifts leading to premature stop codons.
- 3 missense mutations were found affecting exon 8 (c.1478 G→A, p.Gly493Glu and c.1397 G→A, p.Arg466His), exon 6 (c.1001 A→C, p.His334Pro).
- 2 mutation that affects the splice acceptor site in exon 3 (c.550 + 2T→C) and in exon 4 (c.551-1 G→A) has been identified.
- 2 nonsense mutations were found affecting exon 3 (c.289 C→T, p.Gln97Stop and c.301 C→T, p.Gln100Stop).
- 2 patients with HAE II have the same mutation in 8 exon c.1397 G→A, p.Arg466His.
- 25 patients (19 children and 6 adults) did not show any alteration in Serping1 gene, in 9 exon FXII gene, in 1 and 7 exon of ACE gene, in 5 exon of KNG1.

**Conclusion:** We found 6 mutations that didn’t mention in ensemble.org (c.520-524delATCGC, p.Ser173fs254X in exon 3; c.1293delA, p.Leu430fs449X in exon 8; c.1001 A→C, p.His334Pro in exon 6 (PolyPhen 2—0.969); c.551-1 G→A in intron “-” of 4 exon; c.301 C→T, p.Gln100Stop in exon 3; c.744-745delCA, p.247Serfs255X in exon 5).

### P-17 Off-label subcutaneous use of 1500 IE C1-INH for prophylaxis in HAE? A case report

#### Melanie Nordmann-Kleiner, Susanne Trainotti, Janina Hahn, Jens Greve

##### Department of Otorhinolaryngology Head and Neck Surgery, Ulm University Medical Center, Ulm, 89075, Germany

###### **Correspondence:** Melanie Nordmann-Kleiner (melanie.nordmann@uniklinik-ulm.de)

*Allergy, Asthma & Clinical Immunology* 2017,** 13(Suppl 2)**:P-17

**Background:** Hereditary angioedema (HAE) is a rare autosomal dominant disorder affecting approximately 1/50,000 people [1, 2]. Patients suffering from HAE show recurrent swellings of subcutaneous and submucosal structures in various regions of the body. Bradykinin-induced increased vascular permeability leads to edema formation. Current therapy consists of C1-esterase-inhibitor (C1-INH), B2 bradykinin receptor antagonists or the kallikrein inhibitor ecallantide. In most cases an on-demand therapy of acute attacks is sufficient, in severe cases, however, a prophylactic therapy is needed. Therefore C1-INH intravenously (IV) was shown to be safe and efficient.

**Methods:** We present the case of a patient with HAE-I who was under prophylactic therapy with C1-INH IV due to a high number of attacks during on-demand therapy. An implanted port guaranteed a periodical and safe apply of the medication until the device had to explanted due to an infection. Because of a bad vein status repeated IV application failed. After stopping the prophylactic therapy he suffered from recurrent and partially severe attacks again. Therefore we tried a subcutaneously off-label use of 1500 IE C1-INH as prophylaxis over more than one year.

**Results:** After a brief training session the self-application was easily managed by the patient. Under the prophylaxis the number of attacks was reduced from 4.33 to <1/month. No severe attack and none of the upper airway was noticed. The quality of life measured by the AE-Qol could be improved. The results were similar to those under the approved IV therapy.

**Discussion:** Subcutaneous use of 1500 IE C1-INH seems to be easy and safe. In our case it showed similar effectiveness compared to the IV therapy. No adverse events could be noticed. The quality of life measured by the AE-Qol could be approved. By learning a self-application the patient gained independence. The results of this case seem promising, however bigger studies are needed to underline our findings.

**References**

1 .Bygum A. Hereditary angio-oedema in Denmark: a nationwide survey. Br J Dermatol. 2009;161(5):1153–8.

2. Zanichelli A, Arcoleo F, Barca MP, Borrelli P, Bova M, Cancian M, et al. A nationwide survey of hereditary angioedema due to C1 inhibitor deficiency in Italy. Orphanet J Rare Dis. 2015;10:11.

### P-18 The importance of C1q in diagnosis of acquired angioedema (AAE)

#### Susanne Trainotti, Melanie Nordmann-Kleiner, Janina Hahn, Jens Greve

##### Department of Oto-Rhino-Laryngology Head and Neck Surgery, Ulm University Medical Center, Ulm, Germany

###### **Correspondence:** Susanne Trainotti (susanne.trainotti@uniklinik-ulm.de)

*Allergy, Asthma & Clinical Immunology* 2017,** 13(Suppl 2)**:P-18

The anamnesis is the crucial first instrument for discerning bradykinin-mediated from histamine-mediated angioedemas. Patients with onset of angioedemas in late adulthood and no family history should be considered for acquired angioedema (AAE) or late-onset hereditary angioedema (HAE) with spontaneous mutations. Once specific drug intake such as angiotensin-converting-enzyme inhibitors (ACE-inhibitors), angiotensin-II-receptor antagonists (sartans) and dipeptidyl-peptidase-4 (DPP-4) inhibitor in some oral antidiabetics is excluded, a blood test for C4, C1-esterase inhibitor antigen (C1-INH-a) and function (C1-INH-f) should be performed. As levels for these tests are low as well for HAE (type I and II) as for AAE, levels of C1q can help discern between the two pathologies. Two patients with late onset of bradykinin mediated angioedemas presented to our clinics. Both women, aged 53 and 66, showed low levels for C4, C1-INH-a and C1-INH-f, but only the younger had low C1q. AAE is known to be tightly correlated to lymphoproliferative diseases, but also anti-C1-inhibitor-auto-antibodies and other malignancies with subsequent consumption of the factors mentioned above [1]. For this woman no malignancy was known, oncological examination including PET-CT didn’t reveal any suspicious result, but she will undergo regular oncological visits. About 14% of patients with AAE don’t show any correlated disease [2]. The second patient had a history of breast cancer, splenomegaly and a submucosal gastric formation suspicious for MALT-lymphoma, an endoscopic biopsy was unsuccessful. Unexpectedly, levels for C1q were normal and testing for anti-C1-inhibitor-autoantibodies was negative. In literature there are reports about cases of AAE with normal levels of C1q [3]. As the patient mentioned that her daughter started having swellings, we had to re-consider HAE type I as diagnosis. Genetic analysis showed no mutation for SERPING-1 gene and the daughter’s blood levels for C4, C1-INH-a and C1-INH-f were normal. Even if HAE could not be excluded for sure, AAE has to be considered as the most probable diagnosis. A new endoscopic biopsy of the suspected MALT-lymphoma is already planned, as therapy of the malignant disease can stop the symptoms of AAE. In the meanwhile, she self-administers bradykinin B2 receptor antagonist for acute attacks.

C1q is an important marker to discern between HAE and AAE, especially if the correlation between AAE and lymphoproliferative diseases is considered. Even with rarely normal levels of C1q, the diagnosis of AAE should lead to clarify the presence of a malignant disease in order to detect and cure it by time.

**Consent to publish:** Consent to publish was obtained from the patients.

**References**

1. Cugno M, Castelli R, Cicardi M. Angioedema due to acquired C1-inhibitor deficiency: a bridging condition between autoimmunity and lymphoprolipheration. Autoimmun Rev. 2008;8:156–9.

2. Agostoni A, Aygören-Pürsün E, Binkley KE, et al. Hereditary and acquired angioedema: problems and progress proceedings of the third C1 esterase inhibitor deficiency workshop and beyond. J Allergy Clin Immunol. 2004;114(Suppl. 3): S51–131.

3. Castelli R. et al. Acquired C1-inhibitor deficiency and lymphoproliferative disorders: a tight relationship. Crit Rev Oncol Hematol. 2013;87:323–32.

### P-19 Hereditary angioedema in Ukraine: a national survey

#### Liudmyla Zabrodska

##### Centre of Allergic Diseases of Upper Airways, Institute of Otolaryngology by Name of O.S. Kolomiychenko of the National Academy of Medical Sciences of Ukraine, Kyiv, 03057, Ukraine

###### **Correspondence:** Liudmyla Zabrodska (zabrodskalv@gmail.com)

*Allergy, Asthma & Clinical Immunology* 2017,** 13(Suppl 2)**:P-19

Ukraine is undergoing a medical reform. The Ministry of Healthcare started paying attention to patients suffering from the rare diseases. A major obstacle to the HAE treatment is absence of medical guidelines. Medical community along with Ukrainian Association for HAE patients are closely working on the HAE treatment guideline. Along with it, other problems such as HAE diagnostics and access to medication must be also solved in order to create a quality of life for the HAE patients in Ukraine. The study focuses on the organizational aspects of the HAE diagnostic, access to medication and available treatment in Ukraine.

The HAE prevalence is estimated 1 case per 50,000 persons. Presently, in Ukraine a total of 49 patients (30 families) were identified; 47 adults and 2 children with HAE. It suggests that around 811 patients are not diagnosed and may not be treated properly especially for the acute attacks. Further search for these patients should be performed. Moreover, a proper attention must be paid to the diagnosed patients. There is no plasma-derived C1-inhibitor concentrate or other modern drug available in Ukraine. The treatment available is tranexamic acid, hormones, and fresh frozen plasma.

**Objective:** To identify all patients with HAE in Ukraine, to get for them proper medical treatment and increase awareness about the disease among the patients, doctors and the population.

**Method:** In cooperation with “Ukrainian association for HAE patients” (soon to be officially registered) to screen the country via Centres for Rare Diseases, various patient organizations to find the HAE patients. With existing patients, to conduct meetings of patients and doctor(s) to increase patients’ awareness about the disease, the symptoms and the dangerous consequences if an attack is ignored, especially the laryngeal one. To find a laboratory and financing for the blood tests to diagnose HAE. To elaborate medical guidelines for the HAE treatment. Also, to spread information through lectures, articles in popular magazines and participation in TV programs.

**Expected results:** Screen the country for the HAE patients, increase awareness about the disease among the population and get proper diagnostic and medical care for the HAE patients. The risk of upper airway obstruction underlines the importance of diagnosing the HAE patients and increasing their awareness about life threatening situations that might occur.

### P-20 Development of a sensitive assay for measuring C1-inhibitor protein

#### Dominik Gulyás, Nóra Veszeli, Erika Kajdácsi, László Cervenak, Henriette Farkas, Lilian Varga

##### 3rd Department of Internal Medicine, Semmelweis University, Budapest, Hungary

###### **Correspondence:** Dominik Gulyás (ggddoommiinniikk@gmail.com)

*Allergy, Asthma & Clinical Immunology* 2017,** 13(Suppl 2)**:P-20

**Background:** Determining C1-inhibitor (C1-INH) concentration is necessary to the diagnosis of life-threatening angioedemas based on C1-INH deficiency (C1-INH-HAE and C1-INH-AAE), as well as for the differential diagnosis of other forms of angioedema. The current tests cannot detect the small concentration differences found during the exploration of the pathomechanism of the disease. Our aim was to develop a quantitative, sensitive, sandwich ELISA assay, which would be appropriate also for research purposes. This method would allow studying the kinetics of C1-INH regulation by analyzing blood samples from a C1-INH-HAE patient at different times during an angioedematous attack.

**Methods:** We used our proprietary rabbit antiserum, as well as the affinity-purified version of the latter (anti-C1-INH_aff_). We used a human plasma-derived C1-INH concentrate and its ultra-purified form (uC1-INH) to determine C1-INH concentration. We measured C1-INH concentrations in serum obtained from 109 type I and from 11 type II C1-INH-HAE patients Our result were compared with our previous data based on radial immunodiffusion (RID) and functional (Quidel) tests.

**Results:** We drafted a reproducible ELISA assay protocol. We applied 0.025 μg/ml anti-C1-INH_aff_ as coating; our detection antibody was 2.5 μg/ml biotinylated anti-C1-INH_aff_. Two-fold serial dilutions in eight steps from 0.1 μg/ml of uC1-INH served as standards. We minimized the background along with the sensitivity threshold. The limit of detection was 0.4 ng/ml with a dynamic range of 1–100 ng/ml. We succeeded in re-measuring the uC1-INH samples of 20 ng/ml concentration with 98% accuracy on average. C1-INH concentration measured with our ELISA was significantly higher than with RID in case of type I C1-INH-HAE patients and controls, whereas it was significantly lower in type II C1-INH-HAE patients. ELISA and RID exhibited a moderate correlation with each other (Spearman’s r = 0.3617, *p* = 0.0001), and both correlated with the result of the functional test (r = 0.3952, *p* < 0.0001, and r = 0.5342, *p* < 0.0001).

**Conclusions:** Using our anti-C1-INH_aff_ and uC1-INH reagents, we developed a high-sensitivity test, which can be readily implemented also in laboratories where RID is not in routine use. Polyclonal antibodies obtained from commercial sources may cross-react with other proteins—this can be avoided by affinity purification. On one hand, the substantial difference between the concentrations determined by ELISA or RID may arise, in the case of RID, from a possible cross-reaction. On the other hand, it suggests that ELISA might recognize additional (e.g. inactive, complexed) forms of C1-INH present in the samples from the patients.

**Supported by:** OTKA 112110.

### P-21 Hereditary angioedema in a Brazilian cohort: delay in diagnosis

#### Maria L. Oliva Alonso^1^, Solange O. R. Valle^1^, Anete S. Grumach^2^, Rosangela P. Tórtora^1^, Alfeu T. França^1^, Marcia G. Ribeiro^1^

##### ^1^Federal University of Rio de Janeiro, Rio de Janeiro, Brazil; ^2^Faculty of Medicine ABC, São Paulo, Brazil

###### **Correspondence:** Maria L. Oliva Alonso (mloalonso@yahoo.com.br)

*Allergy, Asthma & Clinical Immunology* 2017,** 13(Suppl 2)**:P-21

**Objective:** To describe the delay between first symptoms and diagnosis in a cohort of patients with hereditary angioedema (HAE) followed up at a Reference Center in Rio de Janeiro, Brasil.

**Materials and methods:** A descriptive cross-sectional study with prospective data collection of 138 patients followed up at a Brazilian Outpatient clinic specializing in HAE, from 1989 to 2016. The following parameters were evaluated: sex, age, age at onset of symptoms, age at diagnosis, familial history and severity of HAE attacks.

**Results:** Data from 138 patients, 97 (70.3%) females and 41 (29.7%) males were collected. We found a preponderance of HAE with C1-INH deficiency (HAE-C1-INH) (n = 107; 77.5%), followed by HAE with normal C1-INH (n = 31; 22.5%). Their average ages ranged from 12 to 77 years (mean age = 39.0 ± 15.4 years). Time between early manifestations and diagnosis ranged from 0 (early diagnosis) to 61 years (mean = 17.0 ± 12.9 years). Familial history of HAE was observed in 89.9% of cases. Most of them (75.6%) had moderate or severe attacks. Nineteen were asymptomatic (13.8%).

**Conclusion:** We found a long time between the early manifestations and diagnosis of HAE, even in those patients with a positive familial history. About 75% of cases presented moderate to severe symptoms. Early diagnosis and successful treatment is critical to survival and to improve quality of life. Screening of family members, including asymptomatic individuals, improved the number of cases detected. The delay until HAE diagnosis reflects the need of widespread information about the disease among clinicians in order to decrease morbidity and mortality.

### P-22 An investigation into the importance of body mass index in case of patients with hereditary angioedema caused by C1-inhibitor deficiency

#### Tamás Szilágyi, Nóra Veszeli, Kinga Viktória Kőhalmi, Lilian Varga, Henriette Farkas

##### Hungarian Angioedema Center, 3rd Department of Internal Medicine, Semmelweis University, Budapest, Hungary

###### **Correspondence:** Tamás Szilágyi (szila10@gmail.com)

*Allergy, Asthma & Clinical Immunology* 2017,** 13(Suppl 2)**:P-22

**Background:** Hereditary angioedema with C1-inhibitor deficiency (C1-INH-HAE) is characterized by recurrent angioedema mediated by bradykinin release. Previous studies have found an increased factor XII activity in case of obese patients with hypertension. As factor XII activated contact system has a particular importance in C1-INH HAE, our study aimed to investigate if there is any relationship between the body mass index (BMI) and the number of edematous attacks.

**Materials and methods:** The 128 adult patients (75 females and 53 males, mean age 45 and 43 years, 22 females and 22 males took danazol regularly) and 31 children (17 girls and 14 boys, mean age 11 and 12 years respectively) attended an annual follow-up visit in the Hungarian HAE Center. Their data has been registered in the Hungarian HAE Registry. For those who are above 18 years old, we used BMI values, while in case of children the percentiles (defined by their BMI values) were taken into consideration. The patients were grouped based on their BMI, gender, age and prophylactic treatment. We determined the annual frequency of edematous attacks in accordance with BMI in each group (underweight, normal, overweight, obese).

**Results:** The average BMI of adult patients was 25.95 which is just above the lower border of the overweight category. There was no significant difference among the BMI categories either in case of men or women, however, by comparing the similar groups of the two genders, we found that women had significantly higher number of edematous attacks than men in all cases (except in the underweight category because of the small number of patients).Higher number of edematous attacks was observed in children with higher percentiles than 95%. Patients who took danazol (attenuated androgen) suffered the similar number of edematous attacks as those who did not take it regularly, but the patients taking danazol as long-term prophylaxis, had significantly higher BMI values (p = 0.001). Regarding the BMI values and the average number of edematous attacks (yearly), there was no significant difference between groups taking lower or higher doses of danazol.

**Conclusions:** According to our results, we can state that the BMI has no impact on the frequency of edematous attacks. Women have more edematous attacks than men. Children with lower weight are less affected by the symptoms of C1-INH-HAE. Taking danazol on a regular basis, results in significantly higher BMI values.

### P-23 The clinical appearance of idiopathic nonhistaminergic acquired angioedema and its comparison to other hereditary angioedema forms

#### Noémi Andrási, Nóra Veszeli, Kinga Viktória Kőhalmi, Dorottya Csuka, Lilian Varga, Henriette Farkas

##### Hungarian Angioedema Center, 3rd Department of Internal Medicine, Semmelweis University, Budapest, Hungary

###### **Correspondence:** Noémi Andrási (nono.andrasi@gmail.com)

*Allergy, Asthma & Clinical Immunology* 2017,** 13(Suppl 2)**:P-23

**Background:** The pathomechanism of idiopathic nonhistaminergic acquired angioedema (InH-AAE) is not yet fully understood. It is characterised by recurrent angioedema without wheals. It is not easy to diagnose this type of angioedema. InH-AAE is considered when no other diagnostic criteria are fulfilled and recurrence could not be prevented by antihistamine therapy. Our objective was to examine the clinical characteristic of InH-AAE and to compare it to hereditary forms.

**Methods:** We examined 46 patients with InH-AAE who received medical care in our center (Hungarian Angioedema Center) between 2009 and 2015. In these patients, we excluded histaminergic angioedema, acquired angioedema related to angiotensin converting enzyme inhibitor, C1-inhibitor deficiency, and hereditary angioedema due to a mutation of the coagulation factor XII (FXII-HAE) as additional diseases. We compared InH-AAE patients’ clinical parameters with 27 patients diagnosed with hereditary angioedema of unknown origin (U-HAE) and with 73 patients diagnosed with hereditary angioedema due to C1-inhibitor deficiency (C1-INH-HAE).

**Results**: In the InH-AAE group, median age at the diagnosis was 41 years. Median age at onset of symptoms was 36 years. Compared to that, in the C1-INH-HAE group, median age at onset of symptoms was 13 years, and in the U-HAE group was 29 years. Additionally, 52% of patients with InH-AAE, 59% of patients with C1-INH-HAE and 41% of patients with U-HAE reported 12 or more edematous attacks per year. When it comes to the localization of the symptoms, edema of the limbs occurred in 92% of patients with CI-INH-HAE, versus 54% of patients with InH-AAE, and 37% of patients in U-HAE. 67% of patients with InH-AAE, and 59% of patients with U-HAE suffered edema of the face, but only 15% of patients with C1-INH-HAE mentioned face edema. About half of the patients (51%) with C1-INH-HAE, and only about ¼ of the patients with InH-AAE (28%) and with U-HAE (29%) reported edema of the larynx. edema of the gastrointestinal tract occurred in 75% of patients with C1-INH-HAE. However, in both, InH-AAE and U-HAE, only about 20% of patients mentioned edema of the bowel.

**Conclusion:** It appears that clinical symptoms in the InH-AAE group are different compared to the C1-INH-HAE group. Based on this, it can be assumed that there are different mechanisms that contribute to the onset of the edema. The clinical symptoms were similar in the case of the InH-AAE and the U-HAE group which implies that the underlying pathomechanisms are also similar in these two.

### P-24 Home treatment with conestat alfa in attacks of hereditary angioedema due to C1-inhibitor deficiency

#### Nóra Veszeli^1^, Kinga Viktória Kőhalmi^1,2^, Lilian Varga^1,2^, Henriette Farkas^1,2^

##### ^1^Research Laboratory, 3rd Department of Internal Medicine, Semmelweis University, Budapest, 1125, Hungary; ^2^Hungarian Angioedema Center, 3rd Department of Internal Medicine, Semmelweis University, Budapest, 1125, Hungary

###### **Correspondence:** Nóra Veszeli (veszeli.nora88@gmail.com)

*Allergy, Asthma & Clinical Immunology* 2017,** 13(Suppl 2)**:P-24

**Background:** Conestat alfa, a recombinant C1-inhibitor (rhC1-INH) is a novel therapeutic option for the acute treatment of hereditary angioedema due to C1-INH deficiency (HAE-C1-INH).

Our aim was to continue to investigate the efficacy and safety of conestat alfa administered as acute treatment under real-life conditions to relieve angioedema attacks in patients with C1-INH-HAE.

**Materials and methods:** We analyzed 376 edematous episodes requiring acute treatment and occurring in 7 C1-INH-HAE patients. The patients were treated at home with a dose 2100 U rhC1-INH per occasion. The patients recorded the time of rhC1-INH administration; the time to the onset of improvement and to the complete resolution of symptoms; and noted any side effects. Symptom severity and patient satisfaction were measured with a visual analogue scale (VAS).

**Results:** 165 HAE attacks occurred in abdominal viscera, 9 in the upper airways, 174 in subcutaneous and 62 in multiple locations. RhC1-INH was administered 85.0 (0.0–2910.0) [median (min–max)] minutes after the onset of the attacks with a severity (upon injecting) of 60.0 (10.0–99.0) on a VAS. Clinical symptoms improved within 60.0 (0.0–1320.0) minutes, and the complete resolution of symptoms took 840.0 (60.0–4320.0) minutes. The time between the onset of the attack and the administration of rhC1-INH correlated with the time that symptoms stopped worsening (R = 0.2194, p < 0.0001), with the time that symptoms improve (R = 0.2575 p < 0.0001) and with the time to the complete resolution of symptoms (R = 0.3512, p < 0.0001). Second and third injection of rhC1-INH was administered in 11 and 1 attacks respectively, because symptoms did not improve or resolve completely. In seven cases, rhC1-INH was administered with the aim of prevention before dental procedure. Neither of the cases were followed by edematous attack.

None of the patients experienced a recurrence of the attack, or drug-related systemic adverse events. The mean VAS score of patient satisfaction was 93.1.

**Conclusions:** Home treatment with rhC1-INH was an effective and well-tolerated therapy for all types of HAE attacks. Early treatment of attacks resulted in the better outcomes.

### P-25 Canadian physician survey on the medical management of hereditary angioedema

#### Lisa Fu^1^, Amin Kanani^2^, Gina Lacuesta^3^, Susan Waserman^4^, Stephen Betschel^1^

##### ^1^Department of Medicine, Division of Clinical Immunology and Allergy, University of Toronto, Toronto, ON, Canada; ^2^Division of Allergy and Immunology, Department of Medicine, University of British Columbia, Vancouver, BC, Canada; ^3^Department of Medicine, Dalhousie University, Halifax, NS, Canada; ^4^Division of Clinical Immunology Allergy, McMaster University, Hamilton, ON, Canada

###### **Correspondence:** Lisa Fu (lisa.wy.fu@gmail.com)

*Allergy, Asthma & Clinical Immunology* 2017,** 13(Suppl 2)**:P-25

**Background:** Hereditary angioedema (HAE) is a rare disease that has significant morbidity and may be potentially fatal due to airway obstruction. Our study aimed to determine how Canadian physicians diagnose and treat HAE.

**Methods:** A survey was designed to determine HAE practice patterns amongst Canadian physicians who treat HAE. These physicians were identified by sending the survey to members of three physician organizations (Canadian Hereditary Angioedema Network, Canadian Society of Clinical Immunology and Allergy, Canadian Hematology Society) whose members were thought to most likely treat HAE patients.

**Results:** Thirty-six physicians responded to the survey. The majority of referrals to HAE treating physicians were from family and emergency physicians. The most common sites of swelling reported by patients to physicians were facial, peripheral and abdominal. Facial, abdominal and laryngeal swellings were reported to impact the patient’s quality of life the most. 20 out of 35 respondents reported they used an attack diary to monitor patients. A mean of 53.9% of HAE-Type 1 and II patients and 53.4% of HAEnC1INH patients were on long term prophylaxis. A mean of 43.8, 18.8 and 93.8% of respondents had some patients on danazol, tranexamic acid and C1-inhibitor respectively. Within this group, there was a mean of 24.4, 34.2, and 79.6% of patients on danzol, tranexamic acid and C1-inhibitor respectively.

The majority of physicians felt severity and frequency of attacks were the most important determinants in deciding when to use prophylaxis. A mean of 86.1% of physicians used C1-inhibitor to treat acute attacks and 77.8% used icatibant. A mean of 91.4 and 94.3% of respondents felt very or extremely confident using C1-inhibitor for prophylaxis and for acute attacks respectively. A mean of 71.4% of physicians felt very or extremely confident in using icatibant.

A total of 35/35 respondents were aware of HAE guidelines, 33/35 used the guidelines to manage their patients. Respondents felt a further need for guidance when managing HAE in pregnancy and pediatric patients.

**Conclusions:** Physicians are using guidelines to support their practice, and using agents suggested by guidelines with confidence. C1-inhibitor is being used widely for prophylaxis, as well as acute treatment of attacks along with icatibant. However certain special patient populations may require additional focus in future guidelines.

### P-26 Ambiguous symptoms of hereditary angioedema may delay diagnosis of concomitant diseases: two case reports

#### Inmaculada Martinez Saguer, Carmen Escuriola Ettingshausen, Zeynep Gutowski, Richard Linde

##### Haemophilia Centre Rhine Main, Frankfurt-Mörfelden, Germany

###### **Correspondence:** Inmaculada Martinez Saguer (inmaculada.martinez@hzrm.de)

*Allergy, Asthma & Clinical Immunology* 2017,** 13(Suppl 2)**:P-26

**Background:** Hereditary angioedema is a rare autosomal dominant disease caused by C1-inhibitor deficiency (C1-INH-HAE) with symptoms of recurrent oedema attacks in different regions of the body. Abdominal C1-INH-HAE attacks are often mistakenly diagnosed as food intolerance, irritable bowel syndrome, and appendicitis, among others. Here we aim to bring attention to the reverse situation: symptoms of other conditions may be confused with C1-INH-HAE attacks. Two case reports are presented to illustrate this situation.

**Materials and methods:** Two patients, a 10-year old boy and a 54-year old woman, with C1-INH-HAE were followed for symptoms and clinical cause of the disease. All attacks, treatment, and response to treatment were documented in patient diaries. In both patients, pdC1-inhibitor (C1-INH) replacement therapy was generally well tolerated and effective. When the frequency of abdominal attacks increased considerably without any obvious triggers, the symptoms could be temporarily managed by additional doses of C1-INH concentrate. Relief was only short-lasting and repeated dosing was needed. To assess the cause of the sudden increase in frequency of abdominal attack oedema, C1-INH-testing, gastroscopy, colonoscopy, and metabolic tests were conducted.

**Results:** C1-INH testing was negative in both cases, with C1-INH levels after infusion within the expected ranges. Further examinations for the boy revealed a fructoseintolerance. Under a specific diet, the frequency of abdominal symptoms decreased back to what it was before. The gastroscopy for the woman revealed gastritis. With administration of pantoprazole 40 mg, the high-frequency abdominal pain stopped and her disease was now again well-controlled with her usual C1-INH replacement regimen.

**Conclusions:** These two case reports show that with a diagnosis of C1-INH-HAE, symptoms of other conditions may be misinterpreted as oedema attacks. It is therefore important to instruct patients to seek medical advice if the frequency of their attacks increases unexpectedly and their otherwise effective C1-INH-HAE therapy fails to provide lasting relief.

**Consent to publish:** Consent to publish was obtained from the patients mentioned in this abstract.

### P-27 First kinetic follow-up of symptoms and complement parameters during a hereditary angioedema attack

#### Nóra Veszeli^1,2^, Kinga Viktória Kőhalmi^1,2^, Erika Kajdácsi^1^, Dominik Gulyás^1^, György Temesszentandrási^2^, László Cervenak^1^, Henriette Farkas^1,2^, Lilian Varga^1,2^

##### ^1^Research Laboratory, 3rd Department of Internal Medicine, Semmelweis University, Budapest, 1125, Hungary; ^2^Hungarian Angioedema Center, 3rd Department of Internal Medicine, Semmelweis University, Budapest, 1125, Hungary

###### **Correspondence:** Nóra Veszeli (veszeli.nora88@gmail.com)

*Allergy, Asthma & Clinical Immunology* 2017,** 13(Suppl 2)**:P-27

**Background:** Hereditary angioedema due to C1-inhibitor (C1-INH) deficiency (C1-INH-HAE) is characterized by recurrent, self-limited, edematous attacks. C1-INH deficiency leads to uncontrolled activation of the complement- and kallikrein-kinin systems, resulting in bradykinin release. For the first time, we studied the kinetics of C1-INH and other complement parameters, as well as their role in a self-limited edematous attack in a C1-INH-HAE patient.

**Materials and methods:** We monitored the severity of the symptoms during the entire observation period, i.e. at baseline, in the prodromal, and during-attack stages, and at complete resolution. Twelve blood samples were obtained from the patient, and five were collected from a healthy control during a 24-h period. We measured C1-INH concentration and activity (C1-INH_c+a_), C1(q,r,s), C3, C4, C3a, C4a, C5a and SC5b-9 levels.

**Results:** After a 24-h symptom-free period and another 19-h prodromal period, the patient had a 29-h-long edematous attack in multiple locations, and was followed up for another day. The highest C1-INH_c+a_, C4, and C1(q,r,s) levels were measured at baseline, and their continuous decrease was observed during the 96-h observation period. C1-INH_c_ was 0.033 g/l at the onset of edematous attack. C4 level was depleted when edematous symptoms reached the maximum severity and stayed excessively low level also after the complete resolution of symptoms. Compared to baseline, C4a level was four times higher 18 h before the onset of the attack. The levels of other complement activation parameters were lower than the control values, which remained consistently stable.

**Conclusions:** The concentration and activity of C1-INH decreased progressively before edematous attack. Interestingly, we could not detect any increase of C1-INH after the spontaneous resolution of the attack. This suggests that factors other than C1-INH may be important in the resolution of edematous attacks. C4a may be a useful biomarker for the prediction of edematous attacks.

**Consent to publish:** Both subjects gave written informed consent for the publication.

This study was supported by the grants OTKA 112110 (LV) and by the ÚNKP-16-3 New National Excellence Program of the Ministry of Human Capacities”.

### P-28 Hereditary angioedema in Mexican paediatric population

#### Melissa I. Espinosa^1,2^, Francisco A. Contreras^2,3^, Sandra A. Nieto^2,3,4^

##### ^1^Allergy and Clinical Immunology, Allergy Department, Instituto Nacional de Pediatría, Mexico City, Mexico; ^2^Clinical Allergy Department, Instituto Nacional de Pediatría, Mexico City, Mexico; ^3^Nutritional Genetics Department, Instituto Nacional de Pediatría, Mexico City, Mexico; ^4^Asociación Mexicana de Angioedema Hereditario, Mexico City, Mexico

###### **Correspondence:** Melissa I. Espinosa (melissaen@hotmail.com), Sandra A. Nieto (sandranietoaeh@gmail.com)

*Allergy, Asthma & Clinical Immunology* 2017,** 13(Suppl 2)**:P-28

**Background:** Hereditary Angioedema is a rare disease with C1 inhibitor deficiency (HAE-C1INH). It has been described that the initial presentation of symptoms varies from 4.4 to 18 years, with mean age of ten years. Furthermore, the presentation of the first attack at early age is related to a severe course of the disease with more frequency and severity of the attacks [1].

In our country there are no publications about the prevalence and clinical presentation of hereditary angioedema in paediatric population.

We present the paediatric population from The National Institute of Paediatrics (INP) with hereditary angioedema.

**Results:** We report 14 cases with clinical and laboratory diagnosis of hereditary angioedema, from the Hereditary Angioedema Clinic in the Allergy Department at INP. They are now 13 months to 18 years old of age, 92.8% have family history, suggesting 7.14% with *novo* mutation; 64% are women (3 of them are presumably C1-INH normal and no FXII mutation type), and 78% are type I. The mean age of the first attack was 2 years and 6 months, the median age was also 2 years and 6 months old, and the earliest age at initial symptoms was 2 months old. The mean age at diagnosis was 7 years. 86% of patients had cutaneous involvement, 64% had gastrointestinal symptoms at any time since diagnosis and 64% had laryngeal involvement at least once, with the youngest patient at 2 months of age for laryngeal symptoms (confused as a whipping cough reactivation every month until the specific treatment). One patient reported an asthmatic-like attack, which improved rapidly after infusion of plasma derived C1-INH. 50% of our patients have used plasma-derived CI-INH at least once for acute attacks only. Because of the poor availability of this treatment, 43% of patients were treated with nadroparin for acute attacks, with successful improvement of the symptoms. We do not use danazol in children because the adverse effects reported in this population. No deaths have been reported in paediatric patients at our Institution.

**Conclusions:** Misdiagnosis is common in paediatric population, especially in those with initial gastrointestinal symptoms or atypical manifestation as asthmatic-like attack. Subcutaneously administered Nadroparin has been reported to be effective for symptoms resolution [2], however it is not recommended in current guidelines. As there is poor availability of plasma derived C1-INH in our country, further studies on nadroparin are needed to establish it as an alternative treatment.

**References**

1. Farkas H, Martinez-Saguer I, Bork K, Bowen T, Craig T, Frank M, Germenis AE, Grumach AS, Luczay A, Varga L, Zanichelli A, on behalf of HAWK. International consensus on the diagnosis and management of pediatric patients with hereditary angioedema with C1 inhibitor deficiency. Allergy. 2017;72:300–13.

2. Majiluf A, Nieto S. Long-term follow up analysis of nadroparin for hereditary angioedema. A preliminary report. Int Immunopharmacol. 2011;11:1127–32.

### P-29 Management of hereditary angioedema in Slovak Republic—national survey results

#### Milos Jesenak^1^, Katarina Hrubiskova^2^, Martin Hrubisko^3^, Ludmila Vavrova^4^, Peter Banovcin^1^

##### ^1^Center for Hereditary Angioedema, Comenius University in Bratislava, Jessenius Faculty of Medicine, University Teaching Hospital, Martin, Slovakia; ^2^Center for Hereditary Angioedema, Comenius University in Bratislava, Faculty of Medicine, University Teaching Hospital, Bratislava, Slovakia; ^3^Department of Clinical Immunology and Allergology, St. Elisabeth’s Oncology Institute, Bratislava, Slovakia; ^4^Department of Medical Genetics, St. Elisabeth’s Oncology Institute, Bratislava, Slovakia

###### **Correspondence:** Milos Jesenak (jesenak@gmail.com)

*Allergy, Asthma & Clinical Immunology* 2017,** 13(Suppl 2)**:P-29

**Background:** Hereditary angioedema (HAE) belongs to the inherited defects of complement system with unique characteristics which separate it from the whole group of primary immunodeficiencies. Due to better understanding of pathophysiology of oedemas development, novel therapeutic strategies were developed. The availability of different therapeutic options differs among various countries. Recently, based on the national survey, 94 living patients with HAE in Slovakia, a country in eastern central Europe with population over 5 million people, were found and identified.

**Methods and patients:** The national study about the number of HAE patients and their selected characteristics was performed through the questionnaire-based survey in out-patient clinics for allergy and clinical immunology. The study was conducted by Center for HAE in University Teaching Hospital in Martin. Current management approaches and selected characteristics of the HAE patients were analysed.

**Results:** Our group consisted from 94 living patients with HAE (aged 37.52 years; 55% males) from 42 families. The most prevalent form was HAE type I (86%), followed by HAE type II (9%) and type III (5%). The majority of the patients was managed in two National Centers for Hereditary Angioedema (Martin, Bratislava), however, a small proportion of HAE patients has not been referred to the centers. Typical clinical presentation in the majority of the patients (67%) was characterised by the combination of various symptoms (skin, gastrointestinal, laryngeal, and genital). Isolated skin symptoms were found in 17%, isolated gastrointestinal in 7% and isolated laryngeal angioedemas only in 3% of the patients. Six patients were still asymptomatic. Erythema marginatum was presented in 13% as a prodromal sign of ongoing attack. The mostly used prophylactic therapy was attenuated androgen (in 44% of patients), followed by tranexamic acid (in 13%) and C1-INH concentrate (either human or recombinant) in 2% of the subjects. However, 39% patients remained without any prophylactic therapy. In 15 patients, various forms of thrombophilia was confirmed, which excluded the used of anti-fibrinolytics. Despite current guidelines, 30% of the patients used as a rescue therapy androgens. 26% were equipped by icatibant, 23% with pd-C1-INH and 21% with rh-C1-INH. Molecular diagnosis was performed in 69% of patients and 12 new, previously non-described mutations were detected.

**Conclusions**: Since the first publication of HAE series from former Czechoslovakia [1], this is the first report about the current situation in HAE prevalence and management in Slovakia. Our survey detected expected number of the patients with the estimated prevalence of 1:57,700 and revealed some problems in the management of HAE patients.

**Reference**

1. Starsia Z, Hegyi E, Kuklinek P, et al. Hereditary angioedema in Czechoslovakia. Clinical, immunological, genetic and therapeutic studies of 16 families. Allerg Immunol. 1988;34:35–42.

### P-30 Quality of life in 41 patients with hereditary angioedema: first report from Iranian National Registry of Hereditary Angioedema

#### Maryam Ayazi^1^, Mohammad Reza Fazlollahi^1^, Shiva Saghafi^1^, Sajedeh Mohammadian^1^, Susan Nabilou Deshiry^1^, Kiana Bidad^1^, Raheleh Shokouhi Shoormasti^1^, Iraj Mohammadzadeh^2^, Mohammad Hassan Bemanian^3^, Seyed Alireza Mahdaviani^4^, Zahra Pourpak^1^

##### ^1^Immunology, Asthma and Allergy Research Institute, Tehran University of Medical Sciences, Tehran, Iran; ^2^Non-Communicable Pediatric Diseases Research Center, Babol University of Medical Sciences, Babol, Iran; ^3^Department of Clinical Immunology and Allergy, Hazrate Rasoul Hospital, Iran University of Medical Sciences, Tehran, Iran; ^4^Pediatric Respiratory Diseases Research Center, National Research Institute of Tuberculosis and Lung Diseases (NRITLD), Shahid Beheshti University of Medical Sciences, Tehran, Iran

###### **Correspondence:** Maryam Ayazi (m-ayazi@farabi.tums.ac.ir, marie.ayazi@yahoo.com)

*Allergy, Asthma & Clinical Immunology* 2017,** 13(Suppl 2)**:P-30

**Background:** Hereditary angioedema (HAE) is a serious condition that may not only lead to fatal laryngeal edema attacks but also disfiguring swelling episodes, severe abdominal pains and unnecessary surgeries, which disrupt health-related quality of life (HRQoL) considerably. Therefore, the aim of this study was to assess and report the HRQoL in Iranian HAE patients for the first time.

**Materials and methods:** Patients suspected of having HAE referred (2006–2016) to Immunology, Asthma and Allergy Research Institute (IAARI) were fully evaluated after signing informed consent. Those with a definite diagnosis of HAE entered in the Iranian HAE registry (IHAER). Registered patients older than 15 years completed Iranian validated version of 36-item Short-Form Health Survey (SF-36) [1], which is a general quality of life instrument measuring 8 health-related concepts: Physical Functioning, Role Limitations due to Physical and Emotional Problems, Bodily Pain, General Health, Vitality, Social Functioning, Perceived Mental Health, and also Health Transition.

**Results:** Out of 63 eligible registered patients 41 (female = 24, male = 17) with the mean age of 37.60 ± 12.2 were enrolled in this study, while 22 patients could not be contacted due to a change of domicile, emigration or death (2 cases). The SF-36 showed a reduction in General Health in patients with HAE when compared with healthy Iranian population [1]. Men had better scores in all domains, which was significant in Physical Functioning (*p* = 0.001). Mean scores of eight domains ranged from General Health (61.62) to Physical Functioning (88.37) (Table 1). Mean physical and mental status scores were 75.5 and 69.17, respectively. 56% of patients reported better health condition compared to one year ago.

**Conclusions:** Low score of General Health in Iranian HAE patients despite rather good scores in physical domains might indicate the strong impact of mental/emotional domains like depression and nervousenss on quality of life. HRQoL in female patients seem to be more affected by HAE, which could stem from higher importance of physical appearance and emotional susceptibility in this group, and other sociocultural factors. This study shows HAE patients are suffering a lower HRQoL because of their medical condition, which is even exacerbated by other factors including: (1) misdiagnosis, mistreatment and delayed diagnosis owing to rare nature of HAE; (2) not having easy access to guideline-based emergency/prophylaxis treatments especially in developing/underdeveloped countries. However, improvement in health condition of Iranian HAE patients within the last year might be the result of IHAER efforts to ameliorate awareness and facility for this disease.

**Table 1:** Scores of SF-36 domains in 41 Iranian patients with HAE

SF-36 health-related domains	Mean ± SD
Physical functioning	88.37 ± 12.97
Role limitations/physical	72.56 ± 35.70
Role limitations/emotional	67.47 ± 39.07
Vitality	62.25 ± 18.36
Perceived Mental Health	68.40 ± 16.94
Social functioning	77.18 ± 23.32
Pain	73.23 ± 24.78
General health	61.62 ± 24.81

**Reference**

1. Montazeri A, Goshtasebi A, Vahdaninia M, Gandek B. The Short Form Health Survey (SF-36): translation and validation study of the Iranian version. Qual Life Res. 2005;14(3):875–82.

### P-31 Hereditary angioedema in Bulgaria: clinical and therapeutic characteristics

#### Anna Valerieva^1^, Mariela Vasileva^2^, Tsvetelina Velikova^3^, Elena Petkova^1^, Vasil Dimitrov^1^, Maria Staevska^1^

##### ^1^Clinic of Allergy and Asthma, Medical University of Sofia, University Hospital “Alexandrovska”, Sofia, Bulgaria; ^2^Department of Surgery, Medical University of Sofia, University Hospital “Alexandrovska”, Sofia, Bulgaria; ^3^Laboratory of Clinical Immunology, University Hospital St. Ivan Rilski, Medical University-Sofia, Sofia, Bulgaria

###### **Correspondence:** Anna Valerieva (anna.valerieva@gmail.com)

*Allergy, Asthma & Clinical Immunology* 2017,** 13(Suppl 2)**:P-31

**Background**: Hereditary angioedema (HAE) is a rare and potentially life-threatening disease. No data has been published about HAE in Bulgaria.

**Method**: Our aim was to identify the clinical characteristics and therapeutic modalities of HAE within a cohort study of the Bulgarian population.

**Results**: The number of patients diagnosed with HAE was 70 (26 HAE families): 38 female (54.3%) and 32 (45.7%) male, calculating the prevalence of the disease to be 1:103,785. The distribution of families between HAE Type 1 and 2 was: 21 (81%) vs. 5 (19%). Family history was present in 55 subjects (78.5%), while de-novo mutation was presumed in 15 (21.5%). Age-related characteristics and age distribution in groups are presented in Tables 1 and 2.

**Table:** **Table 1**

	Age (n = 61)	Age at onset of symptoms (n = 49)	Age at diagnosis (n = 45)	Diagnosis delay (n = 45)	Follow-up (n = 45)
Mean, years (±SE)	42 (±2.5)	11.1 (±1.6)	22.5 (±2.6)	11.2 (±2.2)	21.3 (±2.1)
Range	3–82	1–50	2–70	0–62	1–46

**Table:** **Table 2**

Age of first HAE symptoms (n = 49)	0–10 years	11–20 years	>21 years
No of patients	31 (63.3%)	15 (30.6%)	3 (6.1%)

Family history of HAE related death was reported in 37 patients (53%). Based on the frequency of attacks patients are grouped in 5 categories (n = 53) (Table 3).

**Table:** **Table 3**

Asymptomatic	0–1 attacks/month	2–4 attacks/month	5–9 attacks/month	≥10 attacks/month
4 (7.5%)	4 (7.5%)	23 (43.4%)	14 (26.5%)	8 (15.1%)

The location of attacks data is presented in Table 4.

**Table:** **Table 4**

	Peripheral (n = 70)	Facial (n = 62)	Abdominal (n = 61)	Laryngeal (n = 59)	Urogenital (n = 49)
Yes	68 (97.2%)	58 (93.5%)	56 (91.8%)	37 (52.8%)	28 (57.1%)
None	2 (2.3%)	4 (6.5%)	5 (8.2%)	33 (47.2%)	21 (42.9%)

Treatment modalities are presented in Table 5.

**Table:** **Table 5**

	Androgens (past) (n = 49)	FFP (past) (n = 53)	Androgens-ongoing LTP (n = 65)	C1inh on demand (n = 60)	C1inh self-administration (n = 70)
Yes	20 (40.8%)	40 (75.5%)	5 (7.7%)	None—5 (8.3%)	No 67 (95.7%)
None	29 (59.2%)	13 (24.5%)	60 (92.3%)	pdC1inh 8 (13.3%)	Yes 3 (4.3%)
				rhC1inh 47 (78.3%)	

**Conclusions**: HAE is a rare disease and the diagnosis is often delayed. The overall characteristics of Bulgarian HAE patients do not show major differences from other reported populations. Frequency of attacks is reported to be rather high in the Bulgarian cohort. Treatment options were limited in the past, while nowadays the great majority of patients have access to C1inh concentrate.

### P-32 A global multicenter registry of patients with different forms of angioedema without urticaria

#### Francesca Perego^1^, Ruggero Di Maulo^2^ on behalf of participating centers

##### Department of Biomedical and Clinical Sciences “Luigi Sacco”, University of Milan, Luigi Sacco Hospital, Milan, Italy; ^2^CEO Cloud-R s.r.l., Milan, Italy

###### **Correspondence:** Francesca Perego (francescappe@gmail.com)

*Allergy, Asthma & Clinical Immunology* 2017,** 13(Suppl 2)**:P-32

**Background:** Different forms of angioedema occurring without wheals have been classified (Cicardi et al. Allergy 2014;69:602–16), data on each form are minimal and have never been collected systematically.Our aim is to gather in a single registry clinical and laboratory data on the various forms of angioedema, providing better characterization to improve diagnostic and therapeutic approaches.

**Materials and methods:** Starting from an Italian database of patients with C1-INH-HAE provided by the Italian network ITACA, a web based multi-center registry was created with the support of the Italian HAE Association. The registry was built to include patients with acquired (idiopathic histaminergic, idiopathic non histaminergic, associated to ACE-inhibitor, acquired with C1-inhibitor deficiency) or hereditary angioedema (with C1-inhibitor deficiency; with normal C1-INH and mutation in Factor XII or of unknown origin). The following data are collected: patients’ personal and demographic data; clinical and laboratory characteristics; major comorbidities; angioedema treatments; location, duration, severity and treatment of angioedema attacks and prophylaxis. The registry has also functionalities to support patient’s follow-up and a continuous improvement of quality and completeness of data. In November 2016 representatives of angioedema centers from 18 counties (Serbia, France, Spain, Italy, Hungary, Greece, Macedonia, Brazil, Romania, Mexico, Colombia, Canada, Greece, Poland, Bulgaria, Poland, Bulgaria, Canada) met in Sofia (Bulgaria) to switch the registry from national to global. At this writing changes are ongoing to move the registry under the umbrella of the International HAE association. It presently contains 937 entries from Italy, the number is expected to rapidly grow with the contribution of centers from other countries. The governance of the registry will be in charge to a committee where all centers and patients’ supporting groups are represented. The technical handling is provided by the data managing society Cloud-R s.r.l., which developed the new registry on the cloud. Thanks to the application architecture of the registry, patients will be able to directly provide part of the information and to retrieve individual data via web or mobile. Centers will own and will be able use the data they provide. The committee will manage aggregated data and will release periodic outputs. Data will be available for health and regulatory agencies according to the law.

**Conclusions:** The first disease registry for different forms of angioedema has been built. It is open globally, scalable, secure and it will provide prospective data to expand the understanding of the disease and to improve the standard of care.

### P-33 Improvement in diagnostic delays over time in patients with hereditary angioedema: findings from the Icatibant Outcome Survey

#### Andrea Zanichelli^1^, Markus Magerl^2^, Hilary J Longhurst^3^, Werner Aberer^4^, Teresa Caballero^5^, Laurence Bouillet^6^, Anette Bygum^7^, Anete S Grumach^8^, Christelle Pommie^9^, Irmgard Andresen^9^, Marcus Maurer^10^

##### ^1^Department of Biomedical and Clinical Sciences Luigi Sacco, University of Milan, ASST Fatebenefratelli Sacco, Milan, Italy; ^2^Department of Dermatology and Allergy, Allergie-Centrum-Charité, Charité-Universitätsmedizin Berlin, Berlin, Germany; ^3^Department of Immunology, Barts Health NHS Trust, London, UK; ^4^Department of Dermatology and Venereology, Medical University of Graz, Graz, Austria; ^5^Department of Allergy, Hospital La Paz Institute for Health Research (IdiPaz), Biomedical Research Network on Rare Diseases (CIBERER, U754), Madrid, Spain; ^6^National Reference Centre for Angioedema, Internal Medicine Department, Grenoble University Hospital, Grenoble, France; ^7^Department of Dermatology and Allergy Centre, Odense University Hospital, Odense, Denmark; ^8^Faculty of Medicine ABC, São Paulo, Brazil; ^9^Shire, Zug, Switzerland; ^10^Department of Dermatology and Allergy, Allergie-Centrum-Charité, Charité-Universitätsmedizin Berlin, Berlin, Germany

###### **Correspondence:** Andrea Zanichelli (andrea.zanichelli@unimi.it)

*Allergy, Asthma & Clinical Immunology* 2017,** 13(Suppl 2)**:P-33

**Introduction:** Hereditary angioedema due to C1 inhibitor deficiency (C1-INH-HAE) is a rare, oftentimes misdiagnosed disease, with consequent delays in diagnosis and associated morbidity. We analyzed patient data from the Icatibant Outcome Survey (IOS) to evaluate the relationship between patient age (birth date) and both age at diagnosis and delay in diagnosis (time between first symptom onset and diagnosis).

**Methods:** The IOS is an international, observational study (NCT01034969). Data were collected from 12 countries between July 2009 and January 2017. Study participants provided written informed consent. Patients with a family history of C1-INH-HAE who were diagnosed before their first symptoms were excluded from analysis. To avoid potential bias inherent in the data collected from the youngest generation (time to diagnosis is limited by their age), only patients born before 1990 and diagnosed before 25 years of age were included. Linear regression analyses were performed.

**Results:** Of 685 patients with C1-INH-HAE type I or II in the IOS as of January 2017, 250 patients were included in the analysis. The median age at diagnosis was 16.7 years and the median delay in diagnosis was 2.6 years. When analyzed by year of birth, the median age at diagnosis declined over time, being 20.2 years in patients born from 1950–1960 and 15.4 years in patients born from 1980–1990 (P = <0.0001; Pearson correlation coefficient, r = −0.2659). There was also a reduction in diagnostic delays, being 7.0 years in patients born from 1950–1960 and 1.4 years in patients born from 1980–1990 (P = 0.0029; r = −0.1874; Table 1).

**Conclusion:** Although delay in diagnosis of patients with C1-INH-HAE has improved over time, there remains a need to raise awareness of HAE.

**Table:** **Table 1**

Year of birth	N	Median age at diagnosis, years (interquartile range)	Median delay in diagnosis, years (interquartile range)
1950 < 1960	24	20.2 (18.96–22.17)	7.0 (0.25–13.59)
1960 < 1970	59	18.1 (14.46–20.38)	6.0 (0.46–14.63)
1970 < 1980	70	13.7 (7.79–19.30)	2.4 (0.13–5.63)
1980 < 1990	94	15.4 (8.71–18.80)	1.4 (0.00–5.96)
All	250	16.7 (10.05–19.79)	2.5 (0.05–9.71)
P value		<0.0001	0.0029
r		−0.2659	−0.1874

### P-34 A survey of hereditary angioedema in Israel

#### Iris Leibovich-Nassi^1^, Avner Reshef^1^, Raz Somech^2^, Hava Golander^3^

##### ^1^Angioedema Center, Sheba Medical Center, Tel-Hashomer, 5265601, Israel; ^2^Safra Pediatric Hospital, Sheba Medical Center, Tel-Hashomer, 5265601, Israel; ^3^Department of Nursing, Sackler School of Medicine, Ramat Aviv, 6997801, Israel [All affiliated to the University of Tel Aviv, Israel]

###### **Correspondence:** Iris Leibovich-Nassi (Iris.leibovich@sheba.health.gov.il)

*Allergy, Asthma & Clinical Immunology* 2017,** 13(Suppl 2)**:P-34

**Background:** Hereditary angioedema (HAE) is a rare genetic disorder affecting 1:50,000 people. Remarkable progress has been made over the last decade, due to a renewed interest in the disease, better diagnosis and new treatment modalities. The State of Israel (pop. 8,500,000) comprises ethnically heterogeneous society, which undergoes dynamic socioeconomic changes and witnessing constant waves of immigration from Asia, America, Europe and Africa.

**Methods:** We undertook a survey of physician-diagnosed HAE patients (type I & II) from all regions of Israel.* A specific 55-item questionnaire was constructed, addressing demographics, age of diagnosis, disease course, triggers, burden of disease (quality-of-life) and effect of prodromes. Questions were phrased as categorical or continuous (i.e. scales).

**Results:** Total screened population was 233 pts, 197 (84.5%) responded: 113 (57.3%) females and 84 (42.6%) males. Of the 123 pts with accurate diagnosis 105 (84.7%) were type I, and 18 (14.5%) type II. Mean age was 35.4 years, (range 1–79). 53 pts (26.9%) were minors (<18 years). Mean number of children per family was 2.03, with 78 (54%) reporting at least one child diagnosed. 169 (86%) reported having other diagnosed family members. 119 (60%) reported at least one sibling diagnosed, 145 (74%) reported having at least one parent, 73 (37%) at least one uncle or aunt and 90 (46%) an affected grandparent, disease course was variable. 132 (67%) have been hospitalized or visited ER at least once, and 60 (30%) underwent intubation or ICU admission. Additionally, 39 (20%) reported multiple attacks per week, 34 (17%) 3–4 attacks per month, 70 (36%) 1–2 per month, 24 (12%) once or twice per year and 30 (15%) reported no attacks at all. 101 pts (51%) were using HAE prophylaxis (Danazol, Tranexamic Acid or C1-INH).

Almost all the symptomatic participants (n = 165; 99%) reported experiencing prodromes along their disease course. 55 (28%) claimed that prodromes were an absolute predictor of attacks, in 57 (29%) it was considered an occasional predictor, 34 (17%) experienced it ‘sometimes’ and 32 (16%) reported no association between prodromes and attacks.

**Conclusions:** This is the first comprehensive survey of HAE in Israel. We hope that the data obtained will illuminate unmet medical as well as psychosocial needs, and help to provide better care for our patients.

(We would like to acknowledge the most helpful collaboration of Prof. S. Kivity, Prof. M. Rottem, Prof. E. Toubi and the Israeli HAE Patient Association-EDEMA)

### P-35 Delayed diagnosis of hereditary angioedema in an adult patient. A case report

#### Erika J. Sifuentes^1^, Francisco A. Contreras^2^, Sandra A. Nieto^3,4^ *

##### ^1^Allergy and Clinical Immunology, Allergy Department, Instituto Nacional de Pediatría, Mexico City, Mexico; ^2^Allergy Department, Instituto Nacional de Pediatría, Mexico City, Mexico; ^3^Nutrition and Genetics Department, Instituto Nacional de Pediatría, Mexico City, Mexico; ^4^Mexican Association of Hereditary Angioedema, Mexico City, Mexico

###### **Correspondence:** Erika J. Sifuentes (erikasifuentes@gmail.com, sandranietoaeh@gmail.com)

*Allergy, Asthma & Clinical Immunology* 2017,** 13(Suppl 2)**:P-35

**Background:** Hereditary angioedema (HAE) can become a clinical challenge to diagnose. Patients experience recurrent local swelling in different parts of the body including the intestine [1]. Swelling of the intestine may cause symptoms suggestive of an acute abdomen, and this may mislead physicians to consider surgical treatment.

We described a case of a 42 year old man with abdominal pain attacks for the past 8 years. The patient described the attacks of abdominal pain as abrupt and quite intense, sometimes associated with nausea or vomit. He only described two episodes, where in addition of the abdominal pain he experienced local swelling of his forearm and hand, the swelling had no apparent cause and it resolved in less than 3 days. During one of his attacks he underwent open appendicectomy, but the histopathology result was negative for appendicitis. Five years after, he experienced another severe attack of abdominal pain that again brought him to the emergency department. As part of his medical approach an abdominal CT scan was performed, showing peripancreatic and subdiaphragmatic fluid along with mesenteric lymphadenitis. He persisted with acute abdominal pain, so he underwent an exploratory laparotomy from which a biopsy of a mesenteric lymph node was taken and ascitic fluid sent for culture. Under the suspicion of infection, he received antibiotic, but afterwards the histopathology report showed only unspecific inflammatory response, and no bacteria was found in the ascetic fluid culture. To continue studying the cause of his abdominal attacks the patient also had a push enteroscopy showing esophagitis and erosive gastritis, histopathology report from stomach, duodenum, and jejunum biopsies were normal.

In the course of one of his visits to the emergency department, aside from the clinical suspicion of pancreatitis, finally angioedema was considered as a differential diagnosis. Hereditary angioedema with C1-INH deficiency type I was confirmed with laboratory tests (Table 1).

**Table:** **Table 1**

Test name	Result	Reference range
C4	5 mg/dL	16–47 mg/dL
C1q	24.2 mg/dL	10–25 mg/dL
C1-inhibitor function	23%	≥68% normal
		41–67 equivocal
		≤40 abnormal
C1-inhibitor concentration	4 mg/dL	Low mg/dL

**Conclusions:** Abdominal pain attacks occurring in HAE are a diagnostic problem that may lead to laparotomy. Although HAE remains as a rare disease, we consider a simple test as a determination of C4 levels should be performed during the initial approach to every patient presenting appropriate clinical manifestations of HAE.

**Consent to publish:** Consent to publish was obtained from the patient.

**Reference**

1. Hofman ZLM, Relan A, Hack CE. Hereditary angioedema attacks: local swelling at multiple sites. Clin Rev Allergy Immunol. 2016;50(1):34–40.

### P-36 Emergency call number for isolated angioedema: a 4 month experience in France

#### Isabelle Boccon-Gibod, Catherine Mansard, Laurence Bouillet

##### CREAK, CHU Grenoble, Médecine Interne, Grenoble, France

###### **Correspondence:** Catherine Mansard (cmansard@chu-grenoble.fr)

*Allergy, Asthma & Clinical Immunology* 2017,** 13(Suppl 2)**:P-36

**Background:** The isolated angioedema, in particular facial, is a motive which leads the patients to consult in Emergencies. We established an emergency telephone number for the doctors in Emergencies, regular doctors and allergists, 24/24 h.

**Materials and methods:** We listed all the calls from September, 2016 till January, 2017 having required an emergency appeal in front of an isolated angioedema in departments around the CHU of Grenoble (Isère, Ain, Savoie, Haute-Savoie). Every patient benefited from a consultation in the national reference center of the angioedema (CREAK) within 15 days from the call.

**Results:** We received 34 calls concerning 20 women and 10 men with a mean age of 60 years [33–87]. All the calls concerned an isolated facial edema. 75% of the patients were sent by the department of Emergencies, 13% by the regular doctors and 11% by allergists. On 34 patients, 6 did surrender to the consultation. For 32% of the patients the diagnosis of bradykinin-induced angioedema (angiotensin converting enzyme drugs), 3% bradykinin-induced angioedema (ARAII drugs) and for 41% histamine-induced angioedema was done. The modifications of care in Emergencies matters mainly concerned the therapeutic indications with 75% of the patients having benefited from corticoids before the call and the necessity of setting up a treatment adapted to a bradykinin angioedema in 18% of bradykinin-induced angioedema (concentrated C1Inh or icatibant). All the diagnoses were confirmed in consultation and 100% of the dosages of realized inhibitory C1 are normal, only 2 patients are in expectations of results.

**Conclusions:** All the new diagnoses of hereditary bradykinin angioedema were done directly at the center and patient didn’t arrive in an emergency Department. The diagnosis of bradykinin-induced angioedema (angiotensin converting enzyme drugs) represent more than one third of the calls and it was unexpected. The emergency number was helpful for the therapeutic care of the angioedema. The long-term follow-up of these patients will allow to confirm or not this diagnosis and to see again in one year if the proportion of bradykinin-induced in our region.

### P-37 Hereditary angioedema: analysis of 287 attacks treated with Berinert^®^ in the French Cohort COBRA

#### Laurence Bouillet^1,2^, Isabelle Boccon-Gibod^1,2^, Anne Pagnier^1,2^, Anne Gompel^2,3^, Bernard Floccard^2,4^, Ludovic Martin^2,5^, Claire Blanchard-Delaunay^2,6^, David Launay^2,7^, Olivier Fain^2,8^, Alain Sobel^9^, Stéphane Gayet^2,10^, Stéphanie Amarger^11^, Guillaume Armengol^2,12^, Yann Ollivier^2,13^, Ariane Zélinsky-Gurung^2,14^, Pierre-Yves Jeandel^2,15^, Gisèle Kanny^2,16^, Brigitte Coppéré^2,17^, Marie Dubrel^2,18^, Fabien Pelletier^2,19^, Aurélie Du Thanh^2,20^, Sébastien Trouiller^21^, Jérôme Laurent^22^, Claire De Moreuil^2,23^, Christine Audouin Pajot^2,24^, Alexandre Belot^2,25^

##### ^1^Department of Internal Medicine, Grenoble University Hospital, Grenoble, France; ^2^National Reference Centre for Angioedema, CREAK, Grenoble, France; ^3^Endocrine Gynecology, Paris Descartes University, University Hospital Centre, Paris, France; ^4^Intensive Care Unit, Edouard Herriot Hospital, Hospices Civils de Lyon, Lyon, France; ^5^Department of Dermatology, CHU Angers, Angers, France; ^6^Department of Internal Medicine, CH Niort, Niort, France; ^7^Université Lille Nord de France, Department of Internal Medicine, National Reference Centre for Autoimmune and Systemic Rare Diseases, Hospital Claude-Huriez, CHRU Lille, Lille, France; ^8^Department of Internal Medicine, Saint Antoine University Hospital, AP-HP, Paris, France; ^9^Hotel Dieu University Hospital, AP-HP, Paris, France; ^10^Department of Internal Medicine, La Timone Hospital, Marseille, France; ^11^Department of Dermatology and Cutaneous Oncology, CHU Clermont-Ferrand, Clermont-Ferrand, France; ^12^Department of Internal Medicine, Bois-Guillaume Hospital, CHU Rouen, Bois-Guillaume, France; ^13^Department of Internal Medicine, CHU Caen, Caen, France; ^14^Department of Pediatric Emergency, CH Niort, Niort, France; ^15^Department of Internal Medicine, CHU Nice, Nice, France; ^16^Department of Internal Medicine and Clinical Immunology, CHU Nancy, Nancy, France; ^17^Department of Internal Medicine, Edouard Herriot Hospital, Hospices Civils de Lyon, Lyon, France; ^18^Department of Internal Medicine-Pediatry, Saint Antoine University Hospital, AP-HP, Paris, France; ^19^Department of Dermatology, Jean Minjoz Hospital, CHU Besançon, Besançon, France; ^20^Department of Dermatology, CHU Montpellier, Montpellier, France; ^21^Department of Internal Medicine, Henri-Mondor Hospital, CH Aurillac, Aurillac, France; ^22^Department of Allergology, HEGP Hospital, AP-HP, Paris, France; ^23^Department of Internal Medicine, CHU Brest, Brest, France; ^24^Department of Internal Medicine, CHU Toulouse, Toulouse, France; ^25^Department of Pediatric Internal Medicine, Mother Children Hospital, Hospices Civils de Lyon, Lyon, France

###### **Correspondence:** Laurence Bouillet (lbouillet@chu-grenoble.fr)

*Allergy, Asthma & Clinical Immunology* 2017,** 13(Suppl 2)**:P-37

**Introduction:** The COBRA registry reports clinical data and treatment response with Berinert^®^ in the French population of patients with bradykinin mediated angioedema (AE): Hereditary AE (HAE) with and without C1Inh deficiency, idiopathic non histaminergic AE, drug induced AE.

**Methods:** We conducted an analysis of patients with HAE included in the *Cohort Berinert Angiœdème* (COBRA) registry study from 2007 to November 2016, which aims to collate information on all Berinert^®^-treated patients with HAE throughout France. The analysis included retrospective data extracted from patients’ medical record and prospective data directly recorded in the electronic registry. Data on treatment response was only available for documented attacks after COBRA enrollment.

**Results:** 132 type I–II HAE patients, 34.4 ± 17.8 years old, (66.7% women) are today included in the registry. They were 13.4 ± 10.8 yo when they had the first attack and 18.4 ± 14.6 when the diagnosis was made. They have been treated with Berinert^®^ during the last 3.7 ± 4.4 years and the first treatment was received when they were 30.2 ± 17.1 yo. 47.8% of them were genotyped and 92.7% were presenting a Serping1 mutation. Among them 105 patients (79.5%) presented at least an attack treated with Berinert^®^. These patients presented 287 attacks: abdominal (29.0%), facial (22.0%), laryngeal (14.0%), peripheral (10.0%) and multi-location (25%). 60.0% of the attack were rated “severe”, 38.0% “moderate” and 2% “low”. A trigger event was described in 25.4% of crisis in relation with stress or trauma for 15.0% of the cases. 39.8% (98) of attacks have been treated with 20 UI/kg, 54% (135) with 10–20 UI/kg. On available data, symptoms began to be improved in less than 1 h in 35.0% and in less than 2 h in 27%. Symptoms disappeared in less than 24 h in 68.5%. A treatment failure occurred for 8 out 287 attacks (11.1%): 4 attacks were treated with a low dose (<20 UI/kg), 1 attack was treated 8 h after starting, no precise information are available for the 3 remaining attacks. Patients’ satisfaction rate was 94.6%.

**Conclusion:** COBRA registry affords the opportunity to systematically describe type I–II bradykinin mediated AE patients treated with Berinert^®^ and to monitor its efficacy in attack treatment.

### P-38 Management of hereditary angioedema: C1-inhibitor esterase and icatibant acetate self-administration for acute attacks

#### Ana Rodríguez, Dasha Roa, Alicia Prieto, Maria Luisa Baeza

##### Department of Allergy, Hospital General Universitario Gregorio Marañón, Madrid, Spain

###### **Correspondence:** Ana Rodríguez (anarf@me.com)

*Allergy, Asthma & Clinical Immunology* 2017,** 13(Suppl 2)**:P-38

**Background:** Acute angioedema attacks in hereditary angioedema (HAE) are unpredictable; they may cause activity impairment and may be disfiguring, painful or even life-threatening. Specific treatment is administered through subcutaneous or intravenous route. Attending to healthcare facilities cause economic costs, absenteeism from work and delay in treatment. Home- and self-administration programs reduce morbidity, disease burden and potential mortality, improving quality of life.

**Materials and methods:** In this observational retrospective study, HAE patients attending to our third level Hospital, were offered a home self-administration training program. They followed 2–6 sessions lasting for 45 min with a specialist nurse. One partner for each patient was also considered. Injection/infusion skills were annually reviewed.

The number of annual angioedema attacks and their need for emergency department (ED) attendance for the last 5 years was registered.

**Results:** 31 HAE patients were included, 19 women (61.29%), mean age 47.81 years (18.63 SD); 22 patients were categorized as C1-INH HAE (18 type I and 4 type II) and 9 patients of two families as FXII-HAE. Two type II C1-INH HAE and 2 FXII-HAE patients were free of symptoms so far.

21 patients followed the training program. 14 patients were trained for subcutaneous icatibant self-injection and another 2 patients preferred a family member to be trained. 8 patients as well as their 8 family partners learned intravenous C1-inhibitor esterase infusion. Only 2 patients refused the training program and preferred to attend to a near sanitary center for treatment administration.

The median annual number of angioedema attacks was 16.05 (C1-INH HAE type I 17.6, type II 9). Patients with FXII-HAE were asymptomatic in periods without hyperestrogenic states. Oropharyngeal angioedema developed in 55.5% of attacks in C1-INH HAE type I, 50% of type II and 25% of FXII-HAE patients. For the last 5 years, 1 patient attended to the ED 4 times and 4 patients only once due to poor control of attack despite of treatment. Neither of them required orotracheal intubation nor hospitalization.

**Conclusions:** Despite the high numbers of annual angioedema attacks and the significant proportion of oropharyngeal involvement, the number of visits to the ED was very low with no hospital admissions. Therefore, self-administration programs decrease disease burden and improved patient’s quality of life.

### P-39 Off-label intramuscular administration of Conestat Alfa (rhC1inh) in HAE patients: a case series

#### Anna Valerieva, Borislava Krusheva, Elena Petkova, Vasil Dimitrov, Maria Staevska

##### Clinic of Allergy and Asthma, Medical University of Sofia, Sofia, Bulgaria

###### **Correspondence:** Anna Valerieva (anna.valerieva@gmail.com)

*Allergy, Asthma & Clinical Immunology* 2017,** 13(Suppl 2)**:P-39

**Background:** Conestat alfa (rhC1inh) is registered for intravenous treatment of hereditary angioedema (HAE) attacks in adult and adolescent patients.

**Methods:** We present a case series of three different HAE patients who use off-label intramuscular administration of rhC1inh.

**Results:** Case 1: 32-year old man weighing 60 kg with HAE type I. Intramuscular application of rhC1inh has been practiced since 2015 during peripheral, abdominal and urogenital attacks: when he presumes that an attack is not life-threatening, intramuscular self-administration is preferred. The patient normally injects a 20 ml syringe (3000 U rhC1inh + solvent). Frequent under-dosing is reported (1 vial, 2100 U rhC1inh, reconstituted in 10 ml). The patient self-injects the medication in the middle front third of m. quadriceps femoris.

Case 2: A 50 kg, 16-year old girl with HAE type I (daughter of Case 1): during non-severe HAE attacks the father injects 17 ml (2500 U rhC1inh + solvent) in the middle front third of the thigh of his daughter. Again, under-dosing is frequent with good clinical outcome.

Case 3: 71-year old HAE type I patient (84 kg) with frequent and severe abdominal attacks (2–3 times per week). The patient suffers compromised veins and difficult peripheral venous access often resulting in treatment impediments. Off-label twice weekly prophylactic intramuscular administration of rhC1inh was initiated after all ethical implications were discussed and this common decision was made. The dose used per application: 4200 U/20 ml (2 vials solved in 10 ml solvent, each) was injected intramuscularly in two different sites, either into m. gluteus maximus or m. quadriceps femoris. During the 14-week follow-up no severe breakthrough attacks occurred. The patient reported significant improvement in the quality of life and daily activities were restored.

No side effects at the application site and from the medication were reported from any of the patients.

**Conclusions**: Intramuscular administration of rhC1inh could be an alternative to the intravenous route of application, especially when intravenous administration is compromised or access to medical care facilities is difficult. Intramuscular application of rhC1inh seems to be safe and effective in the presented cases: as on-demand therapy and in prophylaxis. 2100 U rhC1inh can be successfully solved in 10 ml solvent and the intramuscular application shows no adverse effects. Long-term prophylaxis (LTP) with rhC1inh seems to be safe and effective in subjects with severe HAE. Application of two vials (4200 IU) twice weekly seems to be an effective dose for LTP.

**Consent to publish:** Consent to publish was obtained from the patients.

### P-40 Are we ready to propose a pharmacological approach for Hereditary Angioedema with normal C1 Inhibitor (HAEnlC1INH)?

#### Stephanie K. A. Almeida^1^, Camila Lopes Veronez^2^, Rosemeire N. Constantino-Silva^1^, Nyla Melo^1^, Joanna Araujo Simoes^1^, Sandra Mitie U. Palma^1^, Joao Bosco Pesquero^2^, Anete S. Grumach^1^

##### ^1^Clinical Immunology, Faculty of Medicine ABC, Santo Andre, SP, Brazil; ^2^Laboratory of Biophysics, Federal University of Sao Paulo, São Paulo, SP, Brazil

###### **Correspondence:** Anete S. Grumach (asgrumach@gmail.com)

*Allergy, Asthma & Clinical Immunology* 2017,** 13(Suppl 2)**:P-40

**Background:** Hereditary angioedema with normal C1 inhibitor (HAEnlC1INH) was first described in 2000. It affects both genders with higher prevalence off females due to hormonal influence. Symptoms have been associated with bradykinin and part of the patients present *factor 12* mutation. Considering the limited knowledge about the mechanism involved, therapy for HAE with C1INH deficit has been applied to those cases. We evaluated the response to therapy of symptomatic patients with HAEnlC1INH proposing a personal approach.

**Methods:** Patients with suggestive symptoms of HAE, familial history and normal C1-INH were included. DNA samples were evaluated for the presence of mutations on exon 9 of the *F12* gene. The protocol was approved by ethical committee.

**Results:** Fourteen families (8 with *factor 12* mutation) were included within a total of 38 (33F:5M) patients (median age 34.5; 9–69 years). Initial symptoms appeared at 18.5 years old (median) and 23/29 between 10–30 years old. Three families reported bruising as prodromal symptoms. Edema mainly affected the following: abdomen 27/29; face 19/29; extremities 12/29; upper airways 13/29. Prophylactic therapy was: combined contraceptive exclusion 8/24; tranexamic acid (medium dosage 500–750 mg/day) 20/29; progestins 15/24; danazol 9/24; oxandrolone 3/24. The attacks were treated with higher dosages of tranexamic acid in 9/29 patients; icatibant 8/29 and plasma derived C1INH in 4/29.

**Conclusions:** Although recognition of physiopathology in HAEnlC1INH is restricted, we face the need of therapy in these patients. Approximately 2/3 of the patients present high risk for upper airway obstruction. We propose a stepwise approach for those patients: combined contraceptive exclusion; low dosages of tranexamic acid; progestins and finally androgens as prophylaxis. High dosage of tranexamic acid was effective for mild symptoms during the attacks but icatibant and plasma derived C1 inhibitor had been used preferentially.

### P-41 New mutations in C1 inhibitor gene leading to hereditary angioedema

#### Camila L. Veronez^1^, Jane da Silva^2^, Bruna F. de Azevedo^1^, Solange Valle^3^, Eli Mansour^4^, Anete S. Grumach^5^, Alvin H. Schmaier^6^, João B. Pesquero^1^

##### ^1^Department of Biophysics, Federal University of São Paulo (UNIFESP), São Paulo, SP, Brazil; ^2^Serviço de Alergia, University Hospital Professor Polydoro Ernani de São Thiago, Federal University of Santa Catarina, Florianópolis, SC, Brazil; ^3^Department of Clinical Immunology, Federal University of Rio de Janeiro, Rio de Janeiro, RJ, Brazil; ^4^Department of Clinical Allergy and Immunology, Faculty of Medicine, State University of Campinas, Campinas, SP, Brazil; ^5^Outpatient Group of Recurrent Infections and Laboratory of Clinical Immunology, Faculty of Medicine ABC, Santo André, SP, Brazil; ^6^University Hospitals Cleveland Medical Center, Case Western Reserve University, Cleveland, OH, USA

###### **Correspondence:** Camila L. Veronez (camila.lopesveronez@mdc-berlin.de)

*Allergy, Asthma & Clinical Immunology* 2017,** 13(Suppl 2)**:P-41

**Background:** From the1960 s, biochemical studies indicated that the deficiency of C1 esterase inhibitor (C1-INH) isthe disorder of hereditary angioedema (HAE) [1, 2].Only since the 1980s, have mutations in its encoding gene (*SERPING1*) described in HAE families [3, 4]. Currently, more than 500 mutations have been found in *SERPING1* that are associated with HAE according to The Human Genome Mutation Database (https://portal.biobase-international.com/cgi-bin/portal/login.cgi). Here we describe new mutations in *SERPING1* found in HAE patients.

**Materials and methods:** Complete coding sequence of *SERPING1* was sequenced by Sanger method from patients with HAE clinical characteristics and low plasma levels of C1-INH and C4.The Ethical Committee of UNIFESP approved the study and patients signed a consent form.

**Results:** Six new mutations were found in 6 unrelated patients: 3 small deletions (c.195delG and c.309_312delCCAA in exon 3, and c.953_954delCA in exon 6), 1 duplication (c.576_581dupCCTGGAGA in exon 4), 1 base change affecting the acceptor splice site from intron 4 (c.686-1G>A), and a nonsense mutation (p.Lys329stop in exon 6). The deletion c.953_954delCA was found in a patient from USA; all the other mutations were found in Brazilian patients.The change p.Lys329stop was confirmed to be a de novo alteration by sequencing the affected patient’s parents. Only one patient reported family history (c.195delG carrier).In addition, 3 mutations previously described in the literature were found for the first time in Brazilian patients (Q348X, Q392X, c.1249 + 1G>A) [5, 6] and the alteration p.Gly184Arg, already described in a Brazilian family [7], was found in an unrelated patient. Among patients with already known mutations, only the carrier of p.Gly348X reported family history of HAE. Seven patients were diagnosed only after 30 years old with a delay in diagnosis ranging from 16 to 44 years.

**Conclusions:** In summary, we report here 10 cases of unrelated patients diagnosed with HAE for the first time in their families, within six new alterations inC1-INH gene. Due to the lack of family history in eight cases and the absence of the correct diagnosis in affected relatives in the other two cases, the molecular analysis of *SERPING1* was decisive in the final diagnosis. Although most physicians do not appreciate the need of molecular sequencing in all cases of HAE [8], it is a special tool in difficult to diagnose HAE patients. It also allows genetic counselling and increases knowledge regarding the mutational spectrum of *SERPING1*.

**Acknowledgements:** We thank Priscila A. Nicolicht for the technical help.

**References**

1. Landerman NS, Webster ME, Becker EL, Ratcliffe HE. Hereditary angioneurotic edema. II. Deficiency of inhibitor for serum globulin permeability factor and/or plasma kallikrein. J Allergy. 1962;33:330–41.

2. Donaldson VH, Evans RR. A biochemical abnormality in hereditary angioneurotic edema: absence of serum inhibitor of C1-esterase. Am J Med. 1963;35:37–44.

3. Cicardi M, Igarashi T, Kim MS, Frangi D, Agostoni A, Davis AE 3rd. Restriction fragment length polymorphism of the C1 inhibitor gene in hereditaryangioneurotic edema. J Clin Invest. 1987;80(6):1640–3.

4. Stoppa-Lyonnet D, Tosi M, Laurent J, Sobel A, Lagrue G, Meo T. Altered C1 inhibitor genes in type I hereditary angioedema. N Engl J Med. 1987;317(1):1–6.

5. Pappalardo E, Caccia S, Suffritti C, Tordai A, Zingale LC, Cicardi M. Mutation screening of C1 inhibitor gene in 108 unrelated families with hereditary angioedema: functional and structural correlates. Mol Immunol. 2008;45(13):3536–44.

6. Gössewein T, Koccot A, Emmert G, et al. Mutational spectrum of the C1INH (SERPING1) gene in patients with hereditary angioedema. Cytogenet Genome Res. 2008;121:181–8.

7. Cagini N, Veronez CL, Constantino-Silva RN, Buzolin M, Martin RP, Grumach AS, Velloso LA, Mansour E, Pesquero JB. New mutations in SERPING1 gene of Brazilian patients with hereditary angioedema. Biol Chem. 2016;397(4):337–44.

8. Speletas M, Szilagyi A, Psarros F, Moldovan D, Magerl M, Kompoti M, Gramoustianou E, Bors A, Mihaly E, Tordai A, Avramouli A, Varga L, Maurer M, Farkas H, Germenis AE. Hereditary angioedema: molecular and clinical differences among European populations. J AllergyClin Immunol. 2015;135(2):570–3.

### P-42 Clinical and analytical characteristics in children with hereditary angioedema due to C1-inhibitor deficiency

#### Maria Pedrosa^1^, Teresa González-Quevedo^2^, Carmen Marcos^3^, Teófilo Lobera^4^, María L. Baeza^5,6^, Blanca Sáenz de San Pedro^7^, Teresa Caballero^1,8^

##### ^1^Department of Allergy, Instituto de Investigación Hospital Universitario La Paz (IdiPAZ), Madrid, Spain; ^2^Department of Allergy, Hospital Universitario Virgen del Rocío, Sevilla, Spain; ^3^Department of Allergy, Complejo Hospitalario Universitario de Vigo, Vigo, Spain; ^4^Department of Allergy, Hospital San Pedro, Logroño, Spain; ^5^Department of Allergy, Hospital General Universitario Gregorio Marañón, Madrid, Spain; ^6^CIBERER U761, Madrid, Spain; ^7^Department of Allergy, Hospital Universitario de Jaén, Jaén, Spain; ^8^CIBERER U754, Madrid, Spain

###### **Correspondence:** Teresa Caballero (mteresa.caballero@idipaz.es)

*Allergy, Asthma & Clinical Immunology* 2017,** 13(Suppl 2)**:P-42

**Objective:** To describe clinical and analytical characteristics of a paediatric population of patients with angioedema due to C1-inhibitor deficiency (C1-INH-HAE).

**Methods:** We performed a retrospective review of clinical charts of patients aged less than 18 years old diagnosed with C1-INH-HAE, regularly followed up in the Outpatient Clinics of the Departments of Allergy belonging to SGBA (Spanish Study Group on Bradykinin-induced Angioedema).

**Results:** Forty-five patients were included (55.6% males). The median age at diagnosis was 3.67 years (IQR 1.04–7.41 year). The median age at onset of symptoms was 6.6 years (IQR 3.67–8.75 year). The diagnosis preceeded symptoms in 29 patients (64.4%). The median delay in diagnosis was 2 months (IQR 0–25 month). Thirteen patients (28.9%) presented their debut before the age of 6 years, 16 (35.6%) between 6–12 years and 2 (4.4%) between 12 and 18 years. Fourteen patients (31.1%) remain asymptomatic. The most frequent location of the first episode was peripheral in 16 patients (51.6%), followed by abdominal and facial (N = 4, 12.9%). Twelve patients had a known trigger, the most frequent being trauma (N = 7, 22.5%). Thirty-nine percent of the symptomatic patients are under long term prophylaxis (LTP). The median reduction of episodes after LTP was 50% (IQR −50 to −80.7%). More than half of the patients (51.6%) did not receive treatment for their first episode. Nevertheless, 77.4% of the patients had replacement with plasma derived C1INH concentrate for subsequent attacks. No viral transmission has been documented. There was no correlation between the age at onset of symptoms or the levels of complement and the number of presented episodes. C1-INH and C4 levels were significantly lower in symptomatic patients (p = 0.019 y 0.028).

**Conclusions:** The majority of children with C1-INH-HAE are diagnosed before presenting symptoms. Less than a half need LTP. The most frequent location of attacks is peripheral. There is no correlation between neither the age at onset of symptoms nor the levels of complement with the severity of the disease.

### P-43 Hereditary angioedema rapid triage (HAE-RT) tool: a Delphi study

#### Lisa Fu^1^, Stephen Betschel^1^, Ernie Avilla^2^, Jacquie Badiou^3^, Karen Binkley^1^, Rozita Borici-Mazi^4^, Jacques Hebert^5^, Linda Howlett^3^, Amin Kanani^6^, Paul K. Keith^7^, Gina Lacuesta^8^, Anne Rowe^3^, Peter Waite^9^, Susan Waserman^3^

##### ^1^Department of Medicine, Division of Clinical Immunology and Allergy, University of Toronto, Toronto, ON, M5B 1W8, Canada; ^2^IDEA Group, Hamilton, ON, L8S 3C2, Canada; ^3^HAE Canada, Ottawa, ON, K1Y 4G2, Canada; ^4^Division of Allergy and Immunology Queens University, Kingston, ON, K7L 5G2, Canada; ^5^Centre de Recherche Appliquée en Allergie de Québec, QC, G1V 4W2, Canada; ^6^Division of Allergy & Clinical Immunology, St. Paul’s Hospital, Vancouver, BC, V6Z 1Y6, Canada; ^7^Division of Clinical Immunology and Allergy, Department of Medicine, McMaster University, Hamilton, ON, L8S 4K1, Canada; ^8^Department of Medicine, Dalhousie University, Halifax, NS, B3H 4R2, Canada; ^9^Canadian Hereditary Angioedema Network, Toronto, ON, M5B 2H5, Canada

###### **Correspondence:** Lisa Fu (lisa.wy.fu@gmail.com)

*Allergy, Asthma & Clinical Immunology* 2017,** 13(Suppl 2)**:P-43

**Background:** HAE patients (both diagnosed and undiagnosed) commonly present to the emergency department (ED). Presenting symptoms (swelling and pain) may be erroneously attributed to common allergic and gastrointestinal conditions resulting in major delays in diagnosis and appropriate treatment. No published tools currently exist for HAE screening and management in undiagnosed disease. The overall goal of the study was to develop and validate a HAE-RT tool for ED settings.

**Methods:** A Delphi study process was used with three rounds of ratings, consisting of a structured questionnaire followed by a facilitated discussion, in order to reach consensus on the final components of the HAE-RT Tool. We determined *a priori* that we would prioritize items for modification in cases where agreement was not achieved by 80% of respondents or the median score is <5 out of 7. If subsequent round of ratings were needed, panel members were asked to reassess their agreement on concepts based on responses of the panel, and come to a consensus. *Participants:* HAE specialists (N = 9) and National Patient Advocacy Group Members (N = 3).

**Results:** Of the 12 experts invited, 9 (75% response rate, 7 allergists, 2 patients) agreed to contribute to the Delphi study questionnaire. The questionnaire addressed 6 key areas required for HAE-RT tool development: (A) Objectives; (B) Impact on clinical practice; (C) Target population; (D) HAE-specific clinical findings; (E) HAE specific treatment options and; (F) ED discharge options. Of 18 statements, 13 (72%) achieved consensus, 5 (27%) did not. Of the 5 statements not reaching consensus, 1 was on stated objectives of the tool and 4 were statements on HAE-specific clinical findings. The expert panellists were asked to discuss and reassess their agreement until a consensus was reached on all 5 statements.

**Conclusion:** Our Delphi study emphasizes the importance of expert involvement and feedback to facilitate the prioritization of important information that must be included in the design of an HAE-RT tool.

### P-44 The effect of menopause on hereditary angioedema: a descriptive study about 48 postmenopausal French patients

#### Aurore Billebeau^1^, Olivier Fain^2,5^, David Launay^3,5^, Isabelle Boccon-Gibbod^4,5^, Laurence Bouillet^4,5^, Anne Gompel^1,5^

##### ^1^Department of Endocrinology Gynecology, Paris Descartes University, Paris, 75014, France; ^2^Department of Internal Medicine, Paris Pierre et Marie Curie University, Paris, 75012, France; ^3^Department of Internal Medicine, Lille Nord de France University, Lille, 59000, France; ^4^Department of Internal Medicine, Grenoble Alpes University, Grenoble, 38400, France; ^5^French National Reference Center for Hereditary Angioedema (CREAK), Grenoble, France

###### **Correspondence:** Aurore Billebeau (aurore.crastes@gmail.com)

*Allergy, Asthma & Clinical Immunology* 2017,** 13(Suppl 2)**:P-44

**Background:** All types of hereditary angioedema (HAE) can be worsened by estrogens. Attenuated androgens are used as treatment in HAE with C1inhibitor (C1INH) deficiency. Menopause is associated with an extinction of estrogenic secretion but also of the ovarian androgens.

**Objective:** The objective of this study was to describe the effect of menopause on hereditary angioedema attacks.

**Methods:** We conducted a retrospective, multi-center study in postmenopausal French women with non-allergic angioedema, including HAE with C1 inhibitor deficiency (C1-INHHAE), and hereditary angioedema with normal C1 inhibitor and factor XII mutation or without factor XII mutation but with family history. The disease severity score previously described [1] was used to classify patients before and after menopause. The influence of hormonal factors on the course of HAE during childbearing years was also assessed.

**Results:** We included 48 postmenopausal women, from 13 centers in France. The mean age was 63.2 years (SD 10.1), and the mean time between menopause and inclusion was 12.9 years (SD 9.8). Eighty-nine percent (N = 43) of patients had C1-INH-HAE, 4% (n = 2) had a type 3 hereditary angioedema with mutation of factor XII and 6% (n = 3) had a type 3 hereditary angioedema without mutation of factor XII but with family history. Concerning the effect of menopause on HAE, 38% (=18) improved after menopause, 23% (n = 11) worsened, and 40% (n = 19) did not record any modification. Three among 9 women who used it worsened with Menopause hormonal therapy (MHT). For 74.4% (n = 35) of women, fluctuations of estrogen (puberty, combined oral contraceptives, pregnancy and/or hormonal replacement therapy) triggered angioedema attacks. At inclusion, 57.4% (n = 27) of women had a long-term prophylactic treatment.

**Conclusion:** This study shows that menopause has a non predictive effect on HAE course. This suggests that a systematic study on the hormone imbalance in these patients could be helpful to improve their management in the post menopause.

**Acknowledgements:** We thank for their participation: Dr. S. Amarger, Dr. C. Blanchard-Delaunay, Dr. A. DuThanh, Dr. B. Coppere, Dr. B. Floccard, Dr. S. Gayet, Dr. P. Y. Jeandel, Pr L. Martin, Dr. F. Pelletier, Dr. F. N. Raison Peyron, Dr. S. Trouillet and the French National Reference Center for Hereditary Angioedema (CREAK).

**Reference**

1. Prior N, Caballero T. J Allergy Clin Immunol Pract. 2016;4(3):464–73.

### P-45 Normal C1-INH angioedema in Israel: phenotyping and *F12* gene sequencing

#### Avner Reshef^1^, Iris Leibovich^1^, Kristina Lis^1^, Yael Laitman^2^, Eitan Friedman^2^

##### ^1^Angioedema Center, The Sheba Medical Center, Tel-Hashomer, 5265601, Israel; ^2^Laboratory of Molecular Genetics, Institute of Human Genetics, The Sheba Medical Center, Tel-Hashomer, 5265601, Israel

###### **Correspondence:** Avner Reshef (avner.reshef@sheba.health.gov.il)

*Allergy, Asthma & Clinical Immunology* 2017,** 13(Suppl 2)**:P-45

**Background:** A subset of angioedema patients with normal C1-INH levels exhibit mutations in coagulation Factor XII gene (*F12*). The role of *F12* mutations and underlying pathophysiology of this HAE type are poorly understood, but the clinical expressions are similar to C1-INH-HAE [1–3]. *F12* gene is mapped to chromosome 5 (5q35.3) and comprise 13 introns and 14 exons covering 12 kb. Currently all known *F12* gene mutations (HAE3, OMIM #610618) are located on exon 9 in the proline-rich linker peptide between the Kringle and trypsin-like serine protease (Tryp-SPc) domains. Two missense mutations (Thr309Lys, Thr309Arg) are the most common [1, 2]. Recently two new *F12* mutations (duplication and deletion) were described in patients with idiopathic non-histaminergic angioedema, as well as in patients with FXII deficiency [4].

**Methods:** Our normal C1-INH population comprised 59 pts (57 females, 3 males). All had clinical history of recurrent angioedema attacks, without hives, normal laboratory values of C4, antigenic and functional C1-INH. A 37-item questionnaire was developed to further characterize the phenotypic expression of these patients. Blood samples of 38 pts were analyzed by *F12* gene sequencing. DNA was extracted manually from whole blood using the Promega Wizard Genomic DNA Purification Kit. Four mutations within exon 9 of the *F12* gene, namely: Thr328Lys, Thr328Arg, c.971del72 and c.892dup18, were analyzed by agarose gel separation and direct Sanger sequencing.

**Results:** 38 patients could be analyzed (36 females, 2 male), Mean age 37.7 ± 12.6 years, (range 16–60). Clinical manifestations included angioedema in various locations, with some patients having more than 25 attacks per year. Mean age of onset of angioedema was 23.9 ± 10.4 years (range 7–53) and 20.5 ± 4.0 years for patients with positive *F12* mutation pts (range 15–29). The *F12* mutation Thr328Lys was found in 16 pts (42.1%, all females). This mutation was found in 9/10 from the same family, 2/2 from another family, and 1 member of the other 5 families. Other F12 mutations were not expressed in this cohort.

**Conclusions:** This is the first phenotype analysis and genetic mapping of *F12* in normal C1-INH in Israel. The study shows that 16/38 patients could be classified as FXII-HAE. Genetic analysis of other patients and normal siblings is underway.

**References**

1. Dewald G, et al. Biochem Biophys Res Commun. 2006;343:1286–89.

2. Bork K, et al. Clin Immunol. 2011;141:31–5.

3. Bork K, et al. Am J Med. 2013;126:1142.e9-14

4. Gelincik A, et al. Ann Allergy Asthma Immunol. 2015;114:154–56.

### P-46 The determinants of hereditary angioedema disease severity: geno-phenotypical aspects

#### N. M. Gokmen, O. Gulbahar, H. Onay, Z. P. Koc, A. Z. Sin

##### Immunology, Ege University, Izmir, 34437, Turkey

###### **Correspondence:** N. M. Gokmen (outkongre@vikingturizm.com.tr)

*Allergy, Asthma & Clinical Immunology* 2017,** 13(Suppl 2)**:P-46

**Background:** Hereditary angioedema with C1 inhibitor deficiency (C1-INH-HAE) is a rare autosomal dominant disorder that manifests as negligible symptoms to life-threatening recurrent episodes of swelling involving the skin, gastrointestinal tract and upper airways. The determinants of disease symptom severity were not clearly clarified yet. We aimed to investigate the potential correlation between geno-phenotypical characteristics and the parameters of severe disease course of HAE patients.

**Methods:** The coding exons and the exon-intron boundaries of the *SERPING1* gene were sequenced and in case of no mutation deletion/duplication analysis with Multiple Ligation Dependent Probe Amplification (MLPA) was performed, and clinical data were obtained in 81 symptomatic HAE patients from 47 unrelated families at the time of diagnosis. Treatment-free blood samples for C1 inhibitor function, C1 inhibitor antigen and C1q levels were taken in 61 patients.

**Results:** Thirty-five different (15 novel and 2/15 homozygous) mutations were identified. There was no causative mutation in 6 out of 81 (7.4%) patients. The lowest C1 inhibitor function levels was detected in patients with deletion (5.05%), and Large Deletion (5.8%) type mutation; whereas the highest C1 inhibitor function was present in patients with none mutation (23.3%; p = 0.02). C1 inhibitor function levels were correlated with disease onset age (r = 0.278, p = 0.03) and symptom severity scores (r = −0.404, p = 0.001), lifelong laryngeal attack numbers (r = −0.404, p < 0.001), annual laryngeal attacks frequency (r = −0.353, p < 0.005), frequency of annual attack (r = −0.289; p < 0.024). Patients with earlier disease onset age, had more frequent annual attacks in every localization and annual abdominal attacks (r = −0.420, p < 0.0001 and r = −0.371, p < 0,001, respectively).

**Conclusion:** The higher symptom severity, the more frequent lifelong/annual laryngeal attack and annual attacks in every localizations seem to be occur in patients with lower level of C1 inhibitor function and with earlier disease onset age. Deletion and Large deletion type mutation can be result in most unfavourable, whereas none mutation group had most favourable C1 inhibitor function levels.

